# A CYP78As–small grain4–coat protein complex Ⅱ pathway promotes grain size in rice

**DOI:** 10.1093/plcell/koad239

**Published:** 2023-09-21

**Authors:** Chunlei Zhou, Qibing Lin, Yulong Ren, Jie Lan, Rong Miao, Miao Feng, Xin Wang, Xi Liu, Shengzhong Zhang, Tian Pan, Jiachang Wang, Sheng Luo, Jinsheng Qian, Wenfan Luo, Changling Mou, Thanhliem Nguyen, Zhijun Cheng, Xin Zhang, Cailin Lei, Shanshan Zhu, Xiuping Guo, Jie Wang, Zhichao Zhao, Shijia Liu, Ling Jiang, Jianmin Wan

**Affiliations:** State Key Laboratory for Crop Genetics and Germplasm Enhancement, Jiangsu Plant Gene Engineering Research Center, Nanjing Agricultural University, Nanjing 210095, China; State Key Laboratory of Crop Gene Resources and Breeding, Institute of Crop Sciences, Chinese Academy of Agricultural Sciences, Beijing 100081, China; State Key Laboratory of Crop Gene Resources and Breeding, Institute of Crop Sciences, Chinese Academy of Agricultural Sciences, Beijing 100081, China; State Key Laboratory of Crop Gene Resources and Breeding, Institute of Crop Sciences, Chinese Academy of Agricultural Sciences, Beijing 100081, China; State Key Laboratory for Crop Genetics and Germplasm Enhancement, Jiangsu Plant Gene Engineering Research Center, Nanjing Agricultural University, Nanjing 210095, China; State Key Laboratory for Crop Genetics and Germplasm Enhancement, Jiangsu Plant Gene Engineering Research Center, Nanjing Agricultural University, Nanjing 210095, China; State Key Laboratory of Crop Gene Resources and Breeding, Institute of Crop Sciences, Chinese Academy of Agricultural Sciences, Beijing 100081, China; State Key Laboratory of Crop Gene Resources and Breeding, Institute of Crop Sciences, Chinese Academy of Agricultural Sciences, Beijing 100081, China; State Key Laboratory for Crop Genetics and Germplasm Enhancement, Jiangsu Plant Gene Engineering Research Center, Nanjing Agricultural University, Nanjing 210095, China; State Key Laboratory for Crop Genetics and Germplasm Enhancement, Jiangsu Plant Gene Engineering Research Center, Nanjing Agricultural University, Nanjing 210095, China; State Key Laboratory for Crop Genetics and Germplasm Enhancement, Jiangsu Plant Gene Engineering Research Center, Nanjing Agricultural University, Nanjing 210095, China; State Key Laboratory for Crop Genetics and Germplasm Enhancement, Jiangsu Plant Gene Engineering Research Center, Nanjing Agricultural University, Nanjing 210095, China; State Key Laboratory of Crop Gene Resources and Breeding, Institute of Crop Sciences, Chinese Academy of Agricultural Sciences, Beijing 100081, China; State Key Laboratory of Crop Gene Resources and Breeding, Institute of Crop Sciences, Chinese Academy of Agricultural Sciences, Beijing 100081, China; State Key Laboratory of Crop Gene Resources and Breeding, Institute of Crop Sciences, Chinese Academy of Agricultural Sciences, Beijing 100081, China; State Key Laboratory for Crop Genetics and Germplasm Enhancement, Jiangsu Plant Gene Engineering Research Center, Nanjing Agricultural University, Nanjing 210095, China; State Key Laboratory for Crop Genetics and Germplasm Enhancement, Jiangsu Plant Gene Engineering Research Center, Nanjing Agricultural University, Nanjing 210095, China; State Key Laboratory of Crop Gene Resources and Breeding, Institute of Crop Sciences, Chinese Academy of Agricultural Sciences, Beijing 100081, China; State Key Laboratory of Crop Gene Resources and Breeding, Institute of Crop Sciences, Chinese Academy of Agricultural Sciences, Beijing 100081, China; State Key Laboratory of Crop Gene Resources and Breeding, Institute of Crop Sciences, Chinese Academy of Agricultural Sciences, Beijing 100081, China; State Key Laboratory of Crop Gene Resources and Breeding, Institute of Crop Sciences, Chinese Academy of Agricultural Sciences, Beijing 100081, China; State Key Laboratory of Crop Gene Resources and Breeding, Institute of Crop Sciences, Chinese Academy of Agricultural Sciences, Beijing 100081, China; State Key Laboratory of Crop Gene Resources and Breeding, Institute of Crop Sciences, Chinese Academy of Agricultural Sciences, Beijing 100081, China; State Key Laboratory of Crop Gene Resources and Breeding, Institute of Crop Sciences, Chinese Academy of Agricultural Sciences, Beijing 100081, China; State Key Laboratory for Crop Genetics and Germplasm Enhancement, Jiangsu Plant Gene Engineering Research Center, Nanjing Agricultural University, Nanjing 210095, China; State Key Laboratory for Crop Genetics and Germplasm Enhancement, Jiangsu Plant Gene Engineering Research Center, Nanjing Agricultural University, Nanjing 210095, China; State Key Laboratory for Crop Genetics and Germplasm Enhancement, Jiangsu Plant Gene Engineering Research Center, Nanjing Agricultural University, Nanjing 210095, China; State Key Laboratory of Crop Gene Resources and Breeding, Institute of Crop Sciences, Chinese Academy of Agricultural Sciences, Beijing 100081, China

## Abstract

CYP78A, a cytochrome P450 subfamily that includes rice (*Oryza sativa* L.) BIG GRAIN2 (BG2, CYP78A13) and *Arabidopsis thaliana* KLUH (KLU, CYP78A5), generate an unknown mobile growth signal (referred to as a CYP78A-derived signal) that increases grain (seed) size. However, the mechanism by which the CYP78A pathway increases grain size remains elusive. Here, we characterized a rice small grain mutant, *small grain4* (*smg4*), with smaller grains than its wild type due to restricted cell expansion and cell proliferation in spikelet hulls. *SMG4* encodes a multidrug and toxic compound extrusion (MATE) transporter. Loss of function of *SMG4* causes smaller grains while overexpressing *SMG4* results in larger grains. SMG4 is mainly localized to endoplasmic reticulum (ER) exit sites (ERESs) and partially localized to the ER and Golgi. Biochemically, SMG4 interacts with coat protein complex Ⅱ (COPⅡ) components (Sar1, Sec23, and Sec24) and CYP78As (BG2, GRAIN LENGTH 3.2 [GL3.2], and BG2-LIKE 1 [BG2L1]). Genetically, *SMG4* acts, at least in part, in a common pathway with *Sar1* and *CYP78A*s to regulate grain size. In summary, our findings reveal a CYP78As–SMG4–COPⅡ regulatory pathway for grain size in rice, thus providing new insights into the molecular and genetic regulatory mechanism of grain size.

## Teaser

SMG4 interacts with CYP78As and COPⅡ components, acting in a common pathway with them to regulate rice grain size.

**IN A NUTSHELL**

**Background:** Grain size is a key factor for determining grain yield. Many genes or quantitative trait loci (QTLs) that regulate grain size have been identified. Among them, members of the CYP78A cytochrome P450 subfamily are conserved regulators of grain (seed) size in plants. However, the relation between CYP78As and other grain size regulators is largely unknown. Although CYP78As have been reported to likely function by generating a mobile growth signal, the underlying molecular mechanism remains elusive.

**Question:** What is the regulatory pathway of grain size involving CYP78As? What is the molecular and genetic mechanism behind it?

**Findings:** Here, we identified a MATE transporter, SMALL GRAIN 4 (SMG4), which regulates grain size in rice. Loss of function of *SMG4* causes smaller grains while overexpressing *SMG4* results in larger grains. SMG4 is mainly localized to ER exit sites (ERESs) and partially localized to the endoplasmic reticulum and Golgi. Biochemical assays showed that SMG4 interacts with CYP78As (BG2, GRAIN LENGTH 3.2 [GL3.2], and BG2-LIKE 1 [BG2L1]) and COPⅡ components (Sar1, Sec23, and Sec24). Genetic analyses suggest that *CYP78As*, *SMG4*, and *Sar1* likely act, at least in part, in a common pathway to regulate grain size. Taken together, our findings reveal that a CYP78As–SMG4–COPⅡ pathway promotes grain size in rice, thus providing a new strategy for improving grain size and yield in crops.

**Next step:** We will further investigate the nature of the CYP78A-derived signal transported by SMG4 and reveal how it is transmitted.

## Introduction

As a staple crop, rice (*Oryza sativa* L.) feeds more than half the world's population. To meet the demands of a growing global population, there is a need to increase rice yield. Rice yield is largely determined by the panicle number, grain number per panicle, and grain weight (Xing and Zhang 2010). Grain size, the main determinant of grain weight, is specified by grain length, width, and thickness. Many genes or quantitative trait loci (QTLs) that modulate rice grain size and shape have been identified (Fan et al. 2006; Song et al. 2007; Shomura et al. 2008; Li et al. 2011; Chen et al. 2015; Wang et al. 2015a; Xu et al. 2015; Liu et al. 2017; Hu et al. 2018; Liu et al. 2018; Zhao et al. 2018; Li et al. 2019; Shi et al. 2019). Among them, *BIG GRAIN2* (*BG2*) encodes a cytochrome P450 monooxygenase belonging to the CYP78A subfamily (Xu et al. 2015). CYP78As play an important role in promoting organ size in plants (Ito and Meyerowitz 2000; Adamski et al. 2009; Chakrabarti et al. 2013; Ma et al. 2015; Wang et al. 2015b; Xu et al. 2015; Zhao et al. 2016; Qi et al. 2017). In Arabidopsis (*Arabidopsis thaliana*), ENHANCER OF DA1-1 3 (EOD3, also named CYP78A6) functions redundantly with CYP78A9 to regulate seed size (Fang et al. 2012); another CYP78A, KLUH (KLU, also named CYP78A5) is thought to generate a mobile signal that promotes inflorescence and seed growth in a non-cell-autonomous manner (Anastasiou et al. 2007; Adamski et al. 2009). The *klu* mutant phenotype can be rescued by expressing rice *BG2* (*CYP78A13*), which is reported to control grain size in rice (Xu et al. 2015). These studies suggest that members of the CYP78A subfamily likely generate growth signals that enhance fruit and seed (grain) development in plants. However, the regulatory pathway behind grain size that involves CYP78As is still unknown.

The multidrug and toxic compound extrusion (MATE) family of transporters is present in almost all organisms, including prokaryotes and eukaryotes (Wang et al. 2016a; Upadhyay et al. 2019). Compared to bacteria and animals, plants contain many more MATE transporters (Li et al. 2002), and these transporters participate in diverse physiological aspects, including secondary metabolite transport, disease resistance, and iron homeostasis (Marinova et al. 2007; Yokosho et al. 2009; Dobritzsch et al. 2016; Upadhyay et al. 2019). Several lines of evidence indicate that MATE transporters are also involved in seed development. For example, the *big embryo 1a* (*bige1a*) mutant has smaller seeds than the wild type (WT) in Arabidopsis (Suzuki et al. 2015). The maize (*Zea mays*) BIGE1 ortholog controls embryo size by regulating cell expansion of the scutellum (Suzuki et al. 2015), and defective grain-filling 1 (DG1) in rice acts as an abscisic acid (ABA) transporter that controls grain development in a temperature-dependent manner (Qin et al. 2021). However, the molecular mechanism by which MATEs regulate grain size remains largely unclear.

Vesicle trafficking of secretory cargoes from the endoplasmic reticulum (ER) to the Golgi is a vital cellular program in all eukaryotes (D’Arcangelo et al. 2013; Chung et al. 2016). ER export is mainly mediated by coat protein complex Ⅱ (COPⅡ) vesicles, which comprise 5 conserved components: Sar1, Sec23, Sec24, Sec13, and Sec31 (D’Arcangelo et al. 2013; Chung et al. 2016). The assembly of the COPⅡ coat on the ER membrane is initiated by Sec12, a guanine nucleotide exchange factor (Barlowe and Schekman 1993). The small GTPase Sar1 is activated by Sec12 and is inserted into the ER membrane. Activated Sar1 then recruits an inner-coat complex (composed of Sec23 and Sec24) to form a prebudding complex, which subsequently recruits an outer-coat complex (composed of Sec13 and Sec31) that drives vesicle release from the ER membrane (Bi et al. 2002; Miller et al. 2002; Stagg et al. 2006; Chung et al. 2016). As a transporter of secretory cargoes, COPⅡ can interact with different cargo receptors. Several cargo receptors have been reported, such as yeast (*Saccharomyces cerevisiae*) ER-vesicle protein of 29 kD (Erv29p), which facilitates the trafficking of multiple soluble cargo proteins (Belden and Barlowe 2001), and mammalian ER-Golgi intermediate compartment 53 kD (ERGIC-53) family members, which mediate the trafficking of glycoproteins (Dancourt and Barlowe 2010). Previous studies have shown that COPⅡ components can affect plant fertility and grain quality (Tian et al. 2013; Aboulela et al. 2018; Liang et al. 2020; Bao et al. 2023). However, the role of COPⅡ in regulating grain size remains unclear.

In this study, we isolated and characterized the rice *small grain 4* (*smg4*) mutant, which exhibits a small grain phenotype. *SMG4* encodes a MATE transporter that promotes grain growth by positively regulating cell expansion and cell proliferation in spikelet hulls. Further, we showed that SMG4 is mainly localized to ER exit sites (ERESs) and partially localized to the ER and Golgi. Furthermore, SMG4 not only interacted with COPⅡ components but also with CYP78As. Genetic analyses indicate that CYP78As, SMG4, and COPⅡ function in a common pathway to regulate grain size. These findings establish a CYP78As–SMG4–COPⅡ pathway that promotes grain size in rice, thus providing potential targets for improving grain size.

## Results

### Phenotypic characterization of the *smg4* mutant

**Figure 1.**
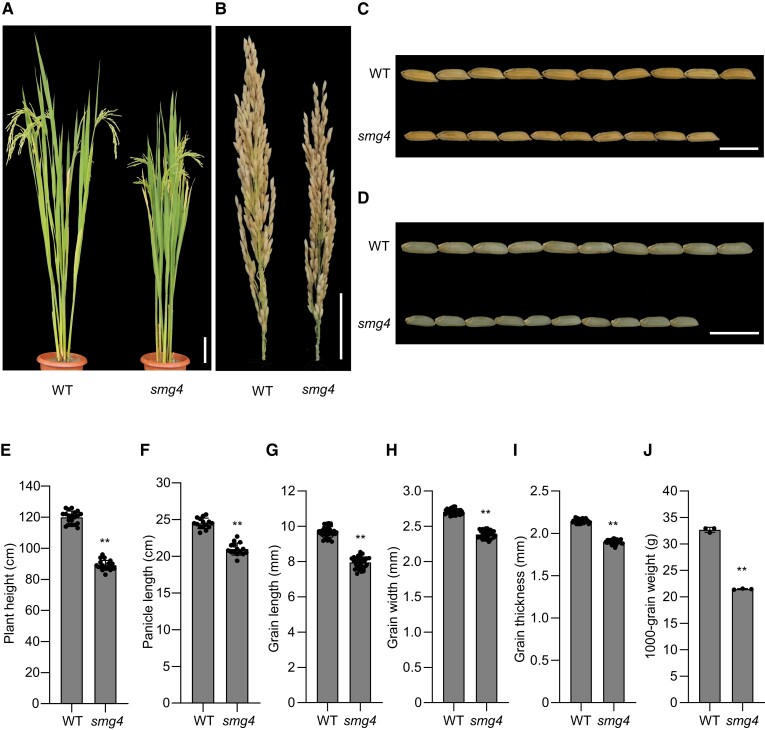
Phenotypes of the *smg4* mutant. **A)** Plant architecture of wild-type (WT) and *smg4* mutant plants at the mature stage. Scale bar, 10 cm. **B)** Mature panicles of WT and *smg4*. Scale bar, 5 cm. **C)** Mature rice grains of WT and *smg4*. Scale bar, 1 cm. **D)** Brown rice grains of WT and *smg4*. Scale bar, 1 cm. **E)** to **(J)** plant height (*n* = 20) **(E)**, panicle length (*n* = 15) **(F)**, grain length (*n* = 30) **(G)**, grain width (*n* = 30) **(H)**, grain thickness (*n* = 30) **(I)**, and thousand-grain weight (*n* = 3) **(J)** in WT and *smg4*. Values are means ± SD. Student's *t*-test was used to generate the *P*-values, **P < 0.01.

To identify genes involved in the regulation of grain size in rice, we isolated the ethyl methanesulfonate (EMS)-mutagenized small grain mutant *smg4* from the *indica* cultivar 9311. Compared to the WT, *smg4* had shorter plants, shorter panicles, and clearly smaller grains with smaller brown grains (Fig. 1, A–F). Further observations showed that the smaller grain size of *smg4* is caused by smaller grain length, width, and thickness compared to WT (Fig. 1, G–I). Accordingly, the thousand-grain weight of the mutant decreased by 34.3% relative to WT (Fig. 1J), and the brown grain length, width, and thickness of *smg4* were also significantly smaller (Supplemental Table S1). However, tiller number, primary branch number, secondary branch number, and grain number per main panicle in *smg4* were comparable to those of WT (Supplemental Table S1). A time-course measurement of brown grain dry weight showed that *smg4* has a lower grain filling rate, especially during the late filling stage, compared to WT (Supplemental Fig. S1), indicating that SMG4 promotes rice grain filling. These results suggest that SMG4 regulates grain size and grain weight in rice.

### *SMG4* promotes cell expansion and cell proliferation in the spikelet hull

**Figure 2.**
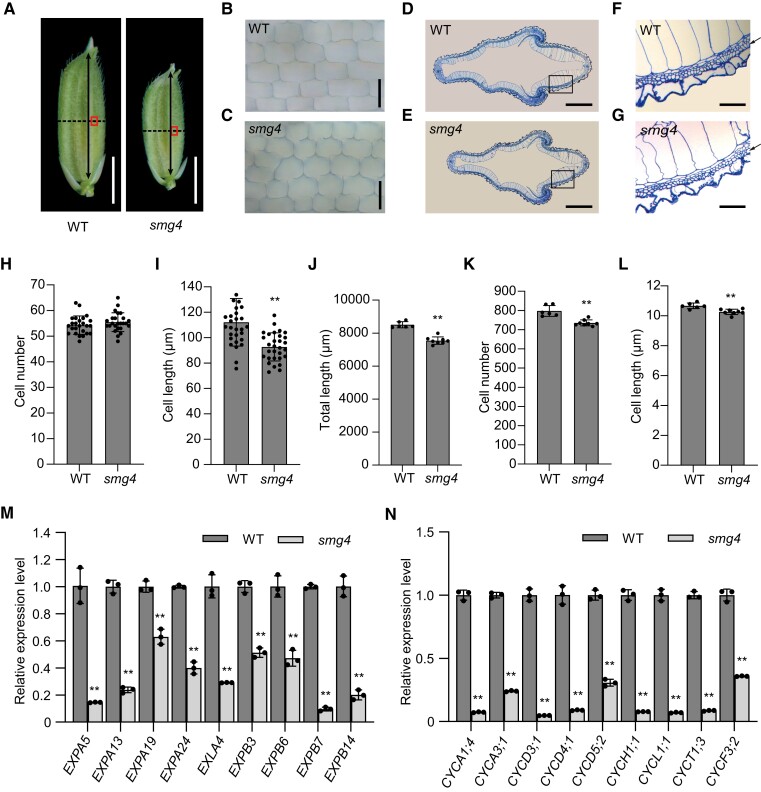
*SMG4* promotes cell expansion and cell proliferation. **A)** Spikelet hulls of WT and *smg4* just before a thesis. The boxes represent the observation sites of **(B)** and **(C)**. The dotted lines indicate the sites of cross sections in **(D)** and **(E)**. The double-headed arrows indicate the orientation for the cell number statistics in **(H)**. Scale bars, 3 mm. **B, C)** Microscopy observation of WT **(B)** and *smg4***(C)** inner epidermal cells. Scale bars, 100 *µ*m. **D, E)** Cross sections of WT **(D)** and *smg4***(E)** spikelet hulls. Scale bars, 500 *µ*m. **F, G)** The 5× enlargement of the regions outlined by the box in **(D)** and **(E)**, respectively. The arrows indicate the outer cell layers that were compared in **(J)** to **(L)**. Scale bars, 100 *µ*m. **H, I)** Longitudinal cell number (*n* = 25) **(H)** and cell length (*n* = 30) **(I)** of inner epidermal cells in WT and *smg4*. **J–L)** Total cell length (*n* ≥ 6) **(J)** cell number (*n* ≥ 6) **(K)** and cell length of each cell (*n* ≥ 6) **(L)** in the outer parenchyma cell layer in WT and *smg4*. **M, N)** Relative expression levels of cell expansion-related genes **(M)** and cell cycle-related genes **(N)** in spikelet hulls BH of WT and *smg4* (*n* = 3). The *UBIQUITIN* gene was used as an internal control. Values are means ± SD. Student's *t*-test was used to calculate the *P*-values, **P < 0.01.

The spikelet hull size, delimiting the final size of a grain, is determined by cell expansion and cell proliferation (Li and Li 2016). Compared to the WT, the spikelet hull of *smg4* was shorter and narrower (Fig. 2A). Next, we investigated the cellular mechanism by which SMG4 regulates grain size. We performed microscopy observations, which revealed that the longitudinal cell length of *smg4* inner epidermal cells is significantly smaller, while the number of longitudinal inner epidermal cells was comparable to that of WT (Fig. 2, B–H and I). Furthermore, scanning electron microscopy analysis showed that the longitudinal cell length of both the outer and inner epidermal cells of mature grains is significantly decreased in *smg4* compared to WT (Supplemental Fig. S2). Thus, the shorter grain of *smg4* is likely caused by diminished longitudinal cell length in the spikelet hull. In addition, we compared cross sections of the middle parts of spikelet hulls from WT and *smg4* (Fig. 2, D–G and J–L). We observed that the transverse outer parenchyma cell layer in *smg4* is shorter than that of WT (Fig. 2J), and the number and length of transverse outer parenchyma cells in *smg4* are 7.8% and 3.8% lower, respectively, than that of WT (Fig. 2, K and L), suggesting that the narrower grains of *smg4* are caused by their smaller transverse cell number and cell length. Supporting these results, expression levels of 9 cell expansion-related genes and 9 cell cycle-related genes were significantly lower in the spikelet hulls of *smg4* compared to WT (Fig. 2, M and N). These results indicate that SMG4 positively regulates grain size by promoting cell expansion and cell proliferation.

### *SMG4* encodes a MATE transporter

**Figure 3.**
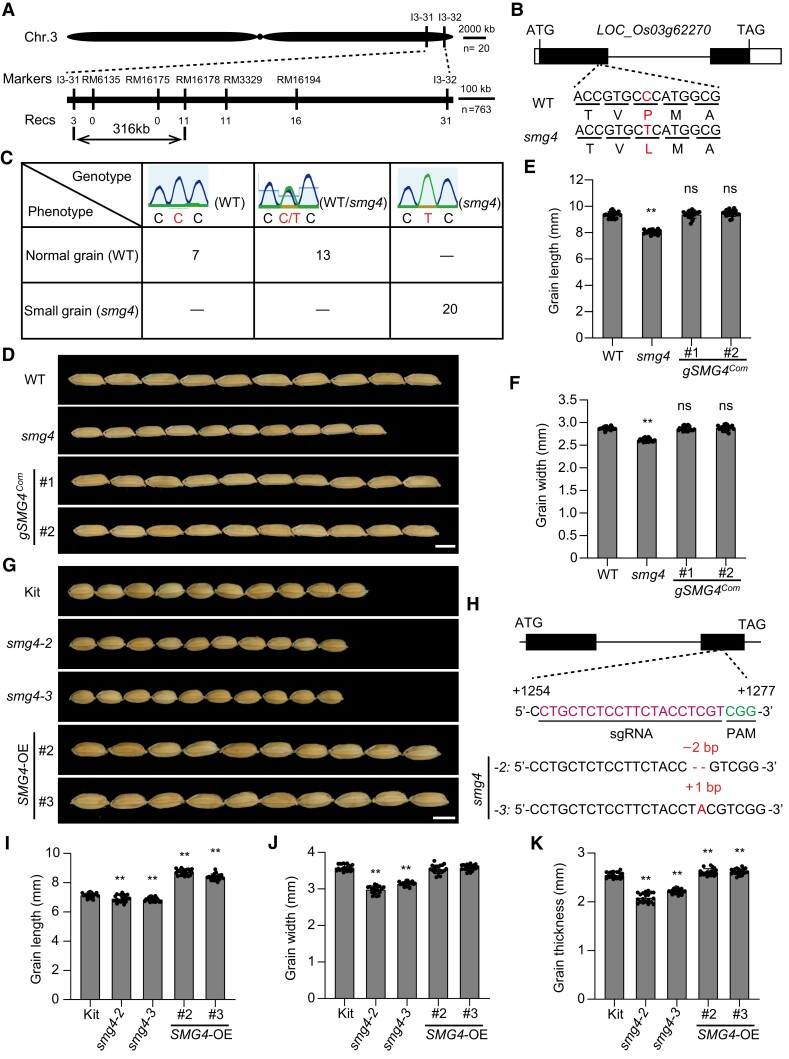
Map-based cloning of *SMG4.***A)** Fine mapping of the *SMG4* locus. The molecular markers and numbers of recombinants are indicated above and below the filled bars, respectively. Chr., chromosome; Recs, recombinants. **B)** Gene structure of *SMG4* (LOC_Os03g62270) and the mutation in *smg4*. The boxes and lines indicate exons and introns, respectively. ATG and TAG represent the start and stop codons, respectively. **C)** The genotypes and phenotypes co-segregate. **D)** Grain morphologies of WT (*SMG4*), *smg4*, and the complementation transgenic lines (*gSMG4^Com^* #1 and *gSMG4^Com^* #2). Scale bar, 5 mm. **E, F)** Grain length (*n* = 20) **(E)** and grain width (*n* = 20) **(F)** of WT, *smg4*, and the complementation transgenic lines (*gSMG4^Com^* #1 and *gSMG4^Com^* #2). **G)** Grain morphologies of Kitaake (Kit), *SMG4* knockout lines (*smg4-2* and *smg4-3*), and overexpression lines (*SMG4*-OE#2 and *SMG4*-OE#3). Scale bar, 5 mm. **H)** Identification of *SMG4* knockout lines generated by the CRISPR/Cas9 system. The sgRNA-targeted site and protospacer adjacent motif (PAM) are indicated in different colored fonts, respectively. The dashed line represents deleted nucleotides. The added base is highlighted. **I–K)** Grain length (*n* = 20) **(I)**, grain width (*n* = 20) **(J)**, and grain thickness (*n* = 20) (K) of Kit, *SMG4* knockout and overexpression transgenic lines. Values are means ± SD. Student's *t*-test was used to calculate the *P*-values, **P < 0.01. ns, no significance.

Genetic analysis revealed that the *smg4* mutant phenotype is inherited as a single nuclear recessive mutation (Supplemental Table S2). To isolate the causal gene, we generated a segregating F_2_ mapping population from a cross between *smg4* and *japonica* variety Ketan Nangka. Linkage analysis revealed that the *SMG4* locus is associated with the insertion/deletion (InDel) markers I3-31 and I3-32 on chromosome 3. Fine mapping using 763 progenies from the F_2_ population delimited the *SMG4* locus to a 316-kb genomic region between the markers I3-31 and RM16178 (Fig. 3A). Whole-genome resequencing of WT and *smg4* revealed 16 single nucleotide polymorphisms (SNPs) within the mapping region (Supplemental Table S3). Among these SNPs, only SNP2 was within an exon (Supplemental Table S3). SNP2 results in an amino acid change (Pro-315 to Leu-315) in the first exon of LOC_Os03g62270 (Fig. 3B and Supplemental Table S3), suggesting LOC_Os03g62270 as a candidate for the *SMG4* gene.

To test whether the mutation in LOC_Os03g62270 was responsible for the *smg4* mutant phenotypes, we analyzed the genotypes of 20 phenotypically WT individuals and 20 *smg4* individuals in the F_2_ population obtained by reciprocal cross between WT and *smg4*. The C→T mutation of SNP2 in LOC_Os03g62270 cosegregated with all 20 *smg4* individuals (Fig. 3C). Furthermore, we performed a complementation test by introducing a 4.7-kb WT genomic fragment of LOC_Os03g62270 into the *smg4* mutant and obtained 2 positive transgenic lines that rescued the mutant phenotypes, including grain length and grain width (Fig. 3, D–F). Moreover, we generated clustered regularly interspaced short palindromic repeat (CRISPR)/CRISPR-associated nuclease 9 (Cas9) -mediated knockouts and overexpression lines of LOC_Os03g62270 in the Kitaake (*Oryza sativa* L. ssp. *japonica*) background. Knockout lines (*smg4-2* and *smg4-3*) exhibited smaller grains with smaller grain length, width, and thickness, whereas overexpression lines (*SMG4*-OE#2 and *SMG4*-OE#3) exhibited larger grains with increased grain length, similar grain width, and increased grain thickness compared to Kitaake (Fig. 3, G–K). Similar to the *smg4* mutant, the *SMG4* knockout lines had a short stature, shorter panicles, and lower thousand-grain weight, whereas overexpression lines were taller, had longer panicles, and greater thousand-grain weight (Supplemental Fig. S3). We also transformed CRISPR/Cas9 constructs targeting the first exon of LOC_Os03g62270 into the Kitaake and Nipponbare cultivars: all 4 resulting knockout lines (*smg4-4* and *smg4-5* in Kitaake; *smg4-6* and *smg4-7* in Nipponbare) had smaller grains with lower grain length, width, and thickness (Supplemental Fig. S4). Overall, these results confirm that LOC_Os03g62270 is the *SMG4* gene.

*SMG4* encodes a MATE transporter, which is predicted to contain 12 transmembrane domains (Supplemental Fig. S5A). The amino acid affected by the *smg4* mutation is located in the eighth transmembrane domain (TM8) (Supplemental Fig. S5A). A Basic Local Alignment Search Tool search revealed that the mutated Pro-315 residue is conserved in plants (Supplemental Fig. S5B), suggesting that the site is likely important for the normal function of MATE transporters.

### Expression and topology analysis of *SMG4*

**Figure 4.**
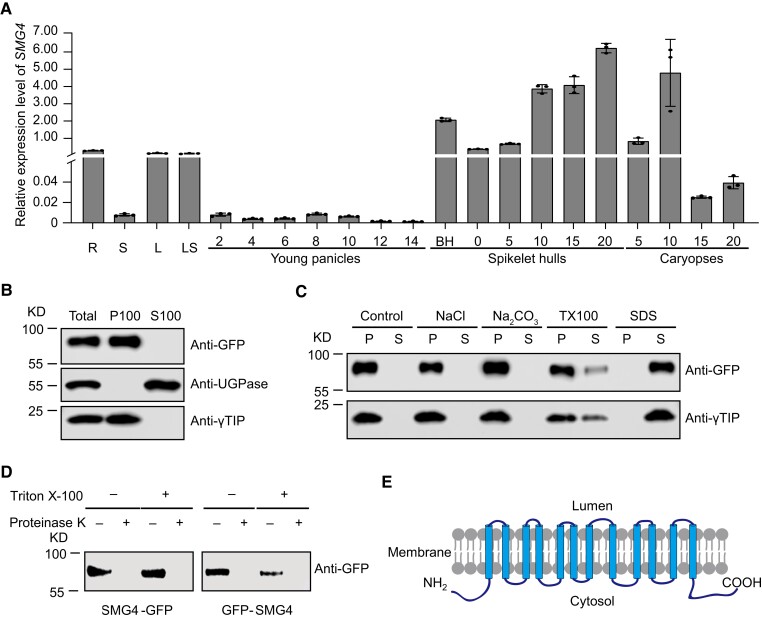
Expression of *SMG4* and topology analysis of SMG4. **A)** Relative *SMG4* expression levels in roots (R), stems (S), leaves (L), leaf sheaths (LS), young panicles (numbers indicate the length of young panicles, in cm), spikelet hulls (BH; numbers indicate the days after heading), and caryopses (numbers indicate the days after heading) of 9,311. The *UBIQUITIN* gene was used as an internal control. Values are means ± SD (*n* = 3). **B, C)** SMG4 is an integral membrane protein. Total protein extract from rice protoplasts was ultracentrifuged at 100,000 × *g* for 1 h to obtain the pellet (P100) and supernatant (S100) fraction, followed by immunoblot analysis with anti-GFP and specific antibodies for the cytosol marker anti-UGPase and the tonoplast marker anti-γTIP (tonoplast intrinsic protein) **(B)**. The P100 fraction was resuspended in various buffers as indicated. These suspensions were ultracentrifuged to obtain pellet (P) and supernatant (S), followed by immunoblot analysis with anti-GFP and anti-γTIP antibodies **(C)**. TX100, Triton X-100. **D)** Protease digestion assay. The microsomal pellets containing SMG4 tagged with GFP at either the C or N terminus were digested with or without proteinase K in the presence or absence of detergent (Triton X-100) and then analyzed by immunoblot with anti-GFP. **E)** Proposed topology of SMG4.

To clarify the function of SMG4, we analyzed the expression pattern of *SMG4* by RT-qPCR analysis. *SMG4* was widely expressed in various vegetative and reproductive organs, with higher expression in spikelet hulls and caryopsis; in addition, the expression level of *SMG4* increased continuously during spikelet hull development, with expression peaking at about 20 d after heading (Fig. 4A), which is consistent with the role of SMG4 in regulating grain size.

To analyze the membrane association of SMG4, we transfected rice protoplasts with a construct encoding SMG4-GFP (a fusion between SMG4 and the green fluorescent protein) and conducted a subcellular fractionation assay. We determined that SMG4-GFP localizes to the membrane fraction (Fig. 4B). Furthermore, SMG4 could not be extracted with high salt or alkali but could be partly solubilized with Triton X-100 and efficiently solubilized with SDS, suggesting that SMG4 is an integral membrane protein (Fig. 4C). To assess the topology of SMG4, we performed a protease digestion assay using microsomal pellets prepared from *Nicotiana benthamiana* leaves expressing *SMG4-GFP* or *GFP-SMG4*. Regardless of the presence of detergent (Triton X-100), incubation with proteinase K fully removed the N- and C-terminal GFP tags (Fig. 4D), suggesting that both termini of SMG4 are likely exposed to the cytoplasm (Fig. 4E).

### Subcellular localization of SMG4

**Figure 5.**
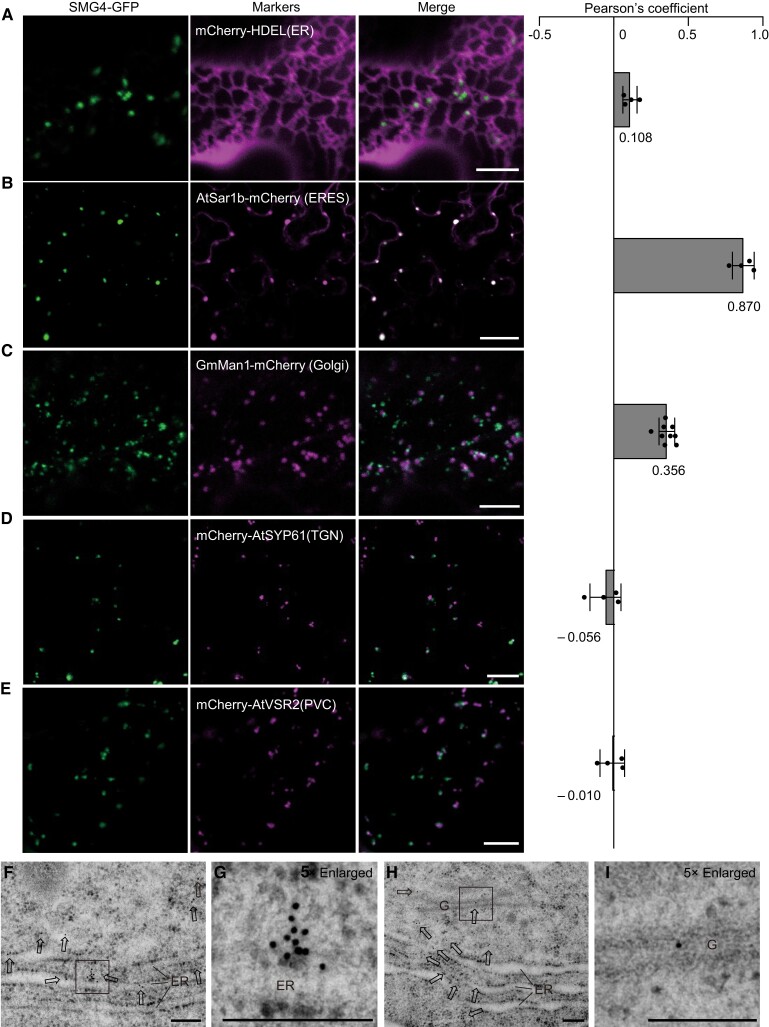
Subcellular localization of SMG4 in the leaf epidermal cells of *N*. *benthamiana* and rice root tip cells. **A to E)** Confocal microscopy images showing that SMG4-GFP localizes as puncta in the cytosol and these punctate signals partially colocalize with the marker proteins targeted to the ER (mCherry-HDEL) **(A)**, and Golgi (GmMan1-mCherry) **(C)**, strongly colocalize with ERES (AtSar1b-mCherry) **(B)**, but show a distinct localization from that of marker proteins targeted to the TGN (mCherry-SYP61) **(D)**, and PVC (mCherry-AtVSR2) **(E)**. PSC coefficients (*r_s_*) between SMG4-GFP and each marker is shown in the right panel. Values are means ± SD (*n* ≥ 4 images). Scale bars, 10 *µ*m. **F–I)** Immunoelectron microscopy localization of SMG4-GFP in root tip cells of rice. **G and I)** are the magnified images of the boxed areas in images **(F)** and **(H)**, respectively. Gold particles are highlighted with arrows. G, Golgi. ER, endoplasmic reticulum. Scale bars, 200 nm.

To investigate the subcellular localization of SMG4 in planta, we transformed an *SMG4-GFP* fusion construct into Kitaake. Examination of GFP fluorescence in the roots of the transgenic seedlings showed that the SMG4-GFP signal forms a punctate pattern in the cytosol (Supplemental Fig. S6, A and B). When expressed in *N*. *benthamiana* leaves, the SMG4-GFP signal was present in moving punctate structures (Supplemental Movie 1). To determine the nature of these punctate structures, we transiently co-expressed *SMG4-GFP* with several fluorescent marker constructs encoding fluorescent proteins targeted to the ER (mCherry-HDEL) (Nelson et al. 2007), ERESs (AtSar1b-mCherry) (Hanton et al. 2008), Golgi (GmMan1-mCherry) (Tse et al. 2004), trans-Golgi network (TGN, mCherry-SYP61) (Lam et al. 2007), and prevacuolar compartment (PVC, mCherry-VSR2) (Miao et al. 2006) in *N*. *benthamiana* leaves. Confocal microscopy observations indicated that the punctate structures of the SMG4-GFP signal overlap with the ERESs marker, with a strong correlation (*r_s_* = 0.870); we also detected a partial overlap with the ER and Golgi markers, with weaker correlations (*r_s_* = 0.108 and 0.356, respectively); and did not overlap with the TGN or PVC markers (Fig. 5, A–E). In addition, we established that a GFP fusion with the mutant SMG4 protein smg4 (SMG4^P315L^)-GFP also localizes to ERESs (Supplemental Fig. S6C). To evaluate the intracellular localization of SMG4, we performed immunogold microscopy with anti-GFP antibodies in the roots of an *SMG4-GFP* transgenic line. We detected an enrichment of gold particles near the ER membrane, with some gold particles being distributed along the Golgi (Fig. 5, F–I and Supplemental Fig. S7). Thus, SMG4 mainly localizes to ERESs and partially to the ER and Golgi.

### SMG4 interacts with COPⅡ components to regulate grain size

**Figure 6.**
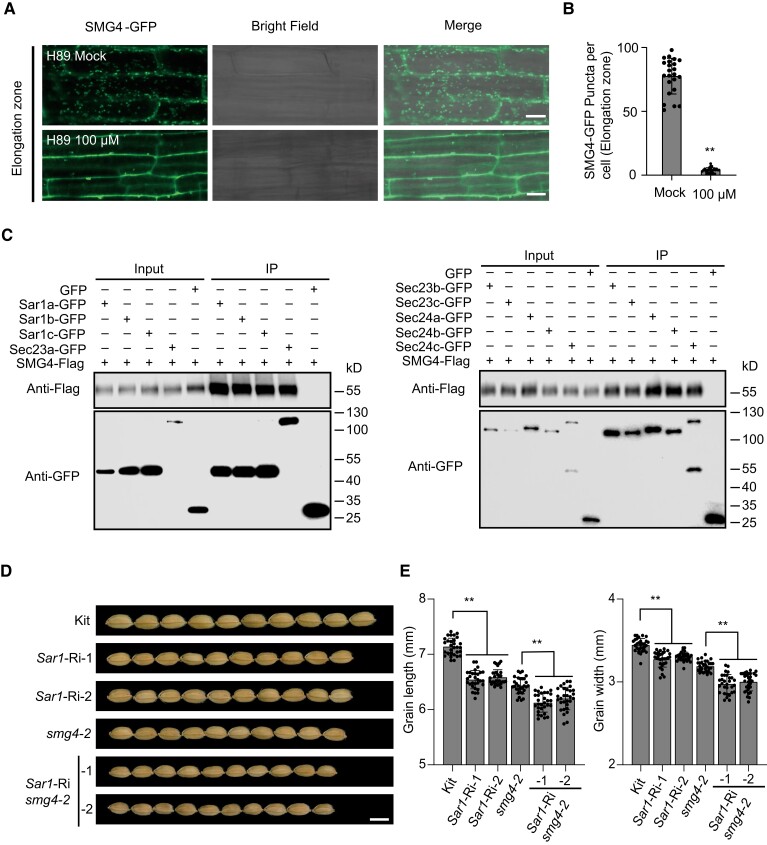
SMG4 interacts with COPⅡ components to regulate grain size. **A)** Confocal imaging of the roots from 5-d-old *SMG4-GFP* transgenic seedlings treated with or without 100 *μ*M H89 for at least 12 h. Scale bars, 10 *µ*m. **B)** Quantification of the number of SMG4-GFP puncta in (A) (*n* > 20 cells). **C)** In vivo Co-IP assay showing that SMG4 interacts with Sar1a, Sar1b, Sar1c, Sec23a, Sec23b, Sec23c, Sec24a, Sec24b, and Sec24c in rice protoplasts. The symbols “+” and “–” represent the presence and absence of the corresponding proteins. **D)** Grain morphologies of Kit, *smg4-2*, and *Sar1* (*Sar1a* + *Sar1b* + *Sar1c*) RNAi lines in the Kit and *smg4-2* backgrounds. Scale bar, 5 mm. **E)** Grain length (*n* = 30) and grain width (*n* = 30) of kit, *smg4-2*, and *Sar1* (*Sar1a* + *Sar1b* + *Sar1c*) RNAi lines in the Kit and *smg4-2* backgrounds. Values are means ± SD. Student's *t*-test was used to calculate the *P*-values, **P < 0.01.

Export from the ER is an essential cellular program driven by the COPⅡ complex, which forms vesicles at ERESs to transport secretory cargoes from the ER to the Golgi (D’Arcangelo et al. 2013; Chung et al. 2016). H89 is a serine/threonine kinase inhibitor that specifically abolishes membrane recruitment of Arabidopsis Sar1, thereby inhibiting COPⅡ assembly (Zeng et al. 2021). We treated *SMG4*-*GFP* transgenic seedlings with H89 and observed that the number of puncta labeled by SMG4-GFP significantly decreased with H89 treatment compared to the untreated control (Fig. 6, A and B). This result suggests that the localization of SMG4 likely depends on COPⅡ. To test the relation between SMG4 and COPⅡ, we performed luciferase complementation imaging (LCI) assays in *N*. *benthamiana* leaves and determined that SMG4 interacts with the COPⅡ components Sar1a, Sar1b, Sar1c, Sec23a, Sec23b, Sec23c, Sec24a, Sec24b, and Sec24c (Supplemental Fig. S8). We verified these interactions by bimolecular fluorescence complementation (BiFC) assays in *N*. *benthamiana* leaf epidermal cells (Supplemental Fig. S9) and co-immunoprecipitation (Co-IP) assays in rice protoplasts (Fig. 6C). To determine the regions of SMG4 that interact with COPⅡ components, we truncated SMG4 into 3 fragments for interaction assays: an N-terminal fragment, a TM, and a C-terminal fragment (Supplemental Fig. S10A). LCI assays showed that either the N-terminal or C-terminal region but not the TM of SMG4 is required for the interaction between SMG4 and COPⅡ components (Supplemental Fig. S10). Together, these results indicate that SMG4 interacts with COPⅡ components.

To further analyze whether SMG4 affects COPⅡ-mediated ER-to-Golgi transport, we examined the localization of the vacuolar cargoes Aleurain-mCherry (Zeng et al. 2015) and Cysteine protease (CYSP)-mCherry (Delgadillo et al. 2020) as well as the plasma membrane cargoes SECRETORY CARRIER MEMBRANE PROTEIN 1 (SCAMP1)-GFP (Cai et al. 2011) and PLASMA MEMBRANE INTRINSIC PROTEIN 2; 7 (PIP2; 7)-GFP (Hachez et al. 2014), which were transported by COPⅡ, in protoplasts of Kitaake, *smg4-2*, and *SMG4*-OE#3. A Sar1 dominant-negative mutant (Sar1DN) was previously reported to inhibit ER-to-Golgi transport in plant cells (Zeng et al. 2015). We thus cotransfected a *Sar1cDN-flag* construct with the above constructs encoding each cargo in protoplasts as control. Microscopy analysis of the transfected protoplasts showed that the vacuolar cargoes (Aleurain-mCherry and CYSP-mCherry) and the plasma membrane cargoes (SCAMP1-GFP and PIP2; 7-GFP) are delivered normally to the vacuole and plasma membrane in both *smg4-2* and *SMG4*-OE#3 protoplasts, whereas co-expressing *Sar1cDN-flag* significantly inhibited their transport (Supplemental Fig. S11), indicating that the COPⅡ-mediated ER-to-Golgi secretion route is not clearly disrupted in either *smg4-2* or *SMG4*-OE#3. These data suggest that SMG4 likely does not affect the general transport function of COPⅡ.

A previous study showed that the highly similar Sar1a, Sar1b, and Sar1c proteins are functionally redundant in rice endosperm (Tian et al. 2013). To explore the role of COPⅡ in regulating grain size, we knocked down *Sar1*, a key component of COPⅡ, using RNA interference (RNAi) in the Kitaake background. We constructed the RNAi vector using a fragment of the coding sequence that is highly similar among *Sar1a*, *Sar1b*, and *Sar1c*. We selected 2 *Sar1*-RNAi lines (*Sar1*-Ri-1 and *Sar1*-Ri-2) with significantly lower transcript levels of *Sar1s* (*Sar1a*, *Sar1b*, and *Sar1c*) for grain size measurements (Supplemental Fig. S12A). *Sar1*-RNAi lines (*Sar1*-Ri-1 and *Sar1*-Ri-2) exhibited smaller grains compared to Kitaake (Fig. 6, D and E), indicating that Sar1s play an important role in grain size regulation in rice. To analyze the genetic relationship between *SMG4* and *Sar1*s, we knocked down *Sar1*s in the *smg4-2* background with the same RNAi construct (Supplemental Fig. S12B), revealing the smaller grain size of 2 *Sar1*-Ri *smg4-2* lines compared to that of *smg4-2* (Fig. 6, D and E), suggesting that besides SMG4, other factors may also regulate grain size through COPⅡ. Therefore, these results suggest that SMG4 interacts with COPⅡ components to promote grain size.

### SMG4 acts in a common pathway with CYP78As to regulate grain size

**Figure 7.**
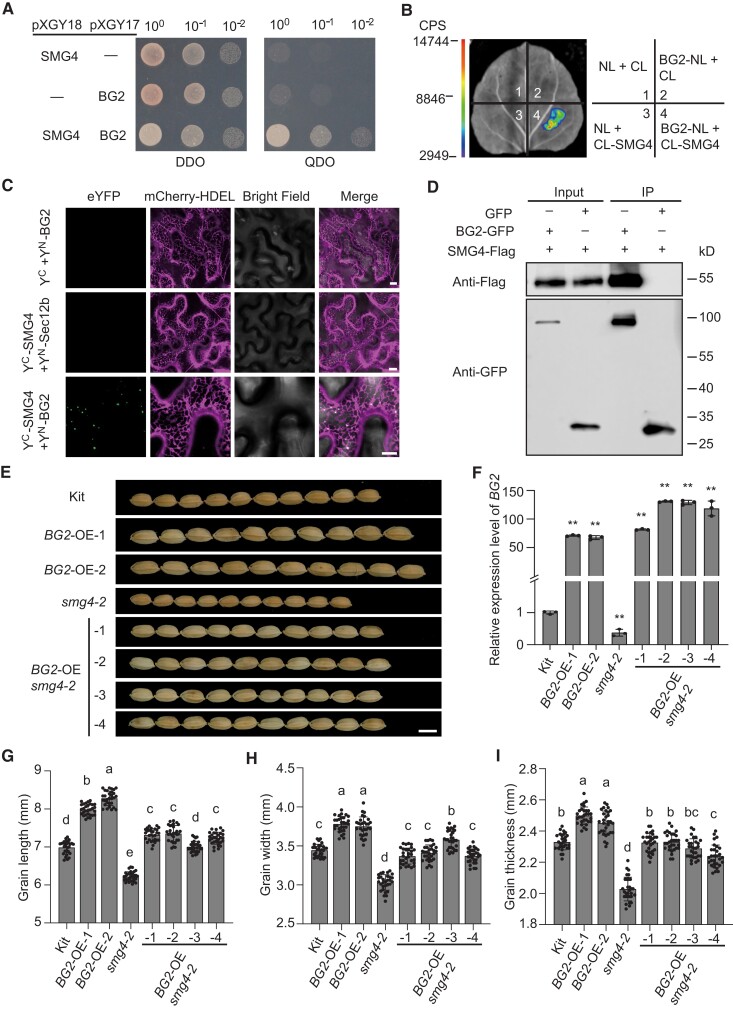
SMG4 acts genetically with BG2 to regulate grain size. **A)** Yeast 2-hybrid assay showing the interaction between SMG4 and BG2. DDO, synthetic defined medium lacking Trp and Leu (SD/–Trp–Leu); QDO, SD medium lacking Trp, Leu, His, and Ade (SD/–Trp/–Leu–His/–Ade). **B)** Firefly LCI assay showing the interaction between SMG4 and BG2 in *N*. *benthamiana* leaf cells. CL, C terminus of LUC; NL, N terminus of LUC. The scale bar indicates the luminescence intensity in counts per second (CPS). **C)** BiFC assay showing that SMG4 interacts with BG2 in *N*. *benthamiana* leaf cells. Sec12b was used as a negative control. Scale bars, 10 *µ*m. **D)** In vivo Co-IP assay showing that SMG4 interacts with BG2 in rice protoplasts. The symbols “+” and “–” represent the presence and absence of the corresponding proteins. **E)** Grain morphologies of Kit, *smg4-2*, and *BG2* overexpression lines in the Kitaake and *smg4-2* backgrounds. Scale bar, 5 mm. **F)** Relative *BG2* transcript levels in spikelet hulls of Kit, *smg4-2*, and *BG2* overexpression lines in the kit and *smg4-2* backgrounds (*n* = 3). The *UBIQUITIN* gene was used as an internal control. Values are means ± SD. Student's *t*-test was used to calculate the *P*-values, **P < 0.01. (G–I) Grain length (*n* = 30) **(G)**, grain width (*n* = 30) **(H)**, and grain thickness (*n* = 30) **(I)** of kit, *smg4-2*, and *BG2* overexpression lines in the Kit and *smg4-2* backgrounds. Values are means ± SD. Different letters indicate significant differences ranked by pairwise multiple comparison followed by Tukey's test (*P* < 0.05).

Phylogenetic analysis and multiple sequence alignment showed that SMG4 is highly similar to Arabidopsis BIGE1A and maize BIGE1, with Pro-315, which is changed to Leu-315 in the *smg4* mutant, being conserved in these proteins (Supplemental Figs. S13 and S14). In addition, *bige1a* mutants also exhibit a small grain phenotype in Arabidopsis and BIGE1 plays a role in the feedback regulation of the CYP78A pathway in maize (Suzuki et al. 2015). The CYP78A members, Arabidopsis KLU and rice BG2 were reported to promote seed (grain) size likely by generating a mobile growth signal (Adamski et al. 2009; Xu et al. 2015). *BG2*, similar to *SMG4*, was also highly expressed in spikelet hulls (Supplemental Fig. S15A) and BG2 localized to the ER in *N. benthamiana* leaf cells (Supplemental Fig. S15B). These results suggested that SMG4 and BG2 might both contribute to grain size regulation. To test this hypothesis, we performed a yeast 2-hybrid assay and discovered that SMG4 interacts with BG2 (Fig. 7A), which we confirmed by an LCI assay (Fig. 7B), a BiFC assay in *N*. *benthamiana* leaves (Fig. 7C), and a Co-IP assay in rice protoplasts (Fig. 7D). In addition, the BiFC and LCI assays showed that smg4 (SMG4^P315L^) also interacts with BG2 (Supplemental Fig. S16), revealing that the 315P→315L mutation in the smg4 mutant protein does not affect the interaction between SMG4 and BG2. Taken together, these results indicate that SMG4 physically interacts with BG2.

Previous studies have shown that a gain-of-function *BG2* mutant exhibits larger grains (Xu et al. 2015). To verify the role of *BG2* in regulating rice grain size, we generated *BG2* knockout lines in the Kitaake background using CRISPR/Cas9. However, the grain size of *BG2* knockout lines (*bg2-1* and *bg2-2*) was comparable to that of Kitaake (Supplemental Fig. S17, A–E), which may be due to functional redundancy between *BG2* and its homologs (Supplemental Fig. S17F). We also overexpressed *BG2* in the Kitaake background. The *BG2* overexpression lines (*BG2*-OE-1 and *BG2*-OE-2) produced larger grains than Kitaake, as previously reported (Fig. 7, E–I) (Xu et al. 2015), confirming that BG2 does promote grain size in rice. To determine the genetic relationship between *SMG4* and *BG2*, we overexpressed *BG2* in the *SMG4* knockout mutant *smg4-2* and observed that the smaller grain length, width, and thickness of *smg4-2* are largely, but not fully, rescued by *BG2* (Fig. 7, E–I). These observations suggest that *SMG4* acts, at least in part, in a common pathway with *BG2* to regulate grain size.

**Figure 8.**
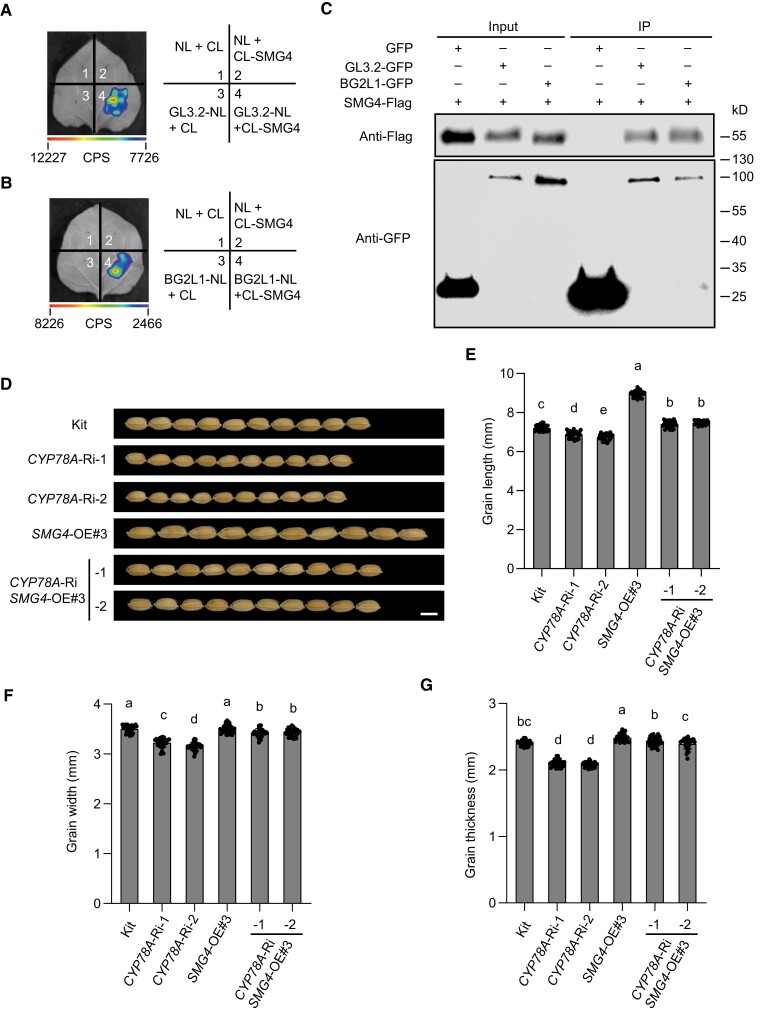
SMG4 acts in a common pathway with CYP78As to regulate grain size. **A and B)** Firefly LCI assays shows that SMG4 interacts with GL3.2 **(A)** and BG2L1 **(B)** in *N*. *benthamiana* leaves cells. CL, C terminus of LUC; NL, N terminus of LUC. The scale bar indicates the luminescence intensity in CPS. **C)** In vivo Co-IP assay showing that SMG4 interacts with GL3.2 and BG2L1 in rice protoplasts. The symbols “+” and “–” represent the presence and absence of the corresponding proteins. **D)** Grain morphologies of *CYP78A* (*BG2* + *GL3.2* + *BG2L1*) RNAi lines in the kit and *SMG4*-OE#3 backgrounds. Scale bar, 5 mm. **E–G)** Grain length (*n* = 30) **(D)**, grain width (*n* = 30) **(E)**, and grain thickness (*n* = 30) **(F)** of kit and *CYP78A* (*BG2* + *GL3.2* + *BG2L1*) RNAi lines in the Kit and *SMG4*-OE#3 backgrounds. Values are means ± SD. Different letters indicate significant differences ranked by pairwise multiple comparison followed by Tukey's test (*P* < 0.05).

To test for functional redundancy between *BG2* and its 3 homologous genes, *GL3.2*, *BG2-LIKE 1* (*BG2L1*), and *BG2L2* (Supplemental Fig. S17F), we measured the expression of *GL3.2*, *BG2L1*, and *BG2L2* in spikelet hulls of *bg2-1* and Kitaake. The expression levels of *GL3.2* and *BG2L1* were significantly increased in *bg2-1*, while *BG2L2* expression was not evidently changed (Supplemental Fig. S17G), suggesting that *BG2* and its homologs may redundantly regulate grain size. Further, LCI assays in *N*. *benthamiana* leaves and Co-IP assays in rice protoplasts indicated that SMG4 also interacts with GL3.2 and BG2L1 (Fig. 8, A–C). To verify the genetic relationship between *SMG4* and *CYP78A*s (*BG2, GL3.2*, and *BG2L1*), we selected a stretch of highly similar coding sequence among *BG2*, *GL3.2*, and *BG2L1* to construct an RNAi vector to knock down all 3 *CYP78A*s in the Kitaake and *SMG4*-OE#3 backgrounds (Supplemental Fig. S18). We selected 2 *CYP78A*-RNAi lines that exhibited smaller grains compared to Kitaake (Fig. 8, D–G); the downregulation of *CYP78A*s expression in the *CYP78A*-Ri *SMG4*-OE#3 lines partially suppressed the larger grain size of *SMG4*-OE#3 (Fig. 8, D–G), indicating that SMG4 acts, at least in part, in a common pathway with CYP78As to regulate grain size.

### The CYP78A–MATE pathway is likely conserved in monocots and dicots

**Figure 9.**
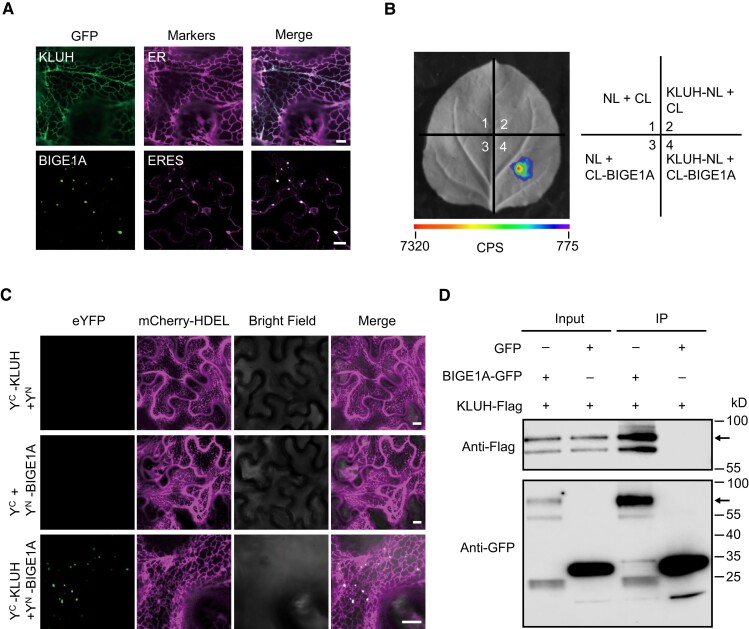
BIGE1A interacts with KLUH in Arabidopsis. **A)** Subcellular localization of the KLUH-GFP and BIGE1A-GFP fusion proteins in the leaf epidermal cells of *N*. *benthamiana*. mCherry-HDEL and AtSar1b-mCherry were used as the ER marker and ERES marker, respectively. Scale bars, 10 *µ*m. **B)** Firefly LCI assay showing that BIGE1A interacts with KLUH in *N*. *benthamiana* leaf cells. CL, C terminus of LUC; NL, N terminus of LUC. The scale bar indicates the luminescence intensity in CPS. **C)** BiFC assay showing the interaction between BIGE1A and KLUH in *N*. *benthamiana* leaf cells. mCherry-HDEL was used as the ER marker. Scale bars, 10 *µ*m. **D)** In vivo Co-IP assay showing that BIGE1A interacts with KLUH in rice protoplasts. The symbols “+” and “–” represent the presence and absence of the corresponding proteins. Arrows indicate the corresponding protein bands.

Phylogenetic analysis revealed that BIGE1A and KLUH of Arabidopsis are homologous to SMG4 and BG2 of rice, respectively (Supplemental Figs. S13 and S17F). Similar to SMG4 and BG2, BIGE1A and KLUH are members of the MATE family and CYP78A family, respectively, and *BIGE1A* and *KLUH* also regulate seed size in Arabidopsis (Adamski et al. 2009; Suzuki et al. 2015). Subcellular localization assays showed that KLUH and BIGE1A also localize to the ER and ERESs, respectively (Fig. 9A). Further, we confirmed the interaction between BIGE1A and KLUH using an LCI assay (Fig. 9B), a BiFC assay in *N*. *benthamiana* leaves (Fig. 9C), and a Co-IP assay in Arabidopsis protoplasts (Fig. 9D). Based on these observations, together with the above findings that *SMG4* genetically functions together with *CYP78A*s to regulate grain size in rice, we conclude that the CYP78A–MATE pathway regulating grain (seed) size is likely conserved in both monocots and dicots.

## Discussion

Other than phytohormones and transcriptional regulatory factors, several signaling pathways, such as the ubiquitin–proteasome pathway, the mitogen-activated protein kinase (MAPK) signaling pathway, and the G-protein signaling pathway, have been demonstrated to control grain size (Li and Li 2016). While the CYP78A pathway also plays a conserved and key role in regulating grain (seed) size in plants (Adamski et al. 2009; Chakrabarti et al. 2013; Ma et al. 2015; Suzuki et al. 2015; Wang et al. 2015b; Xu et al. 2015; Zhao et al. 2016; Qi et al. 2017), its role in regulating grain (seed) size remains largely unknown. Here, we describe a previously missed pathway, CYP78As–SMG4–COPⅡ, which promotes grain size in rice.

For secretory cargoes, efficient sorting into COPⅡ carriers depends on transmembrane receptors that physically link cargoes with coat subunits (Barlowe and Helenius 2016). In this study, we showed that SMG4 is a MATE transporter with 12 transmembrane domains (Fig. 3 and Supplemental Fig. S5A), mainly localizes to ERESs and partially localizes to the ER and Golgi (Fig. 5). Its localization pattern likely depends on COPⅡ (Fig. 6, A and B). SMG4 interacts with COPⅡ components (Fig. 6C and Supplemental Figs. S8 to S10) but does not affect COPⅡ general transport function (Supplemental Fig. S11). These characteristics are reminiscent of a canonical cargo receptor that links the ER lumenal cargo to the COPⅡ coat (Barlowe and Helenius 2016). Therefore, we propose that the transmembrane protein SMG4 might function as a COPⅡ-associated cargo receptor and transport specific cargo from the ER to the Golgi.

Previous studies have shown that the TM8, along with TM7, is important for MATE transporters to bind to and transport their cargoes (Miyauchi et al. 2017). In our study, we found that a conserved amino acid in plant SMG4 homologs was changed in TM8 of the smg4 (SMG4^P315L^) mutant protein, resulting in smaller grains in rice (Figs. 1 and 3 and Supplemental Figs. S5 and S14). Although smg4 also interacts with BG2 (Supplemental Fig. S16), the expression levels of *BG2* as well as *GL3.2* and *BG2L1* were significantly lower in the *smg4* mutants (Supplemental Fig. S19). Based on these results, we speculate that the mutation in TM8 may affect the ability of smg4 to bind to or transport cargoes, thereby resulting in the small grain phenotype.

Phytohormones such as brassinosteroids, auxin, and ABA are important for grain growth (Cheng et al. 2014; Liu et al. 2015; Liu et al. 2017). Several MATE proteins have been reported to be involved in auxin biosynthesis (ALTERED DEVELOPMENT PROGRAM 1, ADP1) and ABA transport (DETOXIFICATION EFFLUX CARRIER 50 [DTX50] and DG1) (Li et al. 2014; Zhang et al. 2014; Qin et al. 2021). To assess whether phytohormones are the cargo transported by SMG4, we analyzed the content of 8 plant hormones in the spikelet hulls of Kitaake, *smg4-2*, and *SMG4*-OE (Supplemental Fig. S20). Except for auxin, the 7 other plant hormones showed no opposite changes between *smg4-2* and *SMG4*-OE (Supplemental Fig. S20). Interestingly, auxin content increased in *smg4-2* and decreased in *SMG4*-OE (Supplemental Fig. S20). Although a low auxin concentration promotes plant growth and development, while high auxin concentration has the opposite effect, we do not think that auxin is the cargo transported by SMG4. Why? First, compared to Kitaake, the auxin concentration in *smg4-2* and *SMG4*-OE increased or decreased by less than 2-fold. Second, there were no corresponding opposite changes in auxin biosynthesis precursors, bound auxins, or oxidized auxin between *smg4-2* and *SMG4*-OE (Supplemental Fig. S21). Taken together, the cargo transported by SMG4 may not be a classical phytohormone.

To verify that CYP78As are the cargoes transported by SMG4, we tested the subcellular localization of BG2, GL3.2, and BG2L1 in protoplasts prepared from Kitaake, an *SMG4* mutant and a *Sar1*-RNAi line, showing that BG2, GL3.2 and BG2L1 all localized to the ER in both *smg4-2* and *Sar1*-Ri-1, as in Kitaake (Supplemental Fig. S22). This finding suggests that CYP78As are unlikely to be the cargoes transported by SMG4. Previous studies have shown that the CYP78A proteins, KLUH and BG2, likely increase grain size by generating a mobile growth signal (CYP78A-derived signal) (Adamski et al. 2009; Xu et al. 2015). However, the definitive characterization of this CYP78A-derived signal is lacking, despite numerous efforts over the past 20 years (Zondlo and Irish 1999; Ito and Meyerowitz 2000; Anastasiou et al. 2007; Adamski et al. 2009; Eriksson et al. 2010; Yang et al. 2013; Xu et al. 2015; Sun et al. 2017; Jiang et al. 2021; Nobusawa et al. 2021). Besides, CYP78A proteins generally localize to the ER (Supplemental Fig. S22), and it is unknown how the CYP78A-derived signal would be exported from the ER. In this study, we found that CYP78As interact with the MATE transporter SMG4 that localizes to ERESs in a COPⅡ-dependent manner (Figs. 5 to 8). Genetic analyses indicate that CYP78As, SMG4, and COPⅡ likely act in a common pathway to regulate grain size (Figs. 6 to 8). Therefore, we propose that the CYP78A-derived signal may be the cargo transported by SMG4–COPⅡ. In addition, overexpression of *BG2* largely rescued the grain size phenotype of the *SMG4* knockout mutant (Fig. 7), suggesting that besides SMG4, other proteins may transport the CYP78A-derived signal.

**Figure 10.**
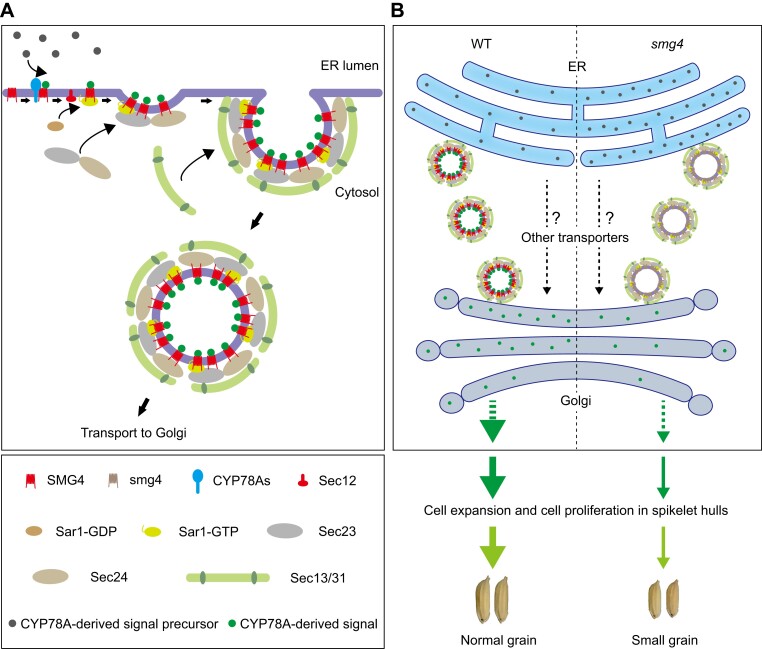
A proposed working model for SMG4's role in regulating rice grain size. **A)** CYP78As may catalyze and generate a growth signal (CYP78A-derived signal) in the ER, and SMG4 interacts with CYP78As to receive the CYP78A-derived signal. Then, SMG4 interacts with COPⅡ components to transmit the CYP78A-derived signal from the ER to Golgi. **B)** In WT, CYP78A-derived signals are transported from the ER to Golgi normally to promote cell expansion and cell proliferation in spikelet hulls, thus leading to normal grains. In *smg4*, the transport of CYP78A-derived signal from the ER to Golgi is disrupted, thus restricting cell expansion and cell proliferation in spikelet hulls and finally leading to small grains.

Finally, we propose a working model for CYP78As–SMG4–COPⅡ-mediated control of grain size in rice: CYP78As may generate a growth signal in the ER, SMG4 interacts with CYP78As to receive the CYP78A-derived signal. Then, SMG4 is loaded onto COPⅡ at ERESs, so that the CYP78A-derived signal can be transported from the ER to the Golgi by the COPⅡ-mediated vesicle transport pathway. The CYP78A-derived signal may be further transported by the Golgi to adjacent cells to regulate grain size by promoting cell expansion and cell proliferation in spikelet hulls (Fig. 10). Our findings reveal a new genetic and molecular pathway for the control of grain size, the CYP78As–SMG4–COPⅡ regulatory pathway, thus providing a new strategy for improving grain size and yield in crops.

## Materials and methods

### Plant materials and growth conditions

The *smg4* mutant was isolated from an EMS-mutagenized population of the *indica* rice (*Oryza sativa*) cultivar 9311. All plants were grown in paddy fields during the normal growing seasons at 2 field sites, Nanjing (31°93′N, 119°08′E) and Beijing (40°13′N, 116°13′E).

### Map-based cloning

To map the *smg4* locus, 783 mutant individuals were selected from a segregating F_2_ population derived from a cross between *smg4* and the *japonica* cultivar Ketan Nangka. The *smg4* locus was first mapped to a 1,200-kb region on chromosome 3 using 20 homozygous F_2_ mutant individuals by molecular markers. Next, the *smg4* locus was further mapped to a 316-kb region using 763 F_2_ mutant individuals. Furthermore, whole-genome resequencing of the WT and *smg4* was used to identify the candidate gene. Twenty phenotypically normal individuals and twenty mutant individuals selected from the backcross F_2_ population obtained by crossing 9311 and *smg4* were used for a co-segregation test. The primers used for map-based cloning are listed in Supplemental Data Set 1.

### Vector construction and plant transformation

For the complementation test, a 4.7-kb genomic DNA fragment (consisting of a 1.5-kb promoter and the entire *SMG4* coding region) was cloned into the pCUbi1390 vector (at the HindIII and BamHI sites) to generate the *proSMG4:gSMG4* construct, which was introduced into the calli of *smg4* via Agrobacterium (*Agrobacterium tumefaciens*)-mediated transformation (Hiei et al. 1994).

For knocking out *SMG4*, 20-bp gene-specific sequences targeting the first or second exon of *SMG4* were inserted into the sgRNA/Cas9 vector (at the BsaI site) to generate the *SMG4-Cas9* constructs. Then, the constructs were introduced into the calli of Kitaake and Nipponbare via Agrobacterium-mediated transformation.

For the *SMG4* overexpression construct, the full-length coding sequence of *SMG4* was amplified by PCR using cDNA prepared from total RNA of WT leaves, and the fragment was ligated to the pCUbi1390 vector (at the KpnI and BamHI sites) to generate the *SMG4* overexpression construct. Then, the construct was introduced into Kitaake calli via Agrobacterium-mediated transformation.

For the *SMG4-GFP* construct, the full-length coding sequence of *SMG4* was cloned into the pCAMBIA1305-GFP vector (at the XbaI and BamHI sites) to generate the 1305-SMG4-GFP construct. Then, the construct was introduced into Kitaake calli via Agrobacterium-mediated transformation.

For the *Sar1*-RNAi construct, a 117-bp fragment of the *Sar1b* coding sequence with high similarity to *Sar1a* and *Sar1c* was amplified by PCR to obtain Sar1-2300RNAi-KpnI and Sar1-2300RNAi-SnaBI fragments. The Sar1-2300RNAi-KpnI fragment was ligated to the FAD2-2300RNAi vector (at the KpnI site) to generate the Sar1-2300RNAi-KpnI vector. Then the Sar1-2300RNAi-SnaBI fragment was cloned into the Sar1-2300RNAi-KpnI vector (at the SnaBI site) to construct the Sar1-2300RNAi construct. Finally, the construct was introduced into the calli of Kitaake and *smg4-2* via Agrobacterium-mediated transformation.

For the *CYP78A*-RNAi construct, a 120-bp fragment of the *BG2* coding sequence with high similarity to *GL3.2* and *BG2L1* was amplified by PCR to obtain CYP78A-2300RNAi-KpnI and CYP78A-2300RNAi-SnaBI fragments. The CYP78A-2300RNAi-KpnI fragment was ligated to the FAD2-2300RNAi vector (at the KpnI site) to generate the CYP78A-2300RNAi-KpnI vector. Then the CYP78A-2300RNAi-SnaBI fragment was cloned into the CYP78A-2300RNAi-KpnI vector (at the SnaBI site) to construct the CYP78A-2300RNAi construct. Finally, the construct was introduced into the calli of Kitaake and *SMG4*-OE#3 via Agrobacterium-mediated transformation.

For knocking out *BG2*, 20-bp gene-specific sequences targeting the first exon of *BG2* were inserted into the sgRNA/Cas9 vector (at the BsaI site) to generate the *BG2-Cas9* construct. Then, the construct was introduced into the calli of Kitaake via Agrobacterium-mediated transformation.

For the *BG2* overexpression construct, the full-length coding sequence of *BG2* was cloned into the pCAMBIA2300 vector (at the SmaI site) to generate the BG2-pCAMBIA2300 overexpression construct. Then the BG2-pCAMBIA2300 construct was introduced into the calli of Kitaake and *smg4-2* via Agrobacterium-mediated transformation. All transgenic lines were analyzed using stable T2 to T3 progeny. All primers used in this assay are listed in Supplemental Data Set 1.

### RNA extraction and RT-qPCR analysis

Total RNA extraction and RT-qPCR analysis were conducted as previously described (Zhou et al. 2020). Briefly, total RNA was extracted from collected tissues using an RNAprep Pure Plant Kit (Tiangen). Reverse transcription was performed using a PrimeScript II 1st Strand cDNA Synthesis Kit (TaKaRa), and qPCR was performed using a SYBR Premix Ex Taq Kit (TaKaRa) and the ABI prism 7500 Real-Time PCR System.

### Subcellular fractionation

The full-length coding sequence of *SMG4* was amplified and inserted into the pAN580 vector (at the XbaI and BamHI sites), generating the fusion construct pAN580-SMG4-GFP. The construct was transfected into rice protoplasts as described previously (Zhang et al. 2011). Subcellular fractionation was performed as described previously with some modifications (Ren et al. 2020). Briefly, protoplasts, incubated overnight at 25 °C, were collected with ice-cooled buffer A (100 mm HEPES-KOH, pH 7.5, 0.3 m sucrose, 5 mm EGTA, 5 mm MgCl_2_, and 1× protease inhibitor [complete cocktail tablets; Roche]) and incubated on ice for 10 min. The homogenate was centrifuged at 10,000 × *g* for 10 min at 4 °C to remove large cellular debris. The supernatant was further centrifuged at 100,000 × *g* for 1 h at 4 °C; the supernatant and pellet were assigned as S100 and P100 fractions for immunoblot analysis, respectively. The P100 fraction was further resuspended in 150 mL each of buffer A, high salt buffer (buffer A supplemented with 1 m NaCl), alkaline buffer (buffer A supplemented with 0.1 m Na_2_CO_3_, pH 11), Triton X-100 buffer (buffer A supplemented with 1% [v/v] Triton X-100), and SDS buffer (buffer A supplemented with 1% [w/v] SDS). After incubation for 20 min on ice, these resuspension solutions were centrifuged at 100,000 × *g* for 1 h at 4 °C to obtain the supernatant (S) and pellet (P) fractions for immunoblot analysis. Primers used in this assay are listed in Supplemental Data Set 1.

### Topology analysis

Topology analysis was performed as previously described (Wang et al. 2016b). Briefly, the full-length coding sequence of *SMG4* was amplified and inserted in-frame and upstream or downstream of the *GFP* sequence in the vector pCAMBIA1305-GFP to generate the 1305-SMG4-GFP and 1305-GFP-SMG4 constructs, respectively. These constructs were introduced into the Agrobacterium strain EHA105 and then used to infiltrate *N*. *benthamiana* leaves. Infiltrated *N*. *benthamiana* leaves (∼3 g) transiently accumulating SMG4 fusion proteins were homogenized in 10 mL of buffer A (100 mm HEPES-KOH, pH 7.5, 0.3 m sucrose, 5 mm EGTA, 5 mm MgCl_2_, and 1× protease inhibitor [complete cocktail tablets; Roche]). The homogenates were filtered and centrifuged at 2,000 × *g* for 20 min at 4 °C to remove the cell debris. The supernatant was ultracentrifuged at 100,000 × *g* for 1 h at 4 °C to obtain the microsomal pellets. The microsomal pellets containing SMG4 protein tagged with GFP at either the C or N terminus were digested with or without 10 ng/mL proteinase K (Roche) in the presence or absence of 1% (v/v) Triton X-100 and then analyzed by immunoblotting. Immunoblots were analyzed with anti-GFP antibody (11814460001, Roche, 1:5000), and antimouse secondary antibody (D330, MBL, 1:2000). The primers used in this assay are listed in Supplemental Data Set 1.

### Subcellular localization assay

For subcellular localization of SMG4 in *N*. *benthamiana* leaves, the full-length coding sequence of *SMG4* was cloned into the pCAMBIA1305-GFP vector (at the XbaI and BamHI sites) to generate the 1305-SMG4-GFP construct. The well-established fluorescent protein markers used were mCherry-HDEL for the ER (Nelson et al. 2007), AtSar1b-mCherry for ERESs (Hanton et al. 2008), GmMan1-mCherry for the Golgi (Tse et al. 2004), mCherry-SYP61 for the TGN (Lam et al. 2007), and mCherry-VSR2 for the PVC (Miao et al. 2006). These constructs and markers were introduced into Agrobacterium strain EHA105 and co-infiltrated into *N*. *benthamiana* leaves for subcellular localization analysis. Images were captured using a laser scanning confocal microscope (ZEISS LSM 980). At least 4 independent images were analyzed with the Pearson–Spearman correlation (PSC) plug-in for ImageJ to quantify the colocalization of SMG4-GFP and each marker.

For subcellular localization of smg4, BG2, KLUH, and BIGE1A in *N*. *benthamiana* leaves, the full-length coding sequences of *smg4*, *BG2*, *KLUH*, and *BIGE1A* were cloned into the pCAMBIA1305-GFP vector (at the XbaI and BamHI sites) to generate the 1305-smg4-GFP, 1305-BG2-GFP, 1305-KLUH-GFP, and 1305-BIGE1A-GFP constructs. These constructs were introduced into Agrobacterium strain EHA105 and co-infiltrated into *N*. *benthamiana* leaves with their respective markers for subcellular localization analysis. Images were captured using a laser scanning confocal microscope (ZEISS LSM 980).

For subcellular localization of SMG4 in rice roots, the 1305-SMG4-GFP vector was introduced into Kitaake calli via Agrobacterium-mediated transformation. The T2-positive transgenic seedlings were selected and their roots were used for subcellular localization observation. Images were captured using a confocal microscope (ZEISS LSM 980).

For subcellular localization of BG2, GL3.2, and BG2L1 in rice protoplasts, the full-length coding sequences of *BG*2, GL3.2, and BG2L1 were amplified and inserted into the pAN580 vector (at the XbaI and BamHI sites), generating the fusion constructs pAN580-BG2-GFP, pAN580-GL3.2-GFP, pAN580-BG2L1-GFP. The constructs were transfected into protoplasts of Kitaake, *smg4-2*, and *Sar1*-Ri-1. GFP fluorescence was observed using a confocal microscope (ZEISS LSM 980). Primers used in these assays are listed in Supplemental Data Set 1.

### Immunogold electronic microscopy

The root tips of 1305-SMG4-GFP transgenic seedlings were high-pressure frozen/freeze substituted, and ultrathin sectioning and immunogold labeling were subsequently performed as previously described by Ren et al. (2020). Finally, the samples were examined using an H7700 transmission electron microscope (Hitachi).

### Phylogenetic analysis

SMG4 homologs were identified in rice, Arabidopsis, and maize, and BG2 homologs were identified in rice and Arabidopsis using the BLASTP search program of the National Center for Biotechnology Information (https://www.ncbi.nlm.nih.gov). The phylogenetic trees were generated using MEGA 7.0, based on the neighbor-joining method with the following parameters: p-distance model, pairwise deletion, and bootstrap (1,000 replicates). The alignments are provided in Supplemental Files 1 and 3. The Newick formats of the phylogenetic trees are provided in Supplemental Files 2 and 4.

### Yeast 2-hybrid assay

A yeast 2-hybrid assay was used to detect protein interactions using the DUAL hunter system (Dualsystems Biotech). The full-length coding sequence of *BG2* was cloned in-frame with the sequence encoding the Cub fragment in the pXGY17 vector, and the full-length coding sequence of *SMG4* was cloned in-frame with the sequence encoding the Nub fragment in the pXGY18 vector. The Y2H assay was conducted according to a previously described method (Xu et al. 2017). Primers used in this assay are listed in Supplemental Data Set 1.

### Luciferase complementation imaging assay

The full-length coding sequences of *SMG4*, *smg4*, *Sar1a*, *Sar1b*, *Sar1c*, *Sec23a*, *Sec23b*, *Sec23c*, *Sec24a*, *Sec24b*, *Sec24c*, and *BIGE1A* were amplified and cloned in-frame with the sequence encoding C-terminal half of firefly luciferase (cLUC, CL) to form the *CL-SMG4*, *CL-smg4*, *CL-Sar1a*, *CL-Sar1b*, *CL-Sar1c*, *CL-Sec23a*, *CL-Sec23b*, *CL-Sec23c*, *CL-Sec24a*, *CL-Sec24b*, *CL-Sec24c*, and *CL-BIGE1A* constructs, respectively. The full-length coding sequences of *Sar1a*, *Sar1b*, *Sar1c*, *Sec23a*, *Sec23b*, *SMG4*, *BG2*, *GL3.2*, *BG2L1*, and *KLUH*, and the sequences encoding the N-terminal fragment (amino acids [aa] 1–40), transmembrane fragment (aa 41–465), and C-terminal fragment (aa 466–516) of SMG4 were amplified and cloned in-frame with the sequence encoding the N-terminal half of luciferase (nLUC, NL) to form the *Sar1a-NL*, *Sar1b-NL*, *Sar1c-NL*, *Sec23a-NL*, *Sec23b-NL*, *SMG4-NL*, *BG2-NL*, *GL3.2-NL*, *BG2L1-NL*, *KLUH-NL*, *N-NL*, *TM-NL*, and *C-NL* constructs, respectively. These constructs were introduced into Agrobacterium strain EHA105, and various combinations of EHA105 strains were co-infiltrated into *N*. *benthamiana* leaves as previously described (Cai et al. 2021). After 36 to 48 h, leaves were harvested and incubated with 1 mm luciferin (E1601, Promega) for 3 min, and luciferase activities were measured using an imaging apparatus (NightShade LB 985, Berthold). Primers used for this assay are listed in Supplemental Data Set 1.

### Bimolecular fluorescence complementation assays

The full-length coding sequences of *SMG4*, *smg4*, and *KLUH* were ligated into the C-terminal fragment of yellow fluorescent protein (YFP) in p2YC vector to generate the *Y^C^-SMG4*, *Y^C^-smg4*, and *Y^C^-KLUH* constructs, and the full-length coding sequences of *Sar1a*, *Sar1b*, *Sar1c*, *Sec23a*, *Sec23b*, *Sec23c*, *Sec24a*, *Sec24b*, *Sec24c*, *Sec12b*, *BG2*, and *BIGE1A* were ligated into the N-terminal fragment of YFP in p2YN vector to generate the *Y^N^-Sar1a*, *Y^N^-Sar1b*, *Y^N^-Sar1c*, *Y^N^-Sec23a*, *Y^N^-Sec23b*, *Y^N^-Sec23c*, *Y^N^-Sec24a*, *Y^N^-Sec24b*, *Y^N^-Sec24c*, *Y^N^-Sec12b*, *Y^N^-BG2*, and *Y^N^-BIGE1A* constructs, respectively. These constructs were introduced into Agrobacterium strain EHA105, and various combinations of EHA105 strains were co-infiltrated into *N*. *benthamiana* leaves as previously described (Waadt and Kudla 2008). After 36 to 48 h, leaves were harvested for fluorescence signal capture using a laser scanning confocal microscope (ZEISS LSM 980). Primers used for this assay are listed in Supplemental Data Set 1.

### Co-IP assay

The full-length coding sequences of *Sar1a*, *Sar1b*, *Sar1c*, *Sec23a*, *Sec23b*, *Sec23c*, *Sec24a*, *Sec24b*, *Sec24c*, *BG2*, *GL3.2*, *BG2L1*, and *BIGE1A* were ligated into the pAN580 vector to produce the pAN580-Sar1a-GFP, pAN580-Sar1b-GFP, pAN580-Sar1c-GFP, pAN580-Sec23a-GFP, pAN580-Sec23b-GFP, pAN580-Sec23c-GFP, pAN580-Sec24a-GFP, pAN580-Sec24b-GFP, pAN580-Sec24c-GFP, pAN580-BG2-GFP, pAN580-GL3.2-GFP, pAN580-BG2L1-GFP, and pAN580-BIGE1A-GFP constructs, respectively, and the full-length coding sequences of *SMG4* and *KLUH* were inserted into the pCUbi1390 vector to produce the *SMG4-Flag* and *KLUH-Flag* constructs, respectively. The construct combinations were cotransfected into rice or Arabidopsis protoplasts and incubated overnight at 25 °C. Total protein was extracted with protein extraction buffer (50 mM Tris–HCl, pH 8.0, 1 mM MgCl_2_, 10 mM EDTA, 0.5 M sucrose, 5 mM DTT, and 1× protease inhibitor [complete cocktail tablets; Roche]). The Co-IP assay was conducted as previously described (Lan et al. 2020). Immunoblots were analyzed with anti-FLAG antibody (M185-7, MBL, 1:5000), anti-GFP antibody (11814460001, Roche, 1:5000), and antimouse secondary antibody (D330, MBL, 1:2000). Primers used for this assay are listed in Supplemental Data Set 1.

### Detection of phytohormones

The spikelet hulls of Kitaake, *smg4-2*, and *SMG4*-OE#3 plants before heading (BH) (Panicles length is about 15 cm) were frozen in liquid nitrogen and ground to a dry powder. The phytohormones (5DS [5-deoxystrigol], ABA, SA [salicylic acid], tZ [trans-zeatin], ACC [1-aminocyclopropanecarboxylic acid], GA4 [gibberellin A4], IAA [indole-3-acetic acid], and JA [jasmonic acid]) and auxin biosynthesis precursors, bound auxins, and oxidized auxins (IPA [3-indolepropionic acid], TRA [tryptamine], IAA-Asp [indole-3-acetyl-L-aspartic acid], IAA-Glu [indole-3-acetyl glutamic acid], IAA-Val-Me [indole-3-acetyl-L-valine methyl ester], and OXIAA [2-oxindole-3-acetic acid]) were quantified by MetWare (http://www.metware.cn/, Wuhan, China) using the AB Sciex QTRAP4500 liquid chromatograph-MS/MS platform. Analysis was performed on 3 biological replicates.

### Statistical analysis

Microsoft Excel and GraphPad Prism 9.3 were used for data analysis. Student's *t-*tests were used for significant difference analysis between 2 samples. One-way analysis of variance (ANOVA) followed by Tukey's tests was used for pairwise multiple comparisons. *P*-value <0.05 was considered statistically significant. The statistical analyses were performed using SPSS software. All statistical results are shown in Supplemental Data Set 2.

### Accession numbers

Sequence data from this article can be found in the GenBank/EMBL libraries under the following accession numbers: *SMG4* (LOC_Os03g62270), *Sar1a* (LOC_Os01g23620), *Sar1b* (LOC_Os12g37360), *Sar1c* (LOC_Os01g15010), *Sec23a* (LOC_Os01g21850), *Sec23b* (LOC_Os08g36994), *Sec23c* (LOC_Os11g24560), *Sec24a* (LOC_Os11g29200), *Sec24b* (LOC_Os05g37120), *Sec24c* (LOC_Os04g04020), *Sec12b* (LOC_Os11g39650), *BG2* (LOC_Os07g41240), *GL3.2* (LOC_Os03g30420), *BG2L1* (LOC_Os03g40600), *EXPA5* (LOC_Os02g51040), *EXPA13* (LOC_Os02g16730), *EXPA19* (LOC_Os03g06050), *EXPA24* (LOC_Os02g16800), *EXLA4* (LOC_Os06g50960), *EXPB3* (LOC_Os10g40720), *EXPB6* (LOC_Os10g40700), *EXPB7* (LOC_Os03g01270), *EXPB14* (LOC_Os02g44106), *CycA1; 4* (LOC_Os05g14730), *CycA3; 1* (LOC_Os03g41100), *CycD3; 1* (LOC_Os06g11410), *CycD4; 1* (LOC_Os09g29100), *CycD5; 2* (LOC_Os12g39830), *CycH1; 1* (LOC_Os03g52750), *CycL1; 1* (LOC_Os01g27940), *CycT1; 3* (LOC_Os11g05850), *CycF3; 2* (LOC_Os03g11040), *UBIQUITIN* (LOC_Os03g13170), *KLUH* (AT1G13710), and *BIGE1A* (AT1G71870).

## Supplementary Material

koad239_Supplementary_Data

## Supplemental data

The following materials are available in the online version of this article.

**Supplemental Figure S1.** Time-course analysis of brown grain dry weight during grain development of WT and the *smg4* mutant.

**Supplemental Figure S2.** *SMG4* affects cell expansion in epidermal cells of the glumes.

**Supplemental Figure S3.** Phenotypes of *SMG4* knockout and overexpression transgenic plants.

**Supplemental Figure S4.** Knock out of *SMG4* decreases grain size in both Kitaake and Nipponbare.

**Supplemental Figure S5.** Structure analysis of SMG4 protein.

**Supplemental Figure S6.** Subcellular localization of SMG4 protein and smg4 mutant protein.

**Supplemental Figure S7.** Immunoelectron microscopy localization of SMG4-GFP.

**Supplemental Figure S8.** LCI assays show that SMG4 interacts with the COPⅡ components.

**Supplemental Figure S9.** BiFC assays show that SMG4 interacts with the COPⅡ components.

**Supplemental Figure S10.** The N- and C-terminal domains but not the TM of SMG4 interact with COPⅡ components.

**Supplemental Figure S11.** SMG4 likely does not affect the transport function of COPⅡ.

**Supplemental Figure S12.** Transcript levels of *Sar1s* in spikelet hulls of *Sar1* RNAi lines in Kitaake and *smg4-2* background.

**Supplemental Figure S13.** Phylogenic analysis of SMG4 and its homologs.

**Supplemental Figure S14.** Sequence alignment of SMG4 and its 6 Arabidopsis homologs.

**Supplemental Figure S15.** Expression pattern and subcellular localization of *BG2* gene.

**Supplemental Figure S16.** smg4 interacts with BG2.

**Supplemental Figure S17.** BG2 likely functions redundantly with its homologs to regulate grain size.

**Supplemental Figure S18.** Transcript levels of *CYP78As* in spikelet hulls of *CYP78A* RNAi lines in Kitaake and *SMG4*-OE#3 background.

**Supplemental Figure S19.** The transcript levels of *CYP78As* are lower in the *smg4* mutant.

**Supplemental Figure S20.** Contents of 8 plant hormones in spikelet hulls of Kitaake, *smg4-2*, and *SMG4*-OE.

**Supplemental Figure S21.** Contents of auxin biosynthesis precursors, bound auxins, and oxidized auxins in spikelet hulls of Kitaake, *smg4-2*, and *SMG4*-OE.

**Supplemental Figure S22.** Subcellular localization of BG2, GL3.2, and BG2L2 in protoplasts of Kitaake, *smg4-2*, and *Sar1*-Ri-1.

**Supplemental Table S1.** Important agronomic traits between WT and the *smg4* mutant.

**Supplemental Table S2.** Phenotypic segregation in reciprocal crosses between WT and the *smg4* mutant.

**Supplemental Table S3.** Identification of 16 SNPs between WT and *smg4* mutant within the fine mapping region.

**Supplemental Data Set 1.** List of primers used in this study.

**Supplemental Data Set 2.** Summary of statistical tests.

**Supplemental File 1.** Multiple sequence alignment for Supplemental Fig. S13.

**Supplemental File 2.** Newick format of the phylogenetic tree for Supplemental Fig. S13.

**Supplemental File 3.** Multiple sequence alignment for Supplemental Fig. S17F.

**Supplemental File 4.** Newick format of the phylogenetic tree for Supplemental Fig. S17F.

**Supplemental Movie S1.** Time-lapse microscopy of SMG4-GFP in the epidermal cells of *N. benthamiana* leaves.

## Dive Curated Terms

The following phenotypic, genotypic, and functional terms are of significance to the work described in this paper:

SAR1 Gramene: AT1G33410
SAR1 Araport: AT1G33410

grain size Planteome: TO:0000397
