# Supplementary material for: A CYP78As–small grain4–coat protein complex Ⅱ pathway promotes grain size in rice
Source: Plant Cell. 2023 Sep 21;35(12):4325–46. doi: 10.1093/plcell/koad239 (PMC10689148; doi:10.1093/plcell/koad239)
Supplement: koad239_Supplementary_Data [file koad239_supplementary_data.zip › TPC2022RA00268D_Supplemental_Data.pdf]

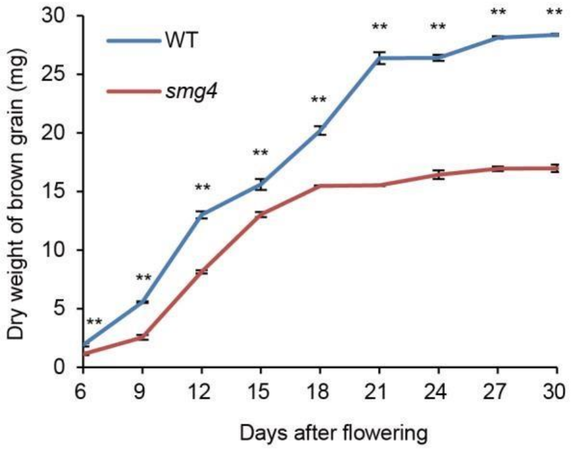

Supplemental Figure S1. **Time-course analysis of brown grain dry weight during grain development of WT and the *smg4* mutant** (Supports Figure 1)

Time-course analysis of brown grain dry weight showing that the *smg4* mutant has a lower filling rate compared to wild type (WT). Values are means ± SD (*n* = 6 plants, 15 grains per plant). Student's *t*-test was used to calculate the *P*-values, ***P* <0.01.

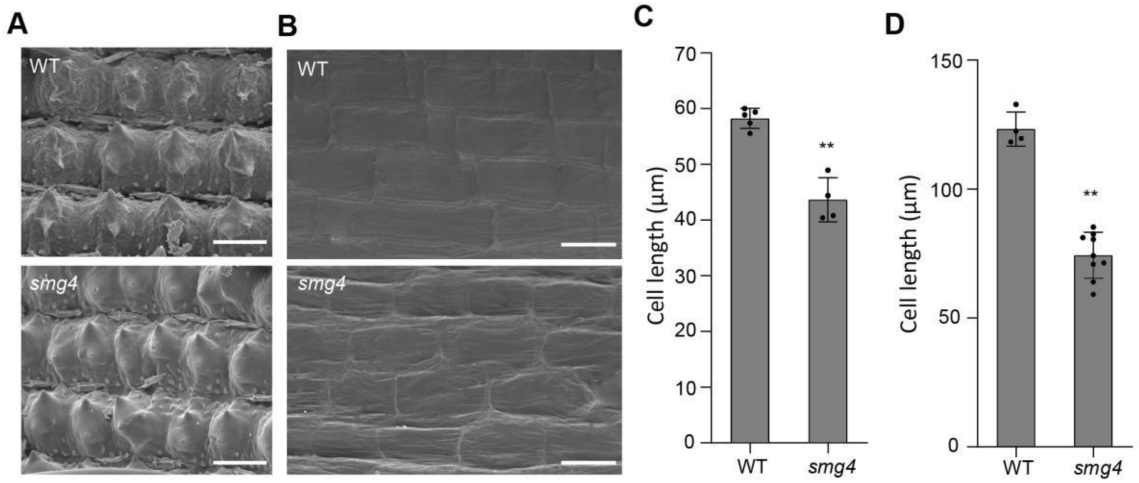

Supplemental Figure S2. *SMG4* affects cell expansion on epidermal cells of the glumes (Supports Figure 2)

(A, B) Scanning electron micrographs of the outer (**A**) and inner (**B**) glume surface of mature grains from WT and *smg4* mutant. Scale bars, 50 μm.

(C, D) Cell length on the outer (**C**) and inner (**D**) glume surface of mature grains from WT and *smg4* mutant ($n \geq 4$ images).

Values are means ± SD. Student's *t*-test was used to calculate the *P* values, $^{**}P < 0.01$.

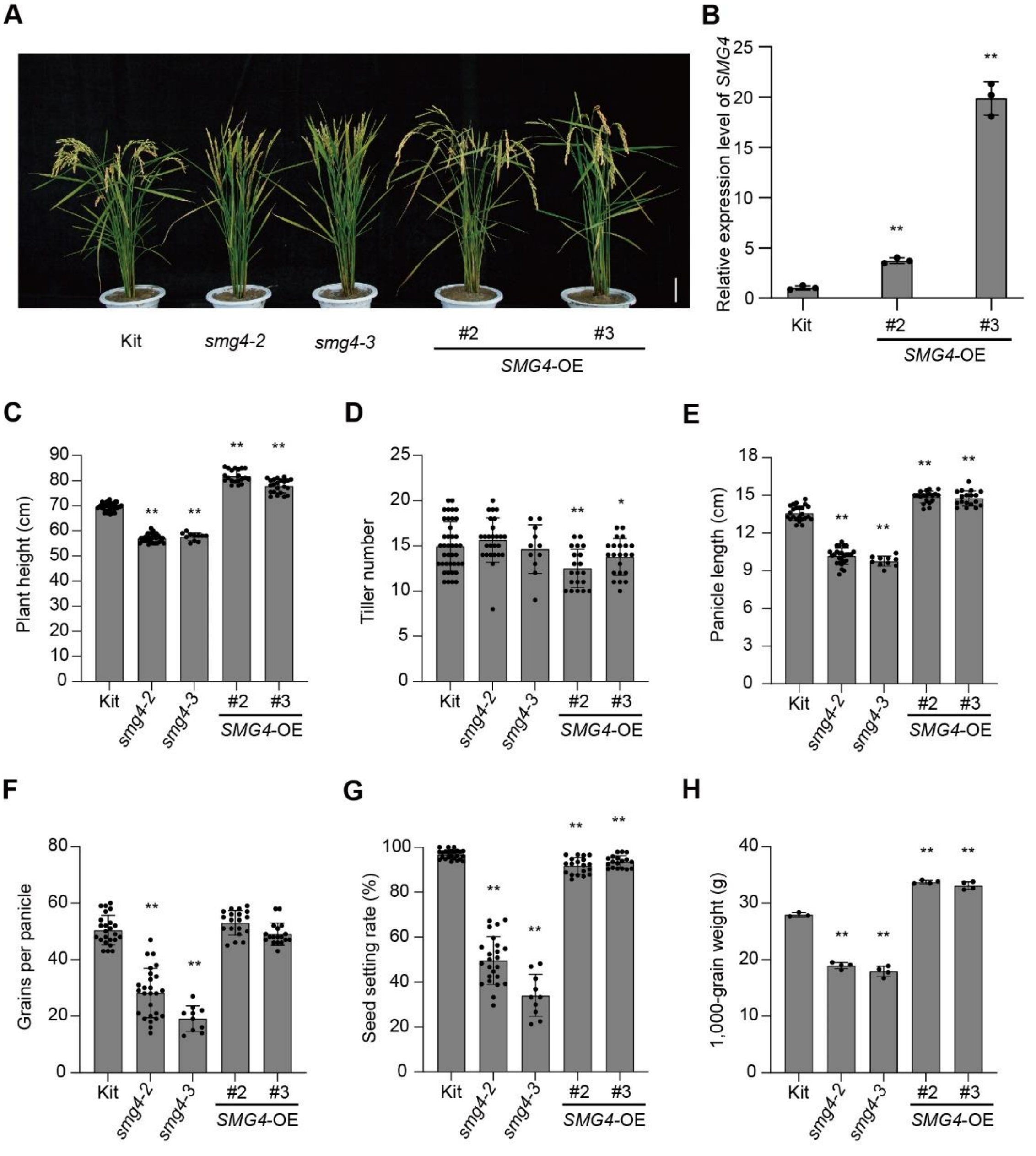

Supplemental Figure S3. Phenotypes of *SMG4* knockout and overexpression transgenic plants (Supports Figure 3)

(A) Phenotypes of Kitaake (Kit), *SMG4* knockout and overexpression transgenic plants at the mature stage. Scale bar, 10 cm.

(B) Relative *SMG4* transcript levels in spikelet hulls of Kit and *SMG4* overexpression lines (*n* = 3). The *UBIQUITIN* gene was used as an internal control.

(C–H) Plant height (*n* > 10) **(C)**, tiller number (*n* > 10) **(D)**, panicle length (*n* ≥ 10) **(E)**, grains per panicle (*n* ≥ 10) **(F)**, seed setting rate (*n* ≥ 10) **(G)**, and thousand-grain weight (*n* ≥ 3) **(H)** of Kit, *SMG4* knockout and overexpression transgenic plants.

Values are means ± SD. Student's *t*-test was used to calculate the *P* values, ***P* <0.01, **P* <0.05.

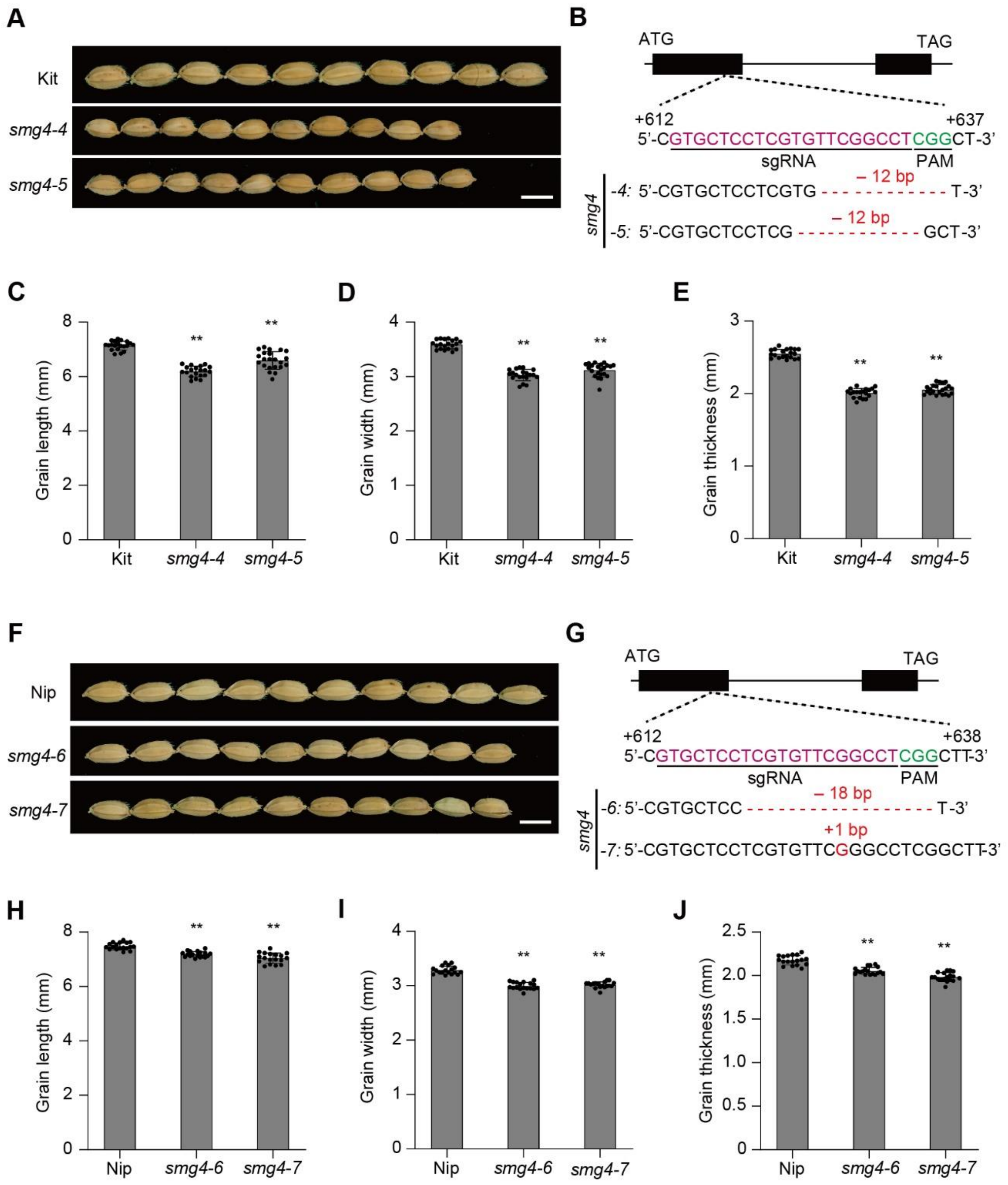

Supplemental Figure S4. Knock out of *SMG4* decreases grain size in both Kitaake and Nipponbare (Supports Figure 3)

(A) Grain morphologies of *SMG4* knockout lines (*smg4-4* and *smg4-5*) in Kitaake (Kit). Scale bar, 5 mm.

(B) Identification of knockout lines in Kit generated by the CRISPR/Cas9 technology. The sgRNA-targeted site and PAM are indicated in magenta and green font, respectively. Red dashed line represents deleted nucleotides.

(C–E) Grain length ($n \geq 20$) **(C)**, grain width ($n \geq 20$) **(D)**, and grain thickness ($n \geq 20$) **(E)** of Kit and *SMG4* knockout lines (*smg4-4* and *smg4-5*).

(F) Grain morphologies of *SMG4* knockout lines (*smg4-6* and *smg4-7*) in Nipponbare (Nip). Scale bar, 5 mm.
(G) Identification of knockout lines in Nip generated by the CRISPR/Cas9 technology. The sgRNA-targeted site and PAM are indicated in magenta and green font, respectively. Red dashed line represents deleted nucleotides. Added base is highlighted in red font.
(H–J) Grain length (*n* = 18) **(H)**, grain width (*n* = 18) **(I)**, and grain thickness (*n* = 18) **(J)** of Nip and *SMG4* knockout lines (*smg4-6* and *smg4-7*).
Values are means ± SD. Student's *t*-test was used to calculate the *P* values, $^{**}P < 0.01$.

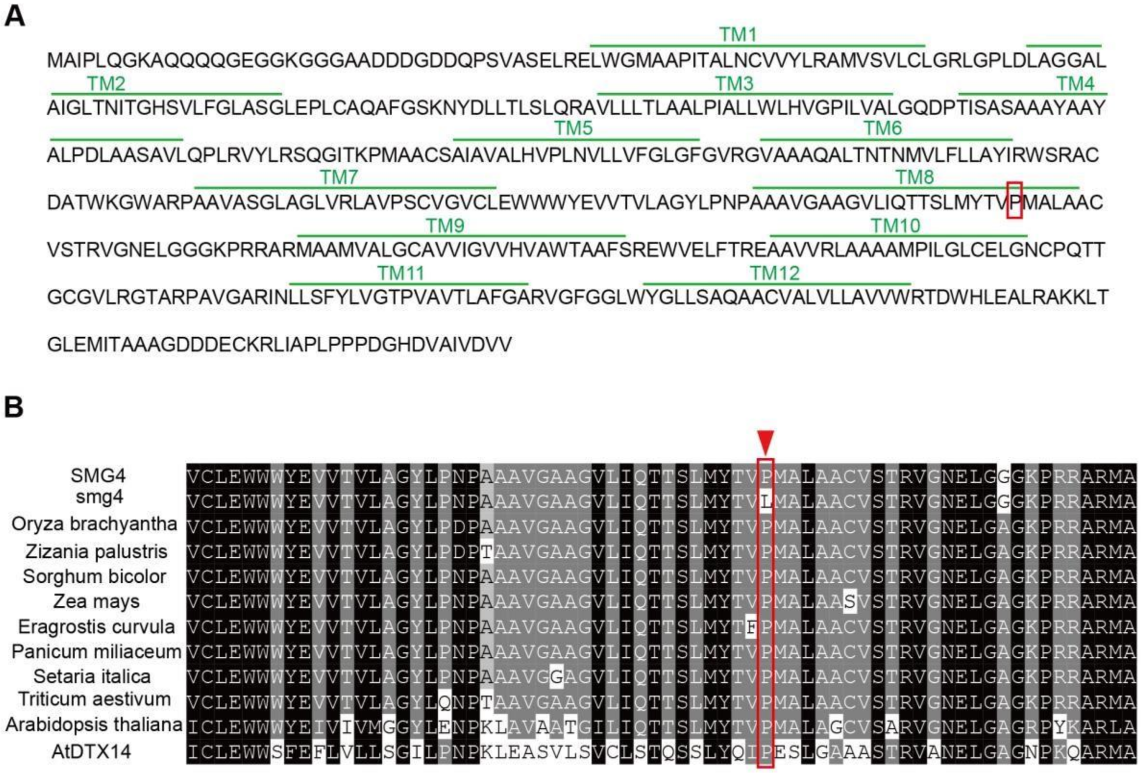

Supplemental Figure S5. Structure analysis of SMG4 protein (Supports Figure 4)

(A) Predicted transmembrane domains of SMG4. Green lines indicate the twelve transmembrane domains predicted by HMMTOP Server v. 2.0 (http://www.enzim.hu/hmmtop/html/ submit.html). Mutated amino acid in *smg4* mutant is highlighted with red box.

(B) The mutated amino acid P315 in smg4 ($SMG4^{P315L}$) is conserved in plants. The mutated amino acid in *smg4* mutant is highlighted with red triangle and red box.

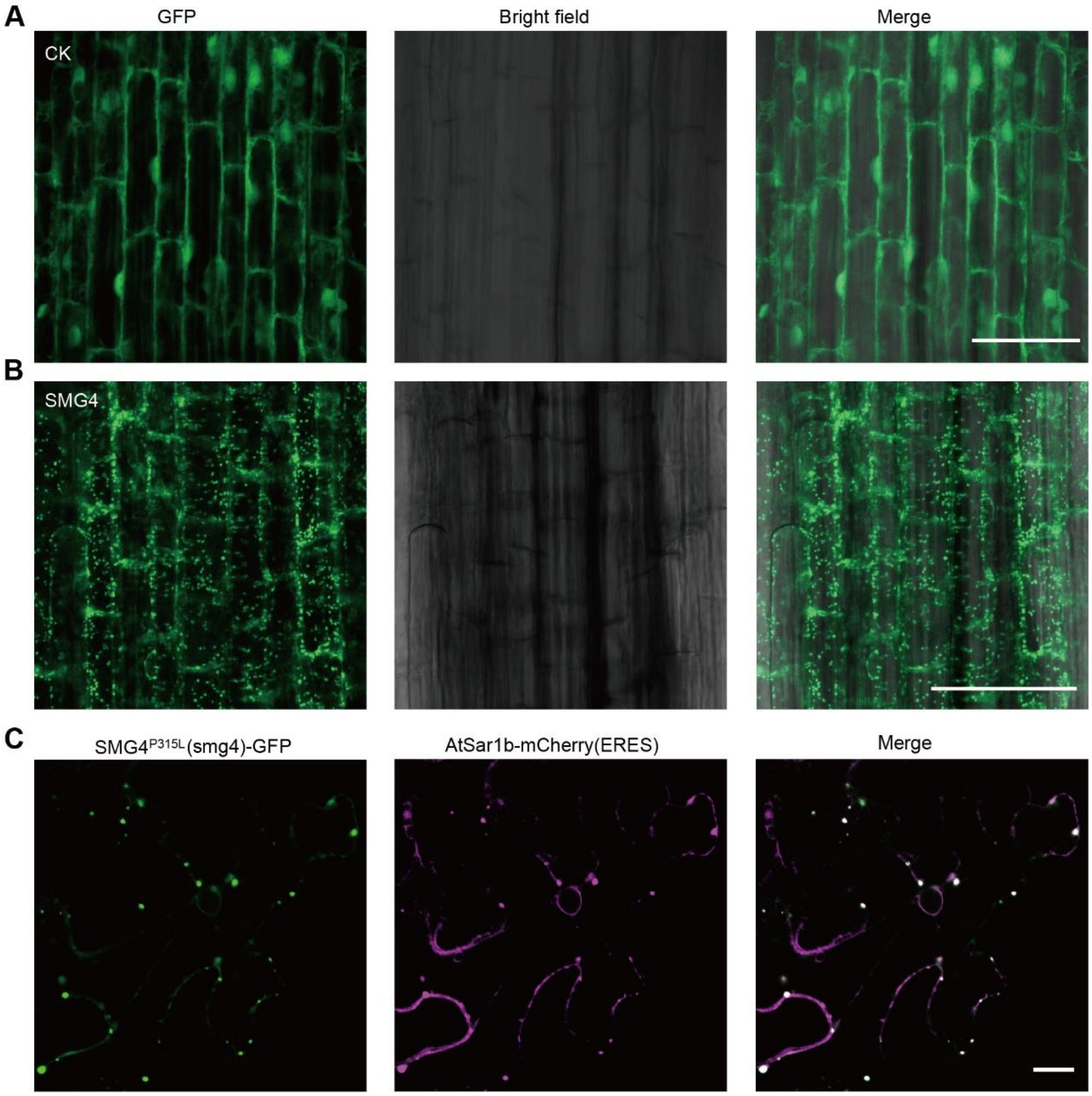

Supplemental Figure S6. Subcellular localization of SMG4 protein and smg4 mutant protein (Supports Figure 5)

(A, B) Subcellular localization of GFP (CK) **(A)** and the SMG4-GFP fusion protein **(B)** in the root cells of transgenic lines. Scale bars, 50 μm.

(C) Subcellular localization of smg4 (SMG4^{P315L}) mutant protein in the epidermal cells of *N. benthamiana* leaves. Scale bar, 20 μm.

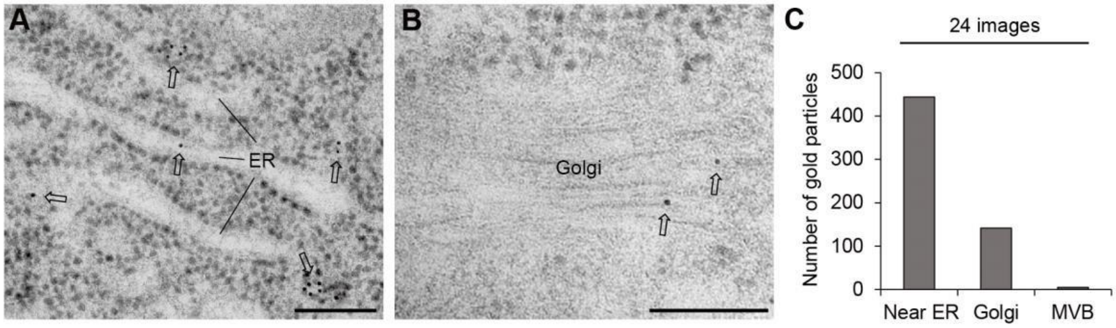

Supplemental Figure S7. Immunoelectron microscopy localization of SMG4-GFP (Supports Figure 5)
(A, B) Electron micrograph showing immunogold-labeled SMG4-GFP in root tip cells of transgenic rice plants. Gold particles are highlighted with arrows. Scale bars, 200 nm.
(C) Number of gold particles distributed near the ER, Golgi, and MVB.

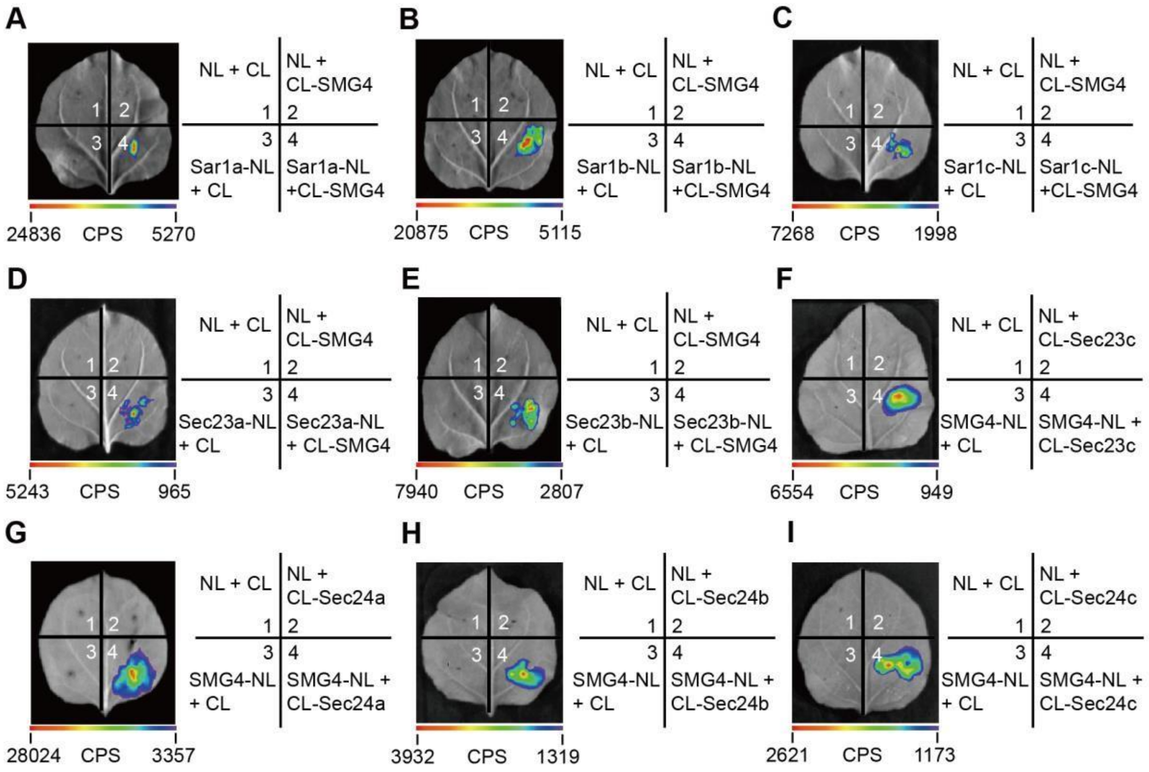

Supplemental Figure S8. LCI assays show that SMG4 interacts with the COPII components (Supports Figure 6)
(A–I) LCI assays showing that SMG4 interacts with the COPII components Sar1a **(A)**, Sar1b **(B)**, Sar1c **(C)**, Sec23a **(D)**, Sec23b **(E)**, Sec23c **(F)**, Sec24a **(G)**, Sec24b **(H),** and Sec24c **(I)** in *N. benthamiana* leaves cells. CL, C terminus of LUC; NL, N terminus of LUC. Colored scale bar indicates the luminescence intensity in counts per second (CPS).

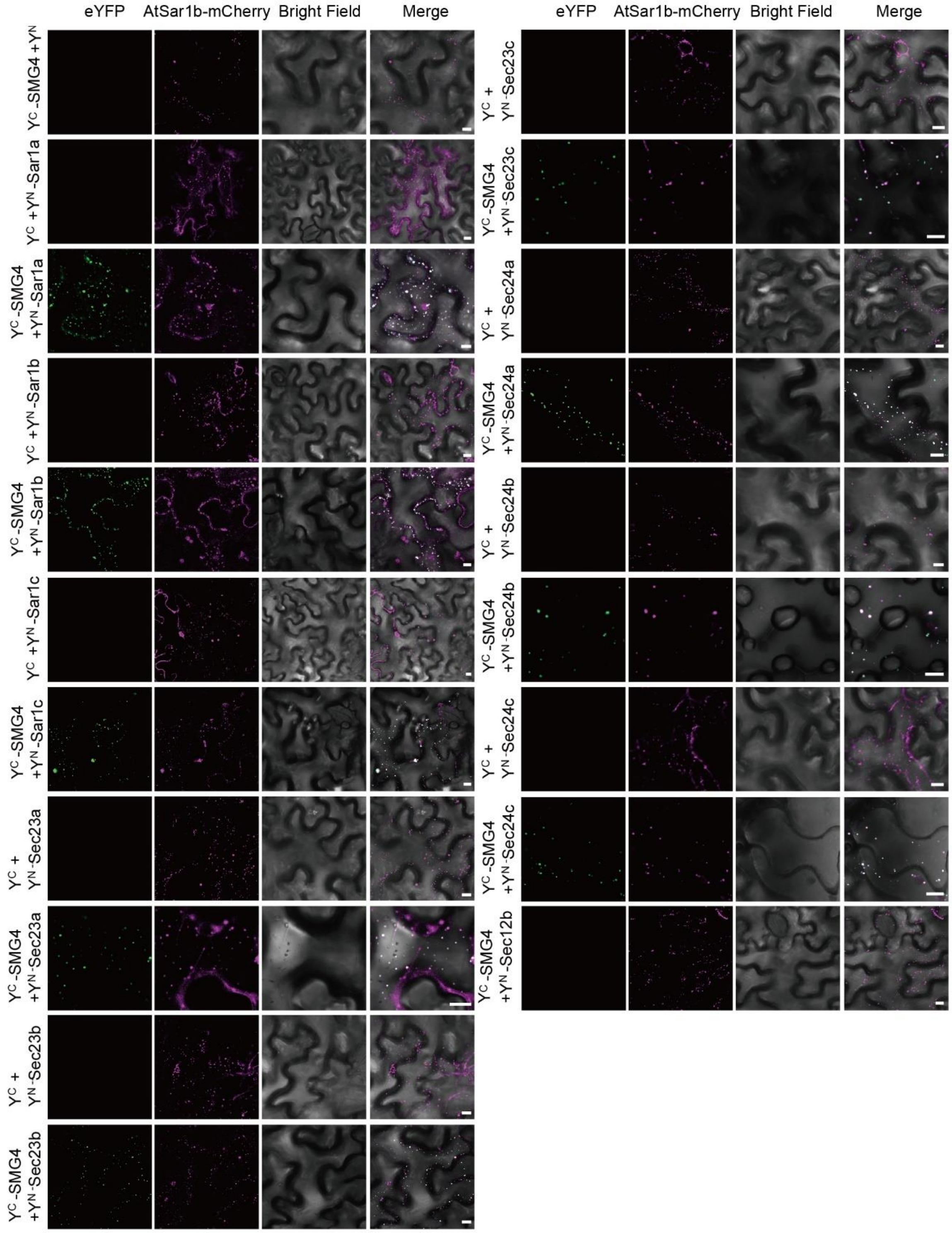

Supplemental Figure S9. BiFC assays show that SMG4 interacts with the COPII components (Supports Figure 6)

BiFC assays showing that SMG4 interacts with Sar1a, Sar1b, Sar1c, Sec23a, Sec23b, Sec23c, Sec24a, Sec24b, and Sec24c in *N. benthamiana* leaves cells. Scale bars, 10 µm. AtSar1b-mCherry is used as ERES marker. Sec12b was used as a negative control.

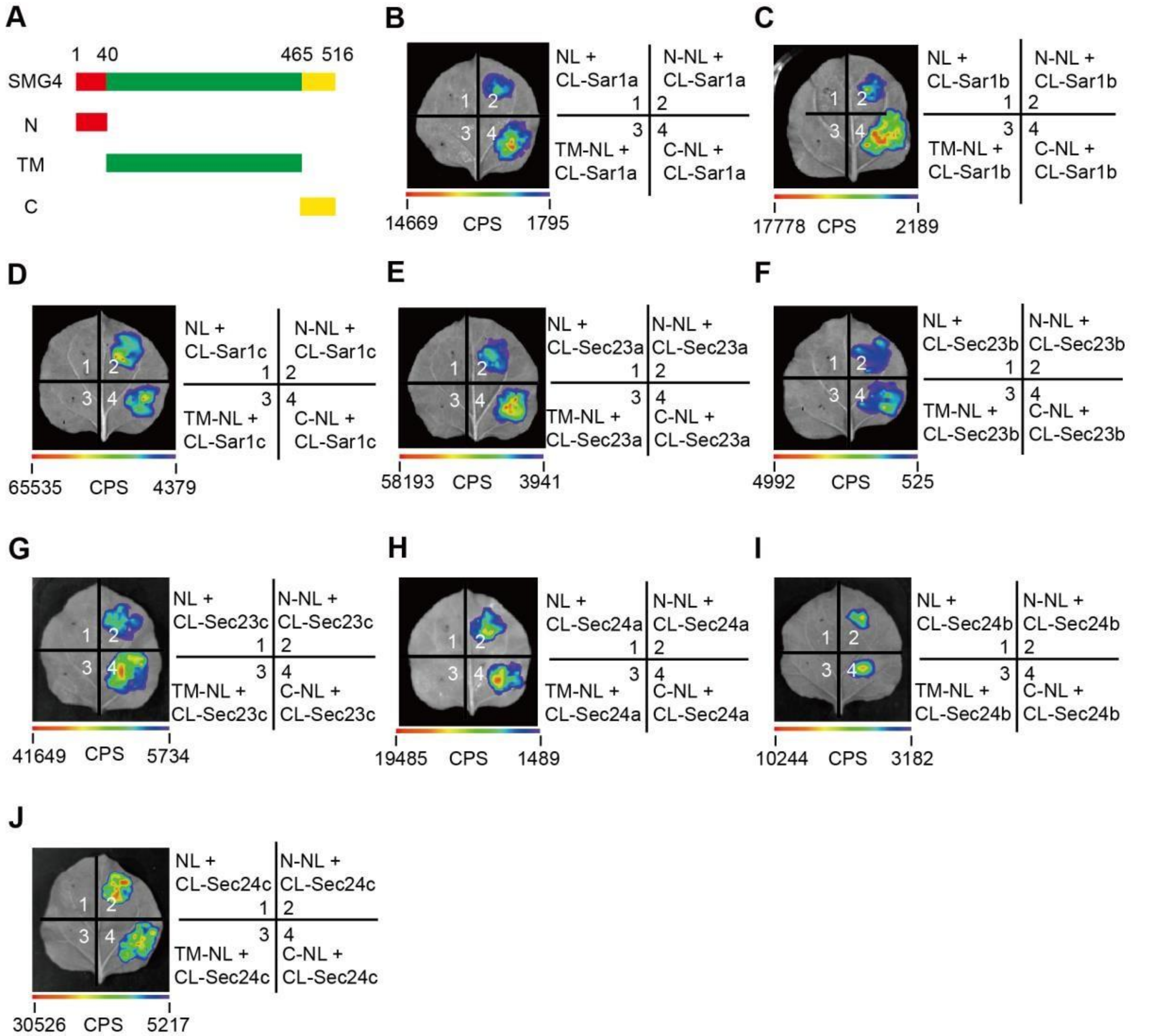

Supplemental Figure S10. The N- and C-terminal domains but not the transmembrane domain of SMG4 interacts with COPII components (Supports Figure 6)

(A) Diagrams of three truncated fragments of SMG4. N, TM, and C represent the N-terminal, transmembrane domain, and C-terminal regions of SMG4, respectively.

(B–J) Firefly LCI assays showing that the N-terminal and C-terminal fragments of SMG4 interact with Sar1a **(B)**, Sar1b **(C)**, Sar1c **(D)**, Sec23a **(E)**, Sec23b **(F)**, Sec23c **(G)**, Sec24a **(H)**, Sec24b **(I),** and Sec24c **(J)** in *N. benthamiana* leaves cells. CL, C terminus of LUC; NL, N terminus of LUC. Colored scale bar indicates the luminescence intensity in counts per second (CPS).

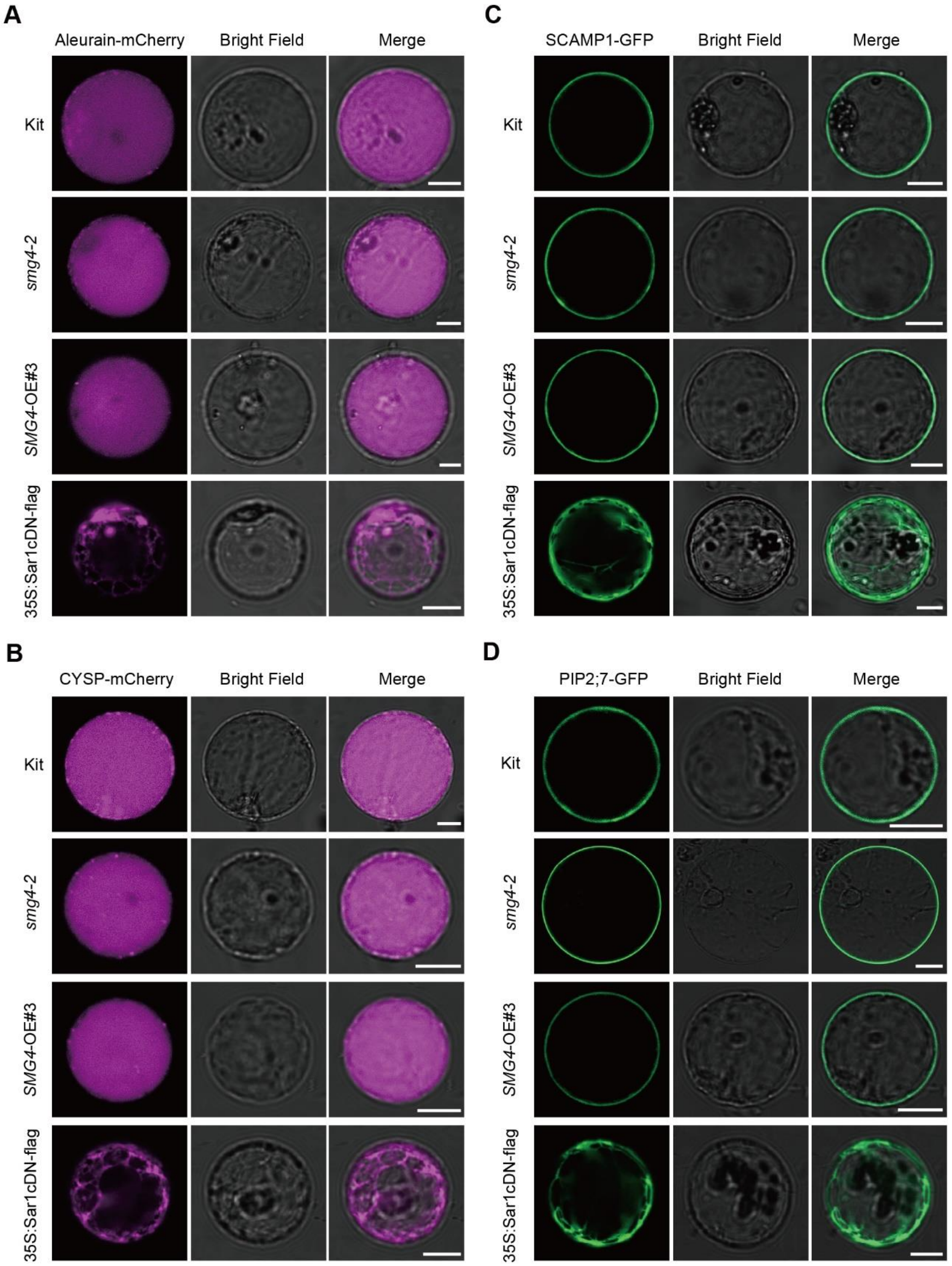

Supplemental Figure S11. SMG4 likely does not affect the transport function of COPII (Supports Figure 6)

(A, B) Subcellular localization of the vacuolar cargoes Aleurain-mCherry **(A)** and CYSP-mCherry **(B)** in protoplasts of Kit, *smg4-2*, and *SMG4*-OE#3.

(C, D) Subcellular localization of the plasma membrane cargoes SCAMP1GFP **(C)** and PIP2;7-GFP **(D)** in protoplasts of Kit, *smg4-2*, and *SMG4*-OE#3.
Scale bars, 10 μm. The Sar1c dominant-negative mutant (Sar1cDN)-flag was co-transfected with cargoes in protoplasts, used as control in A-D.

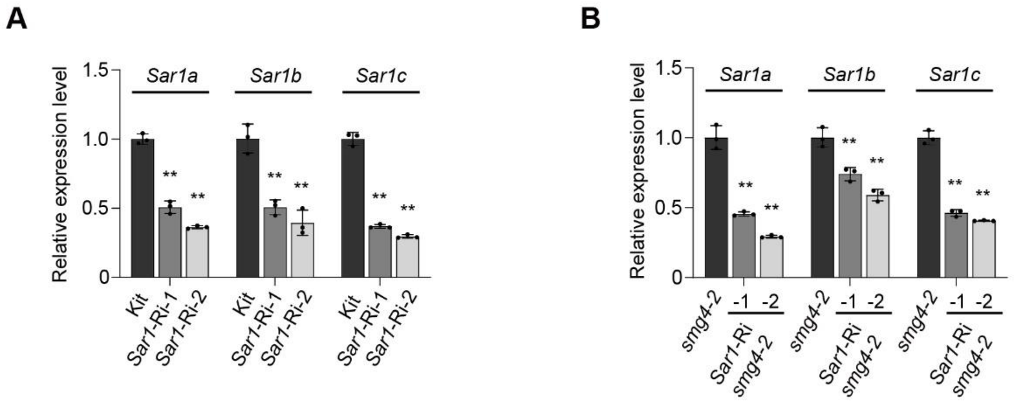

Supplemental Figure S12. Transcript levels of *Sar1s* in spikelet hulls of *Sar1* RNAi lines in Kitaake and *smg4-2* background (Supports Figure 6)

The *UBIQUITIN* gene was used as an internal control. Values are means ± SD (n = 3). Student's t-test was used to calculate the P values, **P <0.01.

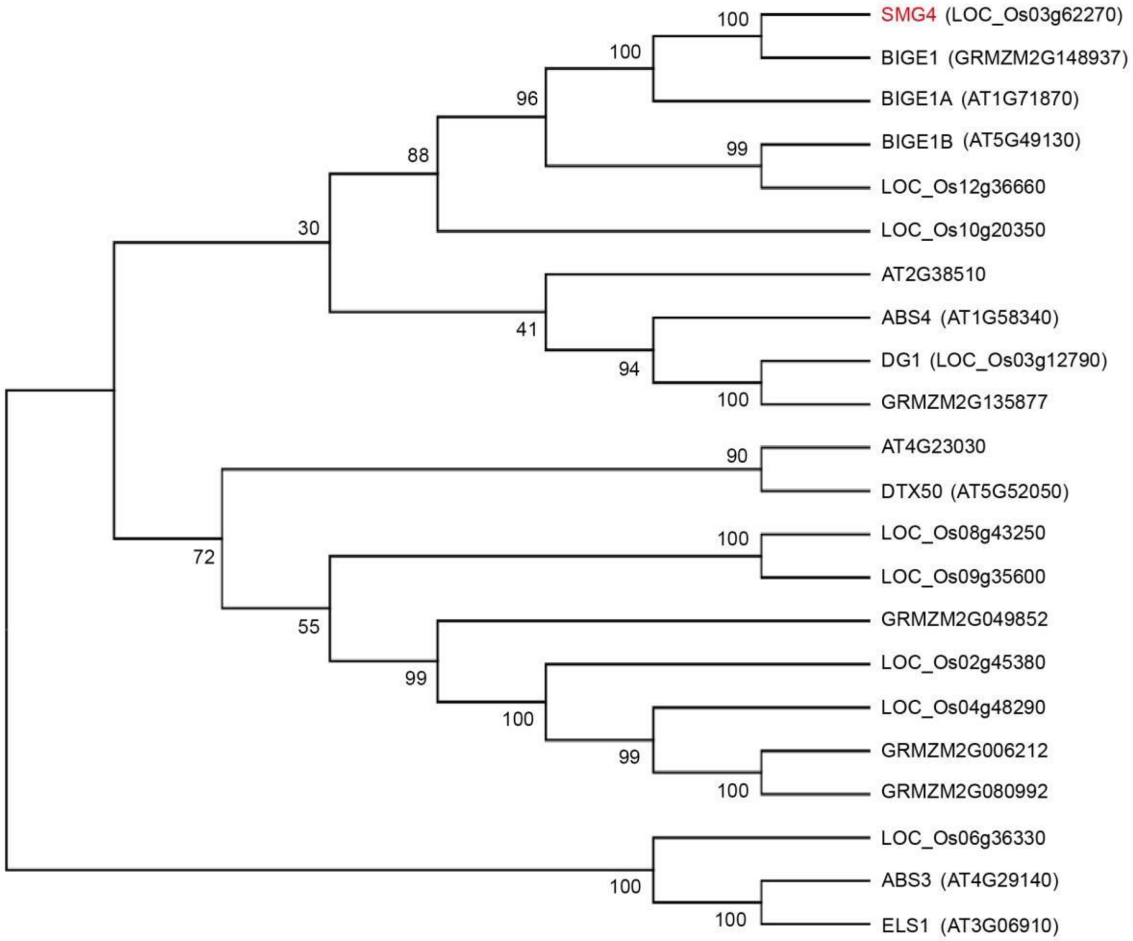

Supplemental Figure S13. Phylogenic analysis of SMG4 and its homologs (Supports Figure 7)
The phylogenic tree was reconstructed using MEGA version 7.0. SMG4 is highlighted in red font.

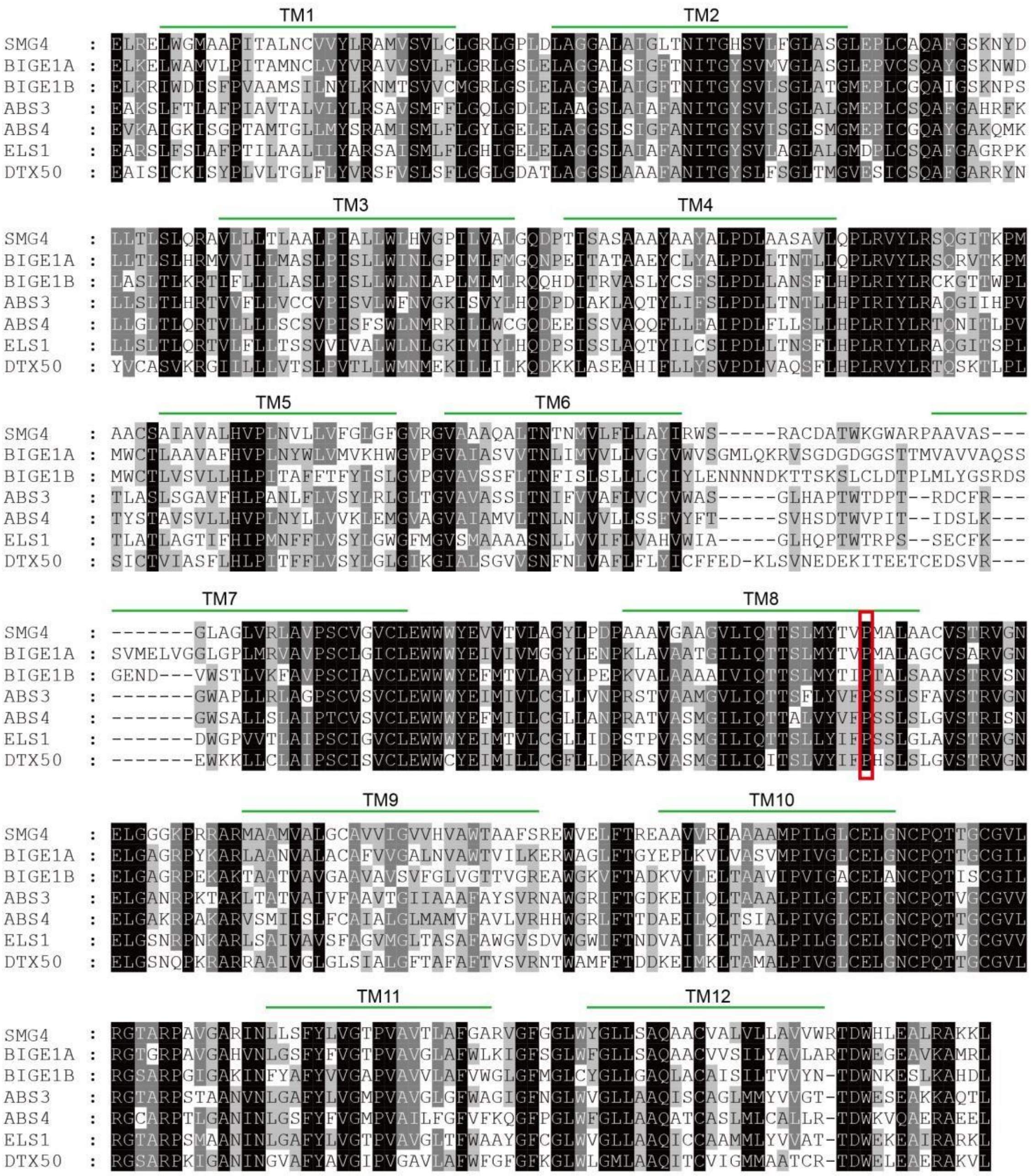

Supplemental Figure S14. Sequence alignment of SMG4 and its six Arabidopsis homologs (Supports Figure 7)

Green lines indicate the twelve transmembrane domains predicted by HMMTOP Server v. 2.0 (http://www.enzim.hu/hmmtop/html/submit.html). Mutated amino acid in smg4 ($SMG4^{P315L}$) mutant protein is highlighted in the red box.

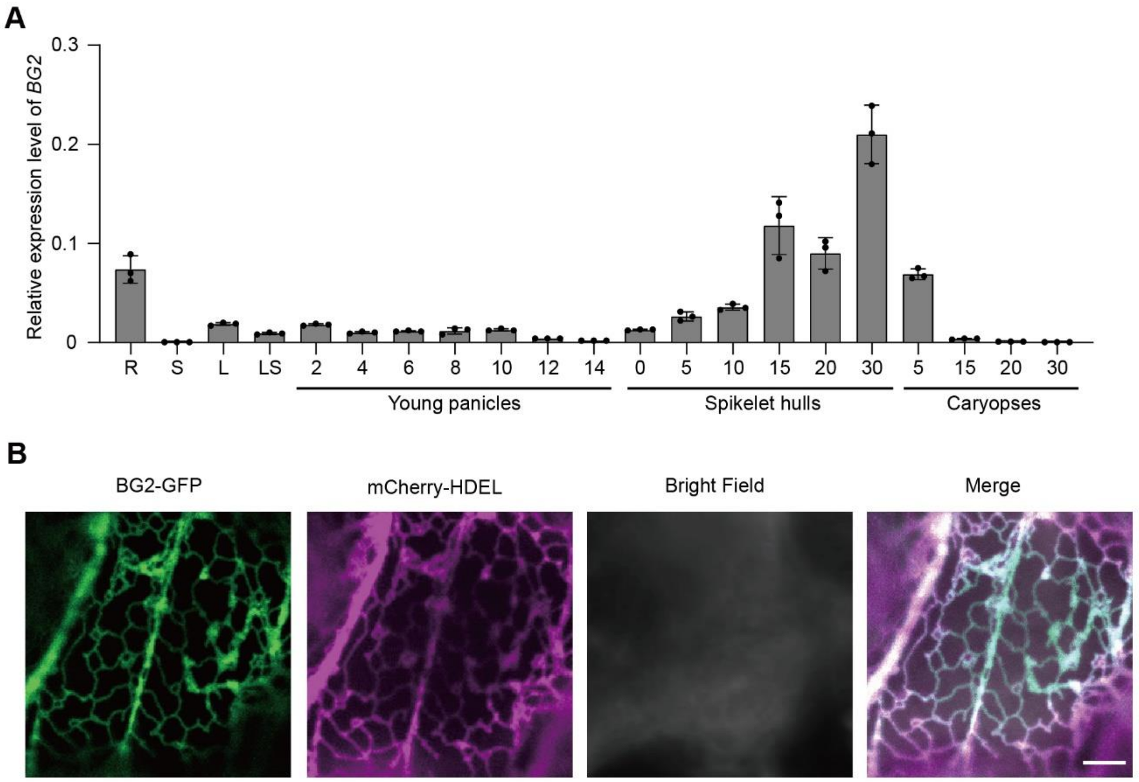

Supplemental Figure S15. Expression pattern and subcellular localization of *BG2* gene (Supports Figure 7)

(A) Relative *BG2* expression levels in different plant tissues analyzed by RT-qPCR. Plant tissues including roots (R), stems (S), leaves (L), leaf sheaths (LS), young panicles (numbers indicate the length of young panicles, in cm), spikelet hulls (BH, before heading; numbers indicate the days after heading), and caryopses (numbers indicate the days after heading) of 9311 (n = 3). The *UBIQUITIN* gene was used as an internal control. Values are means ± SD.

(B) Subcellular localization of BG2-GFP fusion protein in *N. benthamiana* leaf cells. mCherry-HDEL was used as ER marker. Scale bars, 10 μm.

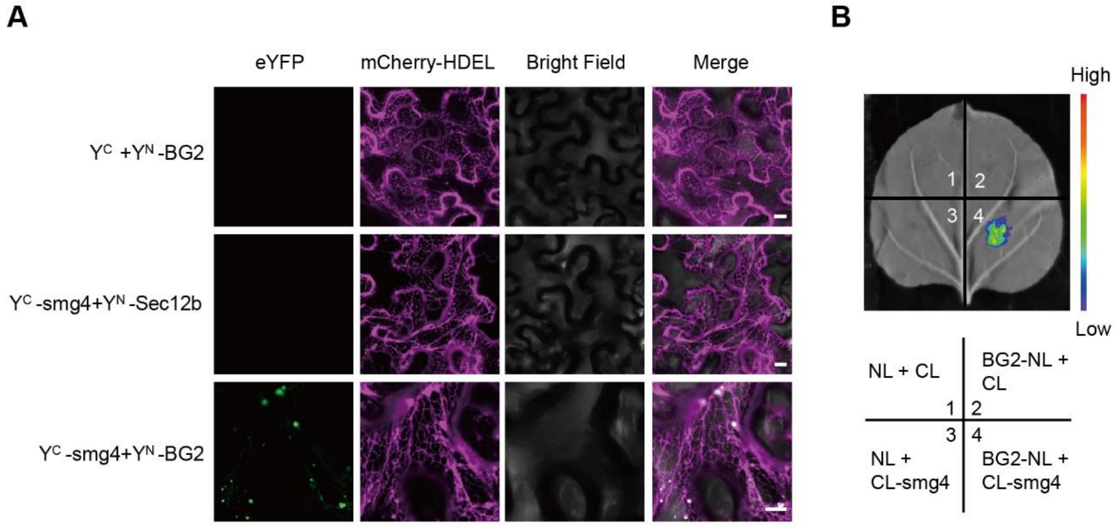

Supplemental Figure S16. smg4 interacts with BG2 (Supports Figure 7)
(A) BiFC assay shows that smg4 (SMG4^{P315L}) can interact with BG2 in *N*. *benthamiana* leaf cells. Scale bars, 10 μm. Sec12b was used as a negative control.
(B) Firefly LCI assay showing the interaction between smg4 (SMG4^{P315L}) and BG2 in *N*. *benthamiana* leaf cells. CL, C terminus of LUC; NL, N terminus of LUC. Colored scale bar indicates the luminescence intensity in counts per second.

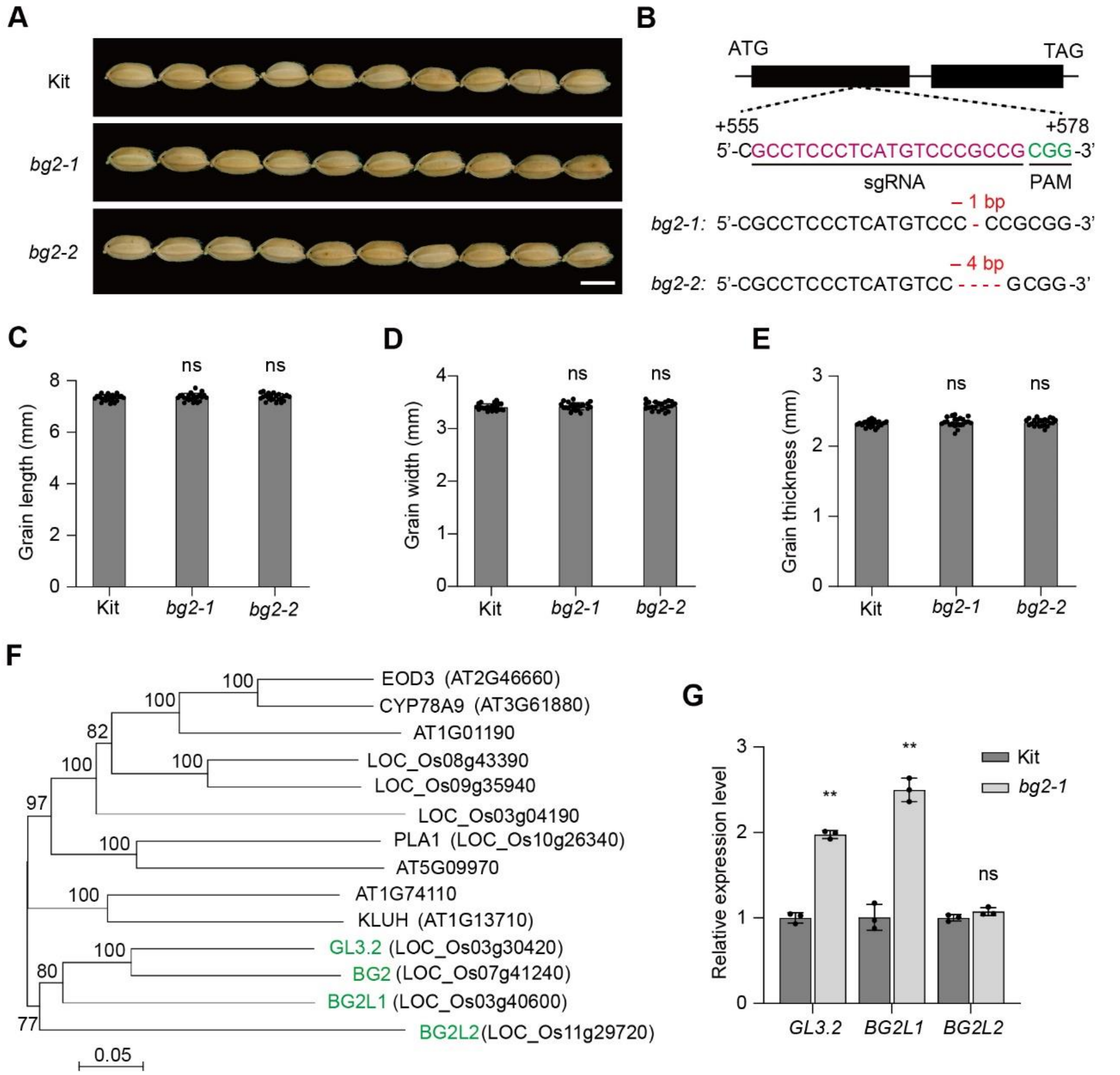

Supplemental Figure S17. BG2 likely functions redundantly with its homologs to regulate grain size (Supports Figure 8)

(A) Grain morphologies of Kit and *BG2* knockout lines (*bg2-1* and *bg2-2*). Scale bar, 5 mm.

(B) Identification of *BG2* knockout lines generated by the CRISPR/Cas9 technology. The sgRNA-targeted site and PAM are indicated in magenta and green font, respectively. Red dashed line represents deleted nucleotides.

(C–E) Grain length (*n* = 22) **(C)**, grain width (*n* = 22) **(D)**, and grain thickness (*n* = 22) **(E)** of Kit and *BG2* knockout lines (*bg2-1* and *bg2-2*).

(F) Phylogenic tree of the BG2 protein and its homologs in *Oryza sativa* and *Arabidopsis thaliana*. BG2 and its homologs GL3.2, BG2L1, and BG2L2 highlighted in green font. The phylogenic tree was reconstructed using MEGA version 7.0.

(G) Relative transcript levels of *GL3.2*, *BG2L1*, and *BG2L2* in spikelet hulls of Kit and *bg2-1* (*n* = 3). The *UBIQUITIN* gene was used as an internal control.

Values are means ± SD. Student's *t*-test was used to calculate the *P* values, $^{**}P$ <0.01. ns, no significance.

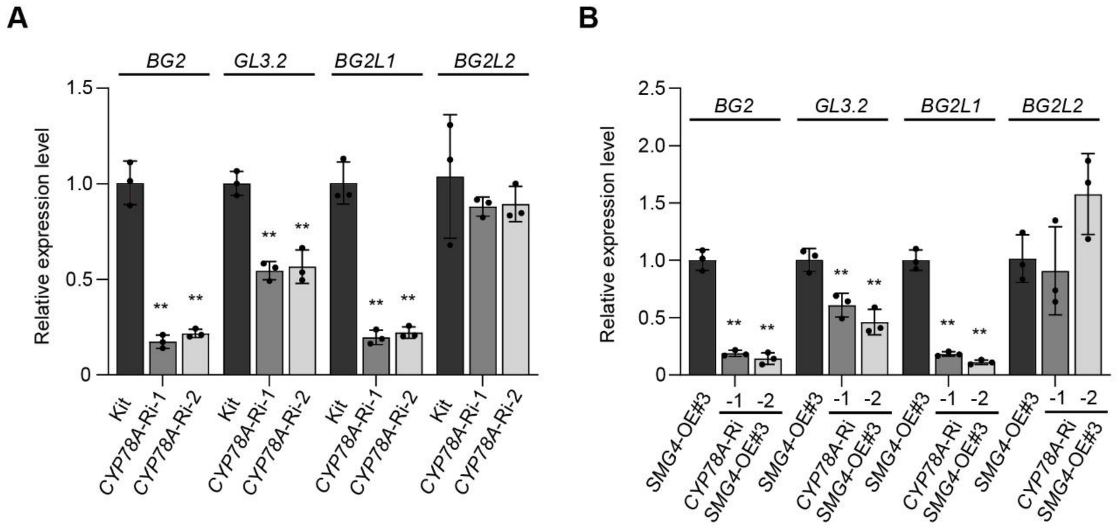

Supplemental Figure S18. Transcript levels of *CYP78As* in spikelet hulls of *CYP78A* RNAi lines in Kitaake and *SMG4*-OE#3 background (Supports Figure 8)
The *UBIQUITIN* gene was used as an internal control. Values are means ± SD (*n* = 3). Student's *t*-test was used to calculate the *P*-values, ***P* <0.01.

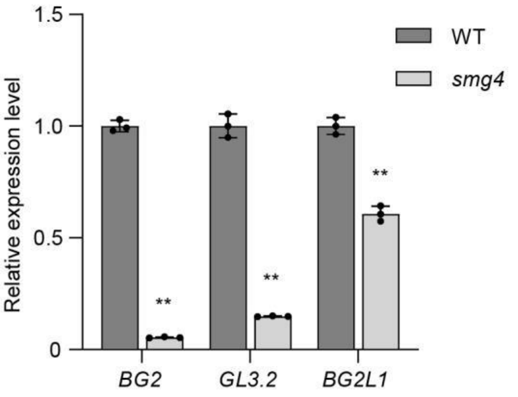

Supplemental Figure S19. The transcript levels of *CYP78A*s are lower in the *smg4* mutant (Supports Figure 8)
Relative transcript levels of *CYP78A*s (*BG2*, *GL3.2*, and *BG2L1*) in spikelet hulls of WT and the *smg4* mutant. The *UBIQUITIN* gene was used as an internal control. Values are means ± SD (*n* = 3). Student's *t*-test was used to calculate the *P*-values, ***P* <0.01.

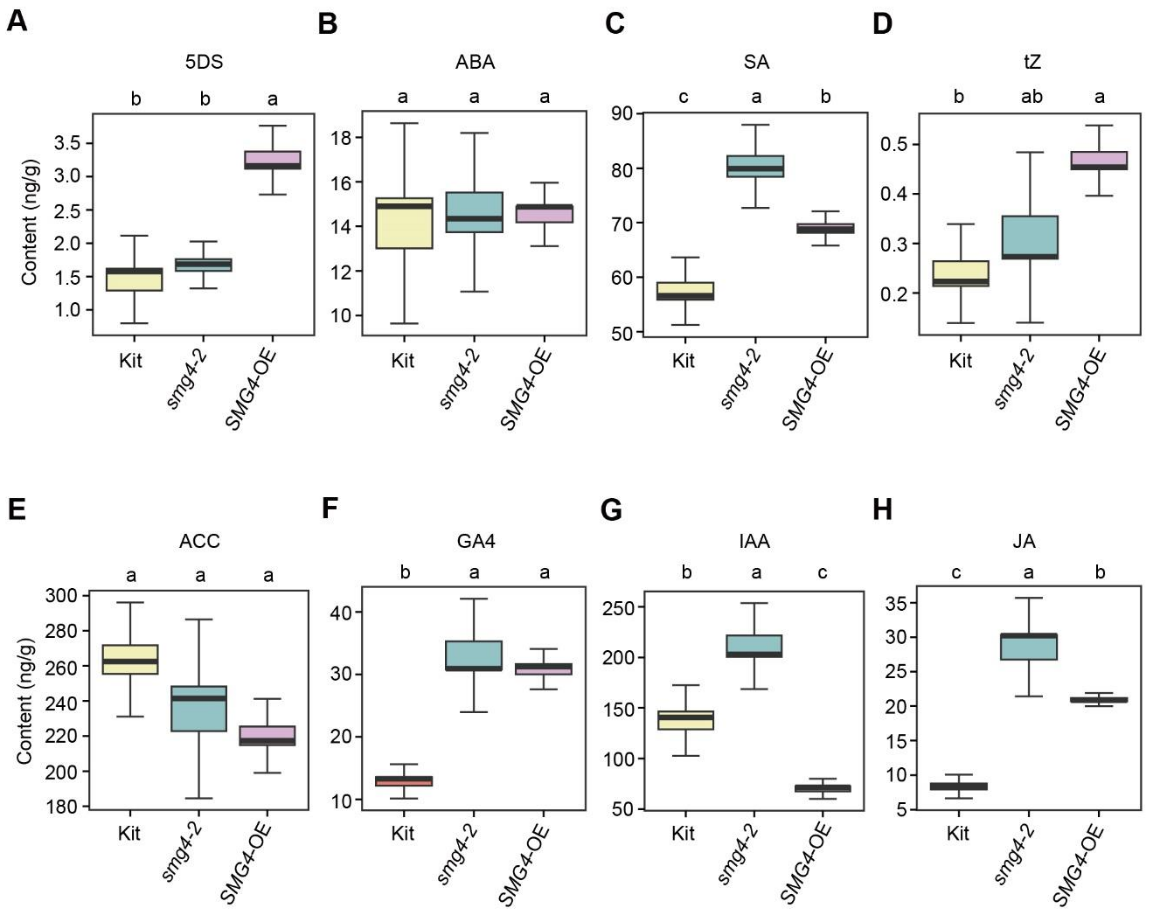

Supplemental Figure S20. Contents of eight plant hormones in spikelet hulls of Kitaake, *smg4-2*, and *SMG4*-OE (Supports Figure 10)

(A–H) Contents of 5DS **(A)**, ABA **(B)**, SA **(C)**, tZ **(D)**, ACC **(E)**, GA4 **(F)**, IAA **(G)**, and JA **(H)** in spikelet hulls of Kit, *smg4-2*, and *SMG4*-OE before heading. Three biological replicates were performed. Values are means ± SD. Different letters indicate significant differences ranked by pairwise multiple comparison followed with Tukey's test ($P < 0.05$).

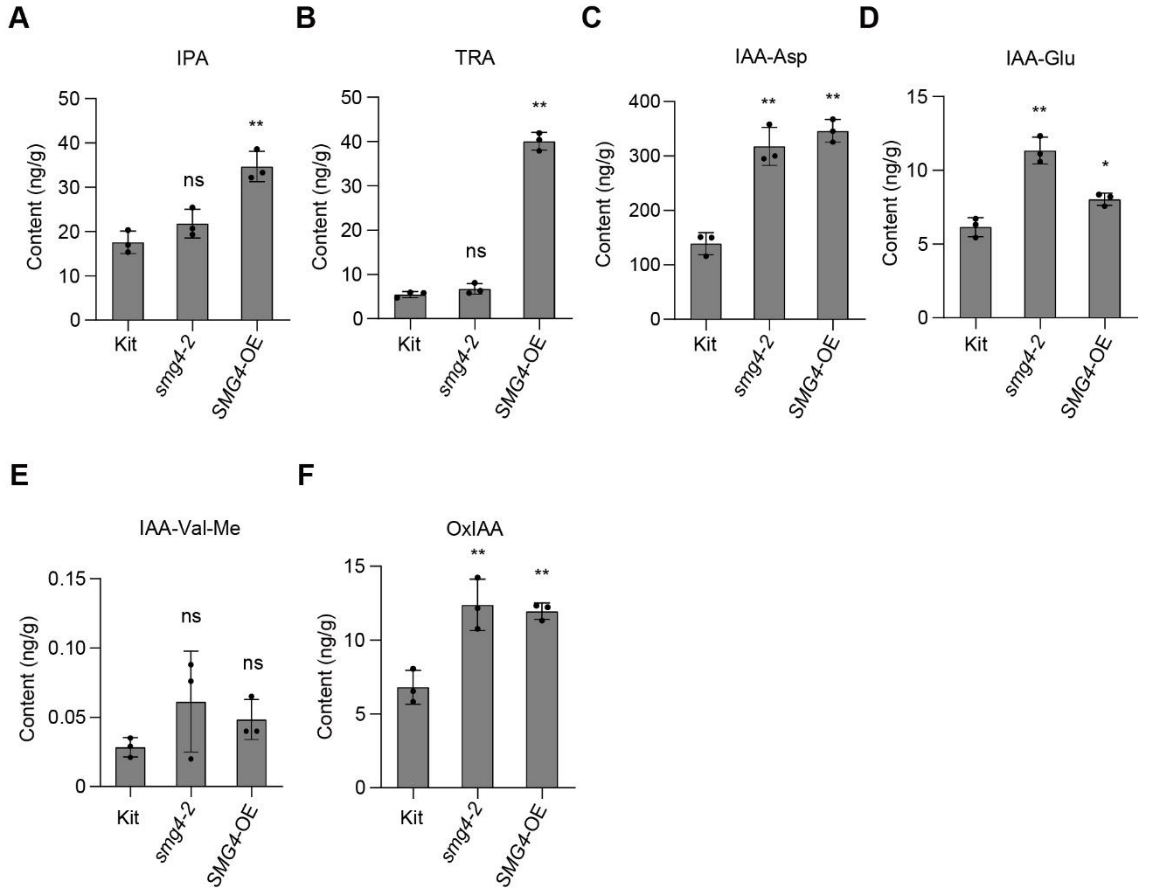

Supplemental Figure S21. Contents of auxin biosynthesis precursors, bound auxins and oxidized auxins in spikelet hulls of Kitaake, *smg4-2*, and *SMG4*OE (Supports Figure 10)
(A–F) Contents of auxin biosynthesis precursors such as IPA (3-Indolepropionic acid) **(A)** and TRA (Tryptamine) **(B)**, bound auxins such as IAA-Asp (Indole-3acetyl-L-aspartic acid) **(C)**, IAA-Glu (Indole-3-acetyl glutamic acid) **(D)**, and IAAVal-Me (Indole-3-acetyl-L-valine methyl ester) **(E)**, and oxidized auxin OxIAA (2oxindole-3-acetic acid) **(F)** in spikelet hulls of Kit, *smg4-2*, and *SMG4*-OE before heading. Three biological replicates were performed. Values are means ± SD. Student's *t*-test was used to generate the *P* values, ***P* <0.01, **P* <0.05. ns, no significance.

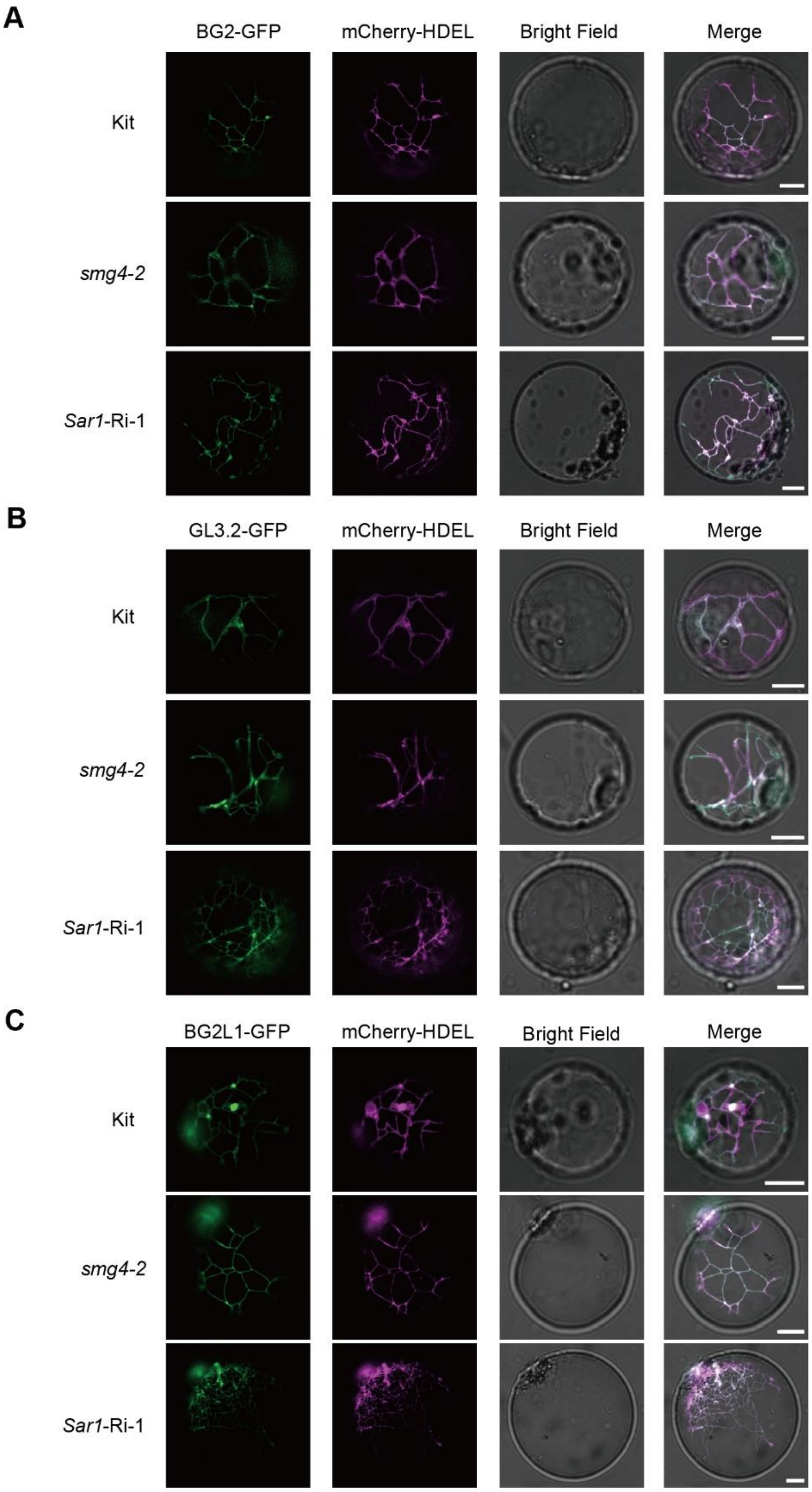

Supplemental Figure S22. Subcellular localization of BG2, GL3.2, and BG2L2 in protoplasts of Kitaake, *smg4-2*, **and** ***Sar1*****-Ri-1** (Supports Figure 10)
mCherry-HDEL is used as ER marker. Scale bars, 10 µm.

Supplemental Table S1. Important agronomic traits between WT and the *smg4* mutant

Agronomic trait	WT	*smg4*
Plant height (cm)	119.85 ± 4.11	89.05 ± 3.14 **
Tiller Number	6.36 ± 0.63	6.74 ± 1.48
Primary branch number	10.10 ± 0.57	10.30 ± 0.67
Secondary branch number	50.40 ± 5.70	50.00 ± 6.99
Grain number per main panicle	231.13 ± 6.48	235.13 ± 7.89
Grain length (mm)	9.68 ± 0.30	7.95 ± 0.32 **
Grain width (mm)	2.71 ± 0.04	2.38 ± 0.06 **
Grain thickness (mm)	2.15 ± 0.02	1.90 ± 0.03 **
Brown rice length (mm)	7.16 ± 0.11	6.03 ± 0.12 **
Brown rice width (mm)	2.51 ± 0.04	2.17 ± 0.05 **
Brown rice thickness (mm)	1.99 ± 0.05	1.77 ± 0.05 **
Thousand-grain weight (g)	32.67 ± 0.47	21.47 ± 0.11 **

Values are means ± SD. Student's *t*-test was used to calculate the *P*-values, $^{**}P < 0.01$.

Supplemental Table S2. Phenotypic segregation in reciprocal crosses between WT and the *smg4* mutant

Cross	Normal	Small grain	X^2 (3:1)
WT/*smg4*	156	51	0.015
smg4/WT	177	56	0.116

Supplemental Table S3. Identification of 16 SNPs between WT and *smg4* mutant within the fine mapping region

NO.	POS	REF	ALT	Gene	Annotation
SNP1	39618706	G	A	LOC_Os03g62260	intergenic
SNP2	39623347	C	T	LOC_Os03g62270	exonic
SNP3	39731033	T	C	LOC_Os03g62430	intergenic
SNP4	39763130	G	A	LOC_Os03g62510	upstream
SNP5	39763132	C	A	LOC_Os03g62510	upstream
SNP6	39776256	C	A	LOC_Os03g62522	downstream
SNP7	39776345	C	T	LOC_Os03g62522	downstream
SNP8	39783542	A	G	LOC_Os03g62539	downstream
SNP9	39804638	C	T	LOC_Os03g62580	upstream
SNP10	39804668	G	T	LOC_Os03g62580	upstream
SNP11	39804677	C	T	LOC_Os03g62580	upstream
SNP12	39817155	C	T	LOC_Os03g62610	upstream
SNP13	39868321	G	A	LOC_Os03g62700	intergenic
SNP14	39869010	G	T	LOC_Os03g62700	intergenic
SNP15	39871323	T	C	LOC_Os03g62700	intergenic
SNP16	39871363	T	C	LOC_Os03g62700	intergenic

POS, position in chromosome; REF, nucleotide in WT; ALT, nucleotide in *smg4*. SNP2 is highlighted in red font.
