# Supplementary material for: A CYP78As–small grain4–coat protein complex Ⅱ pathway promotes grain size in rice
Source: Plant Cell. 2023 Sep 21;35(12):4325–46. doi: 10.1093/plcell/koad239 (PMC10689148; doi:10.1093/plcell/koad239)
Supplement: koad239_Supplementary_Data [file koad239_supplementary_data.zip › TPC2022RA00268D_Supplemental_Movie_Legend.pdf]

Supplemental Movie S1. Time-lapse microscopy of SMG4-GFP in the leaf epidermal cells of *N. benthamiana.*
