# Supplementary material for: A CYP78As–small grain4–coat protein complex Ⅱ pathway promotes grain size in rice
Source: Plant Cell. 2023 Sep 21;35(12):4325–46. doi: 10.1093/plcell/koad239 (PMC10689148; doi:10.1093/plcell/koad239)
Supplement: koad239_Supplementary_Data [file tpc22.00268.PeerReviewReport.pdf › tpc22.00268.PeerReviewReport.pdf]

A CYP78As–SMG4–COP Ⅱ pathway promotes grain size in rice

Chunlei Zhou, Qibing Lin, Yulong Ren, Jie Lan, Rong Miao, Miao Feng, Xin Wang, Xi Liu, Shengzhong Zhang, Tian Pan, Jiachang Wang, Sheng Luo, Jinsheng Qian, Wenfan Luo, Changling Mou, Thanhliem Nguyen, Zhijun Cheng, Xin Zhang, Cailin Lei, Shanshan Zhu, Xiuping Guo, Jie Wang, Zhichao Zhao, Shijia Liu, Ling Jiang, and Jianmin Wan

Corresponding authors: Jianmin Wan (wanjm@njau.edu.cn or wanjianmin@caas.cn) and Ling Jiang (jiangling@njau.edu.cn).

Review timeline:

TPC2022-RA-00157	Submission received:<br>1st Decision:	Feb. 10, 2022<br>Mar. 16, 2022 *manuscript declined*
**TPC2022-RA-00268**	Submission received:<br>1st Decision:<br>Final acceptance:	July 5, 2023<br>Aug. 11, 2023 *acceptance pending, sent to science editor*<br>Aug. 30

REPORT: (The report shows the major requests for revision and author responses. Minor comments for revision and miscellaneous correspondence are not included. The original format may not be reflected in this compilation, but the reviewer comments and author responses are not edited, except to correct minor typographical or spelling errors that could be a source of ambiguity.)

TPC2022-RA-00157 1st Editorial decision – *declined* Mar. 16, 2022

Thank you for choosing to send your manuscript entitled "The MATE transporter Small Grain 3 increases grain size by mediating ER-Golgi transport of the CYP78A signal" for consideration at The Plant Cell. Your submission has been evaluated by members of the editorial board as well as expert reviewers in your field, and we regret to inform you that we are not able to recommend publication of this manuscript in its current form. We have not made this decision lightly. We have had input from multiple scientists, and we have solicited post-review comments as well. Our present policy is to offer streamlined decisions and to not advise on the direction of the work by requesting extensive modifications or substantial additional experiments.

That said, we would be willing to re-consider a new manuscript that fully addressed the concerns raised during this review process. If you decide to resubmit to The Plant Cell, it will be evaluated as a new submission subject to full assessment by the editorial board, and if sent for external review, a new set of reviewers might be chosen. Reviewers will be asked to assess your work as a new manuscript (i.e. are the claims fully supported by the data and do the results presented move the field forward?), and not only whether previous reviewer comments have been addressed.

As you will read, the major concern is that further experimental work is required to provide evidence to support the hypotheses that SMG3 functions in COPII dependent trafficking and/or BG2 signaling.

-- Reviewer comments:

TPC2022-RA-00268 Submission received July 5, 2023

Reviewer comments on previously declined manuscript and **author responses:**

<u>Reviewer #1:</u>

In this newly submitted manuscript, Zhou and co-authors used the forwards genetic screening method to identify the SMG3, which encodes a multidrug and toxic compound extrusion (MATE) transporter, as a new regulator for grain size in rice. Interestingly, SMG3 co-localizes with the endoplasmic reticulum exit sites and interacts with COP Ⅱ components. Further, SMG3 interacts with BG2 and functions together to regulate grain size. Their findings suggest that SMG3-COP Ⅱ potentially mediates ER-Golgi transport of the CYP78A signal, revealing the BG2-SMG3-COP Ⅱ

regulatory pathway of grain size in rice. In general, this study is novel and of great interests, showing the importance of endomembrane trafficking in regulating grain (seed) size. The genetic data are very solid, which are supported by the biochemical or cellular analysis. Below, please see my suggestion for revising or improving the manuscript:

Response: Thank you very much for the positive comments on our manuscript. All comments are valuable and helpful for improving our manuscript. Please pay attention to the two changes in our revised version: "SMG3" to "SMG4", as "SMALL GRAIN 3 (SMG3)" was recently used in a Plant Cell paper in December, 2022 (Li et al., 2022); the title is changed to "A CYP78As–SMG4–COP Ⅱ pathway promotes grain size in rice" for a clearer description on our findings.

Major points:

Q1. The grain thickness in OE1 lines are not significantly different from WT, while OE2 lines are thicker than WT as shown in Figure 3K. Did the author has other OE line to prove that overexpressing the SMG3 will increase the thickness of the grain? Alternatively, please include a possible discussion on this difference.

Response: Thank you very much for the suggestion. We analyzed the phenotypes of two other SMG4 overexpression lines (*SMG4*-OE#3 and *SMG4*-OE#4), whose expression levels were higher than *SMG4*-OE#1 (Figure R1). They exhibited larger grains as a result of increase in grain length and thickness, although no change in grain width, compared to the wild type Kitaake (Table R1). *SMG4*-OE#3 was used to replace the original *SMG4*-OE#1 in Figure 3 (Figures 3G, 3I-3K).

	Kitaake	*SMG4*-OE#3	*SMG4*-OE#4
Grain length (mm)	7.13 ± 0.16	8.73 ± 0.19 **	8.94 ± 0.12 **
Grain width (mm)	3.58 ± 0.08	3.54 ± 0.12	3.61 ± 0.07
Grain thickness (mm)	2.54 ± 0.04	2.61 ± 0.07 **	2.62 ± 0.06 **

Table R1 for Review only. Grain size statistics of two other SMG4 overexpression lines.

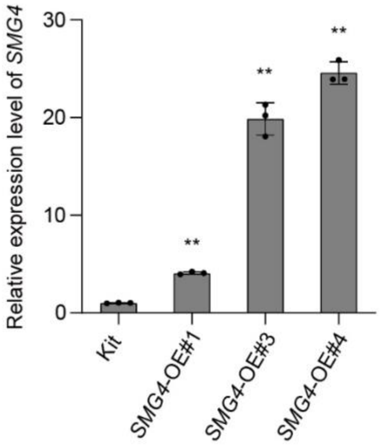

Figure R1 for Review only. Transcript levels of *SMG4* in spikelet hulls of SMG4 overexpression lines.

The *UBIQUITIN* gene was used as an internal control. Kit, Kitaake. All data shown are mean ± SD (*n* = 3). Student's *t*-test was used to generate the *P* values, ***P* <0.01.

Q2. Sec12 is an integral membrane protein, which should not be solubilized by high salt. I would suggest the authors to carefully repeat the experiment or use other ER-resident protein as control in Figure 4C.

Response: Thank you very much for the suggestion. Following your suggestion, we performed the experiment with

tonoplast intrinsic protein γTIP as the control. The result showed that, like γTIP, SMG4 could not be extracted with high salt or alkali but could be partly solubilized with Triton X-100 and efficiently solubilized with SDS (Figure 4C).

Q3. It would be nice if the authors could quantify the gold particles in Figure 5 to further support their conclusion.

Response: Thank you very much for the suggestion. Following your suggestion, we quantified the gold particles in Figure 5. The result showed that gold particles were mainly enriched near the ER membrane, with fewer seen on the Golgi (Figures 5F–5I and Supplemental Figure S7). (Line 236-238).

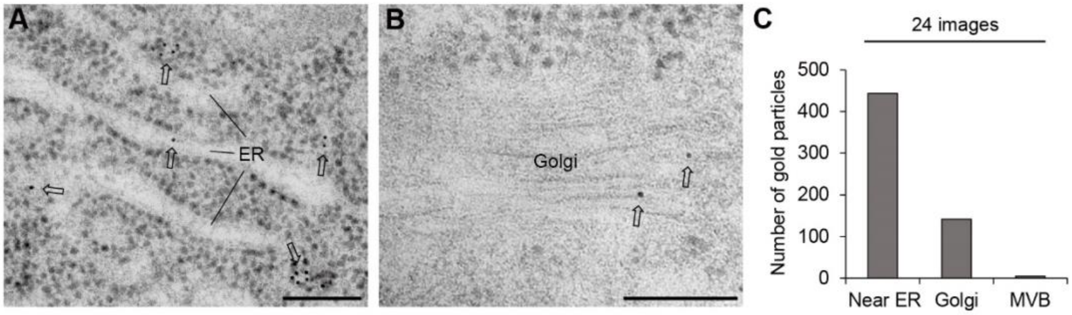

Supplemental Figure S7. Immunoelectron microscopy localization of SMG4-GFP.

(A) and **(B)** Electron micrograph showing immunogold-labeled SMG4-GFP in root tip cells of transgenic rice plants. Gold particles are pointed with arrows. Bars = 200 nm. **(C)** Number of gold particles observed near ER, Golgi, and MVB.

Q4. Although the data showing both N or C terminus contain the signal for SMG3 localization is of potential interests, I would suggest to delete this data since it is too weak to give any conclusion with a single confocal image.

Response: Thank you very much. Following your suggestion, this data has been removed in our revised manuscript.

Q5. Ito et al., 2021 MBoC have shown that BFA treatment did not obviously alter the puncta pattern of ERES, causing the discrepancy between results from different groups. Alternatively, the author could use the H89 to demonstrate the bona fide ERESs localization of the SMG3 (Langhans et al., 2007, Plant Physiology; Zeng et al., 2021, PNAS).

Response: Thank you very much for the valuable suggestion. We treated *SMG4-GFP* transgenic seedlings with H89 and found that the puncta labeled by SMG4-GFP significantly decreased with H89 treatment compared with the untreated control (Figures 6A and 6B). The result further supports the notion that SMG4 is an ERES-localized protein. (Line 243-248).

Q6. As stated by the authors, indeed different phytohormones including ABA is known to be important for grain growth, while several MATE proteins were reported to be involved in ABA transport. Recent study (Li et al., 2021 Nature Plants) has shown that a type of giant COPⅡ is involved in ABA related channel or transporter trafficking under abiotic stresses. Interestingly, the authors showed that SMG3 could interact with AtSar1a (the key player for the giant COPⅡ formation), would the SMG3 also participate in the formation of giant COPⅡ vesicles for stress responses in rice?

Response: Thank you very much for the suggestion. Following your suggestion, we performed several experiments to investigate whether SMG4 participates in the formation of giant COPⅡ vesicles in rice. First, we tested the content of ABA in the spikelet hulls of wild-type (WT) Kitaake, *SMG4*-CR, and *SMG4*-OE, and found that there was no difference between them (Supplemental Figure S20B), which indicates that ABA is unlikely the cargo transported by SMG4. Further, we analyzed the localization of the vacuolar cargoes Aleurain-mCherry and CYSP-mCherry as well as the plasma membrane cargoes SCAMP1-GFP and PIP2;7-GFP, which were transported by COPⅡ, in protoplasts of WT, *SMG4*-CR#1, and *SMG4*-OE#3, respectively. The results showed that the vacuolar cargoes (Aleurain-mCherry and CYSP-mCherry) and the plasma membrane cargoes (SCAMP1-GFP and PIP2;7-GFP) were delivered normally to

the vacuole and plasma membrane in both *SMG4*-CR#1 and *SMG4*-OE#3, respectively (Supplemental Figure S11), indicating that COP Ⅱ -mediated ER–Golgi secretion route is not obviously disrupted in both *SMG4*-CR#1 and *SMG4*-OE#3. However, our study also found that SMG4 interacts with COP Ⅱ components (Supplemental Figures S8, S9 and Figure 6C). Therefore, we speculated that the transmembrane protein SMG4 might function as a cargo receptor and transport specific cargoes from the ER to Golgi. In Arabidopsis, ABA induces *AtSar1a* expression and the formation of AtSar1a-dependent giant COP Ⅱ vesicles (Li et al., 2021). To confirm whether the expression of *Sar1* and *SMG4* is also induced by ABA in rice, we tested the expression levels of *Sar1* and *SMG4* in seedlings of WT on ABA treatment. The expression levels of *Sar1* and *SMG4* were significantly repressed by ABA treatment in rice (Figure R2), indicating that there may be some differences in the function of Sar1 between rice and Arabidopsis under abiotic stress. Taken together, these observations suggest that SMG4 may not be involved in the formation of giant COP Ⅱ vesicles for stress responses in rice. Due to the lack of corresponding equipment and experimental technology in our lab, it is challenging for us to directly observe COP Ⅱ vesicles at this moment. However, we appreciate the reviewer's excellent point and like to perform experiments enabling direct observation of COP Ⅱ vesicles when possible in the future.

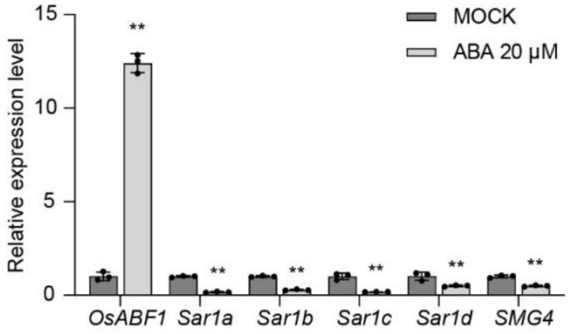

Figure R2 for Review only. *Sar1* and *SMG4* expression levels were determined by RT-qPCR in 5-day-old seedlings of WT under the ABA treatment.

The *OsABF1* and *UBIQUITIN* genes were used as positive and internal control, respectively. All data shown are mean ± SD (*n* = 3). Student's *t*-test was used to generate the *P* values, ***P* <0.01.

Minor points:

Q1. How many times of the magnification (according to the scale bar, should be 5 X Enlarged?) of the Figure 2F, 2G from Figure 2D, 2E? Please indicate in the legend or the figure.

Response: Thanks for your careful review. Figure 2F and 2G are 5× enlargement of the regions outlined by the box in Figure 2D and 2E, respectively. Accordingly, we indicated it in Figure 2 legend.

Q2. The value of the relative gene expression in WT should be 1 for all three replicates. Why there are error bar in WT samples in the Figure 2M, 2N?

Response: Thanks for your careful review. For each WT RNA sample, we performed three replicates and set their average value to 1. Then, we calculate the error relative to their average value using the data obtained by three replicates. Therefore, there are error bars in WT samples in the Figure 2M, 2N.

Q3. The heading of the Supplementary Figure 4 should be changed into "Knock out of SMG3 decreases grain size in both Kitaake and Nipponbare", since the data presented in the figure showing the decrement of grain size in the SMG3 CRISPR mutants.

Response: Thank you very much for your suggestion. We have changed the heading of the Supplementary Figure 4 to "Knock out of SMG4 decreases grain size in both Kitaake and Nipponbare".

Q4. Please indicate the band size in Figure 4D

Response: Thank you very much for your suggestion. We have added the band size in Figure 4D.

Q5. Please clarify the smg3-GFP mutant protein (P→L mutation on SMG3?) in Supplementary Figure 6C.

Response: Thank you very much for your advice. We have changed the smg4-GFP to SMG4^{P315L} (smg4)-GFP in Supplementary Figure 6C.

Reviewer #2:

In this manuscript "The MATE transporter Small Grain 3 increases grain size by mediating ER-Golgi transport of the CYP78A signal", the authors characterized a rice small grain mutant, small grain3(smg3), which has smaller grains than its wild type due to restricted cell expansion and cell proliferation in spikelet hulls. Using MAP based cloning, the authors successfully cloned SMG3, a multidrug and toxic compound extrusion (MATE) transporter. Loss of function of SMG3 resulted in smaller grains, while overexpressing SMG3 produced larger grains. SMG3 mainly co-localized with the endoplasmic reticulum exit sites (ERESs), partially co-localized with the ER and Golgi, and interacted with coat protein complex Ⅱ (COPⅡ) components, suggesting that SMG3 participates in COPⅡ-mediated ER-Golgi vesicle transport. SMG3 interacted with BG2, and acted in a common pathway with BG2 to regulate grain size. In addition, SMG3's homolog BIGE1A also interacted with BG2's homolog KLUH in Arabidopsis. The image quality is good with reasonable statistical analysis. The article is well organized with fluent English writing. However, the current data do not fully support the conclusion and the key mechanism needs to be addressed before considered to be published in Plant Cell.

Response: We greatly appreciate your recognition of our images and writing. All your comments are valuable and helpful for improving our manuscript. Please pay attention to the two changes in our revised version: “SMG3” to “SMG4”, as “SMALL GRAIN 3 (SMG3)” was recently used in a Plant Cell paper in December, 2022 (Li et al., 2022); the title is changed to “A CYP78As–SMG4–COPⅡ pathway promotes grain size in rice” for a clearer description on our findings.

Major concern:

Q1. Point 1. The major conclusion of the article "SMG3-COPⅡ likely mediates ER-Golgi vesicle transport of the CYP78A signal, revealing a novel BG2 (KLU)-SMG3 (BIGE1A)-COPⅡ regulatory pathway of grain (seed) size in plant." (line 43-45, line 111-114), is not fully supported by the experimental data.

To prove this, the authors need to provide two aspects of evidence. First, the authors need to prove that SMG3 indeed works together with the COPⅡ components to facilitate ER-Golgi vesicle transport. However, at current stage, the authors only showed that SMG3 colocalizes and interacts with the COPⅡ components in protoplast or in tobacco infiltration assays. Does the smg3 knock-out mutants disrupt ER-Golgi transport? Does the smg3 knock-out mutants affect ER-Golgi transport dependent on COPⅡ component? Cargo analysis in the knock-out mutants and over-expression lines is necessary to answer the question.

Response: Thank you very much for the key suggestion. To analyze whether SMG4 affects COPⅡ-mediated ER–Golgi transport, we examined the localization of the vacuolar cargoes Aleurain-mCherry (Zeng et al., 2015) and CYSP-mCherry (Delgadillo et al., 2020) as well as the plasma membrane cargoes SCAMP1-GFP (Cai et al., 2011) and PIP2;7-GFP (Hachez et al., 2014) , which were transported by COPⅡ, in protoplasts of Kitaake, *SMG4*-CR#1, and *SMG4*-OE#3, respectively. Sar1 dominant-negative mutant (Sar1DN) has been reported to inhibit ER-Golgi transport in plant cells (Zeng et al., 2015). We co-transformed the Sar1cDN-flag with cargoes in protoplasts as control. The result showed that the vacuolar cargoes (Aleurain-mCherry and CYSP-mCherry) and the plasma membrane cargoes (SCAMP1-GFP and PIP2;7-GFP) were delivered normally to the vacuole and plasma membrane in both *SMG4*-CR#1 and *SMG4*-OE#3, respectively, while Sar1cDN-flag significantly inhibited their transport (Supplemental Figure S11), indicating that COPⅡ-mediated ER–Golgi secretion route is not obviously disrupted in both *SMG4*-CR#1 and *SMG4*-OE#3. These data suggest that SMG4 likely does not affect the general transport function of COPⅡ. (Line 261-274).

Since other pathways that medicate ER–Golgi transport independent of COPⅡ are still largely unknown in plant (Barlowe and Helenius, 2016; Chung et al., 2016), we are currently unable to confirm whether SMG4 affects other ER–Golgi transport pathways.

To further explore the relation between SMG4 and COPⅡ, we treated *SMG4*-GFP transgenic seedlings with H89. H89 is a serine/threonine kinase inhibitor that specifically abolishes membrane recruitment of Sar1, thereby inhibiting COPⅡ assembly (Zeng et al., 2021). The result showed that the puncta labeled by SMG4-GFP significantly decreased with H89 treatment compared with the untreated control (Figures 6A and 6B), suggesting that the localization of SMG4 likely depends on COPⅡ. (Line 241-248).

For secretory cargoes, efficient sorting into COPⅡ carriers depends on transmembrane receptors that physically link cargo with coat subunits (Barlowe and Helenius, 2016). In this study, we found that *SMG4* encodes a MATE transporter with twelve transmembrane domains (Figure 3 and Supplemental Figure S5A), which is mainly localized to ERESs and partially localized to the ER and Golgi (Figure 5). And its localization pattern likely depends on COPⅡ (Figures 6A and 6B). SMG4 interacts with COPⅡ components (Figure 6C and Supplemental Figures S8 and S9), but does not affect the general COPⅡ transport function (Supplemental Figure S11). These characteristics are reminiscent of a canonical cargo receptor that links the ER lumenal cargo to the COPⅡ coat (Barlowe and Helenius, 2016). Therefore, we proposed that the transmembrane protein SMG4 might function as a COPⅡ-associated cargo receptor and transport specific cargo from the ER to Golgi (discussed in Line 359-369). However, we would also appreciate insightful comments from you on the function of SMG4.

Q1. Point 2. Second, the authors need to prove that the CYP78A signal is indeed the downstream effector and/or the candidate cargo of the SMG3-COPⅡ dependent ER-Golgi transport module. However, almost no evidence is presented at the current stage. The authors only show that SMG3 interacts with BG2 at protein level and in a common genetic pathway to regulate grain size. Does the subcellular localization of BG2 or any of the CYP78A signaling components change in the background of smg3 mutants and COPⅡ mutants? If there is no such evidence, how can they get the conclusion that the CYP78A signaling really depends on the ER-Golgi transport pathway?

Without detail mechanism to address the above-mentioned questions, the major conclusion would be an overstatement.

Response: Thank you very much for the key comments. To verify whether CYP78As (BG2, GL3.2, and BG2L1) are the cargoes transported by SMG4, we tested the subcellular localization of BG2, GL3.2, and BG2L1 in protoplasts of Kitaake, *SMG4* mutant and *Sar1*-Ri line, and found that BG2, GL3.2, and BG2L1 are all localized to the ER in both *SMG4*-CR#1 and *Sar1*-Ri-1 as in Kitaake (Supplemental Figure S22), indicating that CYP78As are not the cargoes transported by SMG4 (Line 396-400). Nevertheless, the transcript levels of *CYP78As* were significantly repressed in the *SMG4* mutants and *Sar1*-Ri-1, suggesting that there is an evident feedback regulation between CYP78As and SMG4–COPⅡ (Figure R3 and Supplemental Figure S19).

Although the nature of the CYP78A-derived signal is still unknown, we tried to confirm the relationship between *CYP78As* and *SMG4* by constructing genetic materials and analyzing their phenotypes. First, we knocked out *BG2* in the Kitaake background. The grain size of *BG2* knockout lines was comparable with that of Kitaake, which may be due to the functional redundancy between *BG2* and its homologous genes (Supplemental Figure S17). Further, we overexpressed *BG2* in the Kitaake background. The *BG2* overexpression lines produced larger grains than Kitaake (Figures 7E–7I), suggesting that *BG2* promotes grain size. To determine the genetic relationship between SMG4 and BG2, we overexpressed *BG2* in an *SMG4* knockout mutant *SMG4*-CR#1 background and found that the grain length, width, and thickness of *SMG4*-CR#1 were largely rescued (Figures 7E–7I), which suggest that *SMG4* acts, at least in part, in a common pathway with *BG2* to regulate grain size (Line 308-320).

Phylogenetic analysis and RT-qPCR analysis showed that *GL3.2* and *BG2L1* may be functionally redundant with BG2 (Supplemental Figures S17F and S17G). LCI assays and Co-IP assays showed that SMG4 also interacts with GL3.2 and BG2L1 (Figures 8A–8C). Based on the highly similar coding sequence among *BG2*, *GL3.2*, and *BG2L1*, we constructed RNAi vector to knock down *CYP78As* using RNAi technology in the Kitaake and *SMG4*-OE#3 background (Supplemental Figure S18). These results showed that two *CYP78A*-RNAi lines exhibited smaller grains compared with Kitaake (Figures 8D–8G), and down-regulation of *CYP78As* expression in the *CYP78A*-Ri/*SMG4*-OE#3 lines partially rescued the grain size of *SMG4*-OE#3 (Figures 8D–8G), indicating that *SMG4* acts, at least in part, in a common pathway with *CYP78As* to regulate grain size. (Line 321-336).

We agree with the reviewer that our current study does not fully demonstrate that the CYP78A-derived signal is the cargo of the SMG4–COPⅡ dependent ER-Golgi transport module. Thus, we revised our major conclusion as "A

CYP78As–SMG4–COP Ⅱ pathway promotes grain size in rice." We also conjectured that CYP78A-derived signal may be the cargo transported by SMG4–COP Ⅱ in the discussion section of the manuscript. (Line 396-415). We will continue to prove this inference in the future study. Although the cargo transported by SMG4–COP Ⅱ is still unknown, we established the relationship among CYP78As, SMG4, and COP Ⅱ in regulating grain size, which provides new insights into the molecular regulation mechanism of grain size in rice.

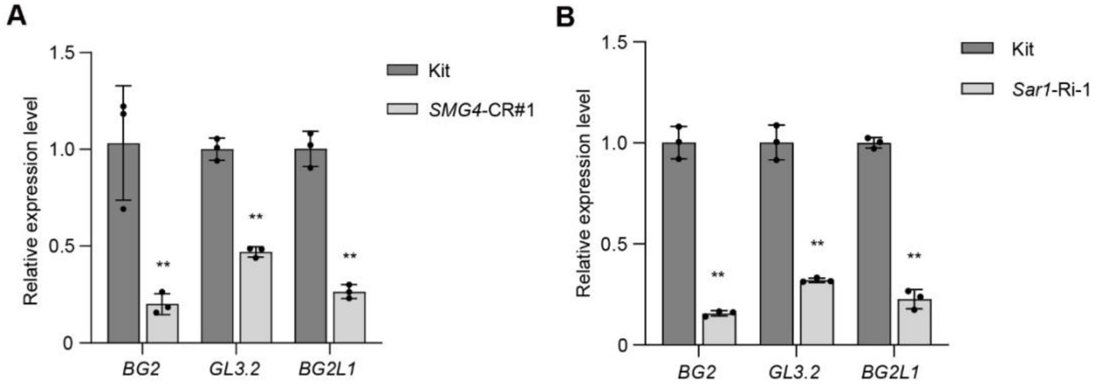

Figure R3 for Review only. The transcript levels of *CYP78As* are repressed in *SMG4*-CR#1 and *Sar1*-Ri-1

(A) and **(B)** Transcript levels of *CYP78As* (*BG2*, *GL3.2*, and *BG2L1*) in spikelet hulls of *SMG4*-CR#1 **(A),** and *Sar1*-Ri-1 **(B)**. The *UBIQUITIN* gene was used as an internal control. All data are mean ± SD (*n* = 3). Student's *t*-test was used to generate the *P* values, ***P* <0.01.

Q2. The authors put SMG3 in the same pathway with COP Ⅱ mediated ER-Golgi transport pathway. Is there any evidence showing that COP Ⅱ components regulate seed size? Either in Arabidopsis or in rice? Ideally, the double mutants of smg3 and COP Ⅱ mutants are required to prove this. If they cannot provide the genetic material, the conclusion should be weakened, as at current stage, only interaction and colocalization data are shown.

Response: Thank you very much for the key comment. The rice genome contains four Sar1 genes including *Sar1a*, *Sar1b*, *Sar1c*, and *Sar1d*. Previous study has shown that highly homologous Sar1a, Sar1b, and Sar1c are functionally redundant in rice endosperm (Tian et al., 2013). To analyze whether COP Ⅱ components are involved in regulating grain size, we generated *Sar1* knockdown lines with RNA interference technology in the Kitaake background (Supplemental Figure S12A). The RNAi vector was constructed using a highly similar coding sequence among *Sar1a*, *Sar1b*, and *Sar1c*. As expected, the *Sar1*-RNAi lines indeed exhibited smaller grains compared with those of Kitaake (Figures 6D and 6E), indicating that Sar1s indeed play an important role in grain size regulation in rice. To analyze the genetic relationship between *SMG4* and *Sar1s*, we knocked down *Sar1s* in the *SMG4*-CR#1 background using the RNA interference method (Supplemental Figure S12B), and found that the grain size of two *Sar1*-Ri/*SMG4*-CR#1 lines was obviously reduced compared with that of *SMG4*-CR#1 (Figures 6D and 6E). Therefore, these results support the notion that SMG4 interacts with COP Ⅱ components to regulate grain size. (Line 275-289).

Minor points:

Point 1. LCI assays showed that SMG3 interacts with the COP II components Sar1a, Sar1b, Sar1c, Sec23a, Sec23b, and Sec24a but not with Sec23c, Sec24b, and Sec24c (Figures 6D-6I, Supplemental Figure S8). They further verified these interactions by performing bimolecular fluorescence complementation (BiFC) assays in *N. benthamiana* leaf epidermal cells (Supplemental Figure S9) and co-immunoprecipitation (Co-IP) assays (Figures 6J and 6K) in rice protoplasts. The authors confirmed that SMG3 interacts with sar1a, Sar1b, sar1c, sec23a, sec23b, and sec24a, but not with sec23c, sec24b, and sec24c by LCI experiment. However, in BiFC experiment and CoIP experiment, the author only made the combination of interaction between SMG3 and sar1a, Sar1b, sar1c, sec23a, sec23b, and sec24a, but did not make the combination with SMG3 and sec23c, sec24b, and sec24c. So, the results are not rigorous and convincing.

Response: Thanks for your valuable suggestions. To verify whether SMG4 interacts with Sec23c, Sec24b, and Sec24c, we performed BiFC assays in *N. benthamiana* leaf epidermal cells (Supplemental Figure S9) and Co-IP assays in rice protoplasts (Figure 6C). However, these results showed that SMG4 interacts with Sec23c, Sec24b, and Sec24c (Supplemental Figure S9 and Figure 6C), which is inconsistent with our previous LCI results. To further confirmed the LCI results, we re-performed LCI assays.

In previous LCI assays, we ligated the coding sequences (CDSs) of *Sec23c*, *Sec24b,* and *Sec24c* to the N-terminal fragment of luciferase (nLUC) and ligated the CDS of *SMG4* to the C-terminal fragment of luciferase (cLUC) to perform the experiments (Figure R4). Alternatively, we re-ligated the CDSs of *Sec23c*, *Sec24b,* and *Sec24c* to the cLUC and re-ligated the CDS of *SMG4* to the nLUC to re-perform the LCI assays. The new LCI assays showed that SMG4 also interacts with Sec23c, Sec24b, and Sec24c while the LCI assays using the previous combinations failed to detect these interactions (Figure R4 and Supplemental Figure S8). These results suggest that the different fusions of genes to N- or C-terminal fragment of luciferase likely cause different results in LCI assays.

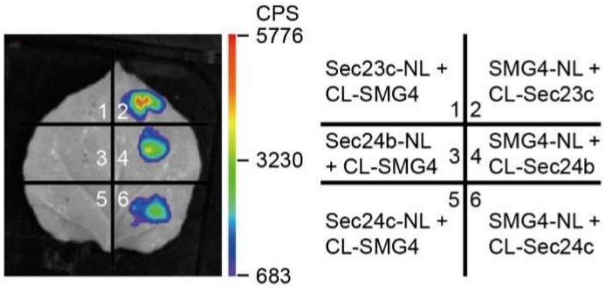

Figure R4 for Review only. LCI assays confirming the interactions between SMG4 and Sec23c, Sec24b or Sec24c in *N. benthamiana* leaves cells.

CL, C terminus of LUC; NL, N terminus of LUC. Colored scale bar indicates the luminescence intensity in counts per second (CPS).

Point 2. Since the yeast two hybrid (Y2H) experiment is done in Figure 7, why not do the Y2H experiment between SMG3 and COP Ⅱ components at the beginning?

Response: Thanks for your careful review. We performed the Yeast two-hybrid experiment (Y2H) between SMG4 and COPⅡ components. However, the interactions between them could not be detected in Y2H experiment (Figure R5). We speculate that there are two possible reasons for this result. The first possible reason is that the Y2H system used in this experimental system may not be suitable for detecting their interactions because SMG4 is a transmembrane protein located in the ER while the COPⅡ components are proteins recruited from the cytoplasm. The second possible reason is that their interactions may require the participation of other proteins, which do not exist in yeast.

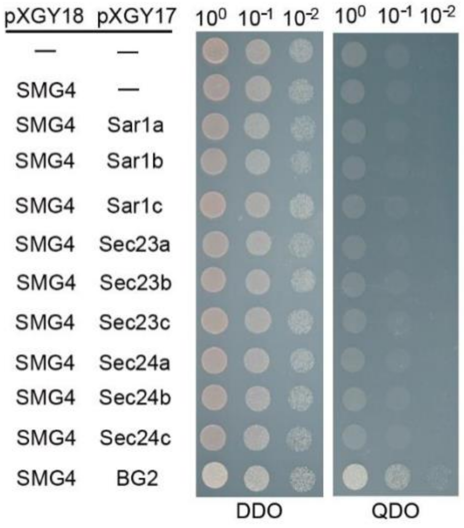

Figure R5 for Review only. Yeast two-hybrid assay between SMG4 and COP Ⅱ components. DDO, SD/-Trp/-Leu; QDO, SD/-Trp/-Leu/-His/-Ade. BG2 was used as a control.

Point 3. In addition, I didn't find the combination of YN + YC-SMG3 in supplemental Figure S9.

Response: Thanks for your careful review. We have added the combination of Y^N + Y^C-SMG4 in Supplemental Figure S9.

<u>Reviewer #3</u>:

The breeding goals are to increase grain yield in rice, however, the improvement of grain size and grain number is a major challenge for modern agriculture because of the well-established negative correlation between these two traits. In this manuscript, the authors identified a rice small grain mutant smg3, and that is caused by the mutation of a gene encoding MATE transporter. The further experiments revealed that SMG3 could interact directly with BG2, and mediates ER-Golgi vesicle transport of unknown mobile growth signal derived from BG2. The topic of this manuscript is interest for researchers follow trends and understanding of molecular mechanism of the genetic control of grain size and yield. However, there are deficiencies in experimental analysis that require substantiation before the manuscript can be acceptable for publication.

Response: Thank you very much for your appreciation of our research direction. All comments are valuable and helpful for improving our manuscript. Please pay attention to the two changes in our revised version: "SMG3" to "SMG4", as "SMALL GRAIN 3 (SMG3)" was recently used in a Plant Cell paper in December, 2022 (Li et al., 2022); the title is changed to "A CYP78As–SMG4–COP Ⅱ pathway promotes grain size in rice" for a clearer description on our findings.

Q1. in the current version of the manuscript, the authors proposed that SMG3-COP Ⅱ likely mediates ER-Golgi vesicle transport of the BG2/CYP78A signal, and the BG2-SMG3-COP Ⅱ regulatory module positively regulate grain size. However, the authors lack supporting evidences for this genetic association. In the bg2 mutant background, does overexpression of SMG3 cause the increase in grain size? In the smg3 mutant background, does overexpression of BG2 affect grain size?

Response: Thank you very much for the suggestion. Previous studies have shown that gain-of-function of *BG2* mutant exhibits larger grains (Xu et al., 2015). To verify the function of *BG2* in regulating grain size in rice, we first knocked out *BG2* in the Kitaake background. The grain size of *BG2* knockout lines was comparable with that of Kitaake, which may be due to the functional redundancy between *BG2* and its homologous genes (Supplemental Figure S17). Further, we overexpressed *BG2* in the Kitaake background. The *BG2* overexpression lines produced larger grains than Kitaake (Figures 7E–7I), suggesting that *BG2* does promote grain size. To determine the genetic relationship between *SMG4* and *BG2*, we overexpressed *BG2* in the *SMG4* knockout mutant *SMG4*-CR#1

background, and found that the grain length, width, and thickness of *SMG4*-CR#1 were largely rescued (Figures 7E–7I), which suggested that *SMG4* acts, at least in part, in a common pathway with *BG2* to regulate grain size (Line 308-320).

Phylogenetic analysis and RT-qPCR analysis showed that *GL3.2* and *BG2L1* may be functionally redundant with *BG2* (Supplemental Figures S17F and S17G). Protein interaction analysis showed that SMG4 also interacts with GL3.2 and BG2L1 (Figures 8A–8C). To verify the genetic relationship between *SMG4* and *CYP78As* (*BG2, GL3.2*, and *BG2L1*), we selected highly similar coding sequence among *BG2*, *GL3.2*, and *BG2L1* to construct RNAi vector to knock down *CYP78As* in the Kitaake and *SMG4*-OE#3 background (Supplemental Figure S18). And we found that two *CYP78A*-RNAi lines exhibited smaller grains compared with Kitaake (Figures 8D–8G) and down-regulation of *CYP78As* expression in the *CYP78A*-Ri/*SMG4*-OE#3 lines partially rescued the grain size of *SMG4*-OE#3 (Figures 8D–8G), which indicates that *SMG4* acts, at least in part, in a common genetic pathway with *CYP78As* to regulate grain size (Line 321-336).

We also supplement the genetic data between SMG4 and COP Ⅱ component. For details, please see our response to Reviewer 2, Question 2. (Line 275-289).

Q2. in Fig. 4D, which antibody used for analysis should be listed in the methods or figure. The N- and C-terminal GFP tags were completely digested by proteinase K. This result indicates that the N- and C-terminal of SMG3 is not in the membrane, how to confirm that they are exposed to the cytosol and not exposed to the lumen?

Response: Thank you very much for your careful review. We used GFP antibody for Western blot analysis and have listed it in Figure 4D and its legend. The protease K digestion assay was performed according to previous studies (Wang et al., 2011; Wang et al., 2016). Briefly, in the presence of proteinase K, ER membrane will protect luminal GFP from cleavage, and GFP will be digested if exposed to the cytosol. In our study, the N- and C-terminal GFP tags were completely digested by proteinase K (Figure 4D). Therefore, we think that both termini of SMG4 are exposed to the cytoplasm (Figure 4E).

Q3. the *smg3* mutant exhibited the reduced plant height, how about the overexpression lines?

Response: Thank you very much for your comment. *SMG4* overexpression lines exhibited higher plant height, compared with Kitaake (Supplemental Figures S3A-S3C). (Line 189)

Q4. Fig. 2H, which orientation for the cell number should be listed in the figure or legend.

Response: Thank you very much for the suggestion. We used black double-headed arrows to indicate the orientation in Figure 2A for the cell number statistics in Figure 2H, and annotated it in the legend.

Q5. Line155, Line159, Line 168 etc., "F2 population" should be "F_2 population".

Response: Thank you very much for your advice. We have changed the "F2 population" to "F_2 population".

References specific for Reviewer only

Barlowe C, Helenius A (2016) Cargo capture and bulk flow in the early secretory pathway. Annu Rev Cell Dev Biol 32: 197-222.

Cai Y, Jia T, Lam SK, Ding Y, Gao C, San MW, Pimpl P, Jiang L (2011) Multiple cytosolic and transmembrane determinants are required for the trafficking of SCAMP1 via an ER-Golgi-TGN-PM pathway. Plant J 65: 882-896.

Chung KP, Zeng Y, Jiang L (2016) COPII paralogs in plants: functional redundancy or diversity? Trends Plant Sci 21: 758-769.

Delgadillo MO, Ruano G, Zouhar J, Sauer M, Shen J, Lazarova A, Sanmartin M, Lai LTF, Deng C, Wang P, et al. (2020) MTV proteins unveil ER- and microtubule-associated compartments in the plant vacuolar trafficking pathway. Proc Natl Acad Sci U S A 117: 9884-9895.

Hachez C, Laloux T, Reinhardt H, Cavez D, Degand H, Grefen C, De Rycke R, Inze D, Blatt MR, Russinova E, et al. (2014) Arabidopsis SNAREs SYP61 and SYP121 coordinate the trafficking of plasma membrane aquaporin PIP2;7 to modulate the cell membrane water permeability. Plant Cell 26: 3132-3147.

Li B, Zeng Y, Cao W, Zhang W, Cheng L, Yin H, Wu Q, Wang X, Huang Y, Lau WCY, et al. (2021) A distinct giant coat protein complex II vesicle population in Arabidopsis thaliana. Nat Plants 7: 1335-1346.

Li J, Zhang BL, Duan PG, Yan L, Yu HY, Zhang LM, Li N, Zheng LY, Chai TY, Xu R, et al. (2022) An endoplasmic reticulum-associated degradation-related E2-E3 enzyme pair controls grain size and weight through the brassinosteroid signaling pathway in rice. Plant Cell.

Tian L, Dai LL, Yin ZJ, Fukuda M, Kumamaru T, Dong XB, Xu XP, Qu le Q (2013) Small GTPase Sar1 is crucial for proglutelin and α-globulin export from the endoplasmic reticulum in rice endosperm. J Exp Bot 64: 2831-2845.

Wang P, Hummel E, Osterrieder A, Meyer AJ, Frigerio L, Sparkes I, Hawes C (2011) KMS1 and KMS2, two plant endoplasmic reticulum proteins involved in the early secretory pathway. Plant J 66: 613-628.

Wang Y, Liu F, Ren Y, Wang Y, Liu X, Long W, Wang D, Zhu J, Zhu X, Jing R, et al. (2016) GOLGI TRANSPORT 1B regulates protein export from the endoplasmic reticulum in rice endosperm cells. Plant Cell 28: 2850-2865.

Xu F, Fang J, Ou S, Gao S, Zhang F, Du L, Xiao Y, Wang H, Sun X, Chu J, et al. (2015) Variations in *CYP78A13* coding region influence grain size and yield in rice. Plant Cell Environ 38: 800-811.

Zeng Y, Chung KP, Li B, Lai CM, Lam SK, Wang X, Cui Y, Gao C, Luo M, Wong KB, et al. (2015) Unique COPII component AtSar1a/AtSec23a pair is required for the distinct function of protein ER export in *Arabidopsis thaliana*. Proc Natl Acad Sci U S A 112: 14360-14365.

Zeng Y, Li B, Ji C, Feng L, Niu F, Deng C, Chen S, Lin Y, Cheung KCP, Shen J, et al. (2021) A unique AtSar1D-AtRabD2a nexus modulates autophagosome biogenesis in *Arabidopsis thaliana*. Proc Natl Acad Sci U S A 118: e2021293118.

TPC2022-RA-00268 1st Editorial decision –*acceptance pending* **Aug. 11, 2023**

We are pleased to inform you that your paper entitled "A CYP78As-SMG4-COP Ⅱ pathway promotes grain size in rice" has been accepted for publication in The Plant Cell, pending a final editorial review by a science editor.

Final acceptance from Science Editor **Aug. 30, 2023**
